# Multi-scale closed-loop tuning via spatial frequency collaborative sensitivity for rice leaf disease detection

**DOI:** 10.1371/journal.pone.0351727

**Published:** 2026-06-18

**Authors:** Yandong Song, Kang An, Lidong Wang, Bin Zhou

**Affiliations:** School of Engineering, Hangzhou Normal University, Hangzhou, Zhejiang Province, China; Communication University of Zhejiang, CHINA

## Abstract

Rice is a fundamental food source for more than half of the global population, making stable yields and quality improvements vital for food security and sustainable agricultural development. Early infections of rice leaf diseases often exhibit subtle symptoms, while conventional control methods based on empirical judgment and routine pesticide application result in both yield losses and environmental pollution. A Multi-scale closed-loop tuning via spatial frequency collaborative sensitivity (MCCA-YOLO) model has been proposed in this paper with a multiscale closed-loop tuning and spatial frequency collaborative attention mechanism for the early detection and classification of rice crop diseases. MCCA-YOLO incorporates a closed-loop tuning compound network architecture that combines a dual-backbone feature extractor with a spatial frequency enhancement module to achieve system self-verification feedback, reducing transmission errors and enhancing the texture features of leaves. The framework implements a cross-scale weighted fusion and a deformable spatial hybrid attention enhanced bidirectional feature pyramid fusion network for dynamic feature adaptation, effectively accommodating the complex morphology of rice leaf lesions. By conducting comprehensive ablation studies and comparative experiments with existing techniques on the rice plant diseases v8 dataset, the proposed approach achieves a mean average precision (mAP) of 92.2%, outperforming well-established methods, while delivering superior precision (0.915) and recall (0.900). Extensive empirical validation of additional v9 and Rice Leaf Spot Disease (RLSD) datasets for rice plant diseases further demonstrates the model’s outstanding performance.

## 1. Introduction

Rice is a fundamental cereal crop worldwide, serving as the primary dietary staple for a large portion of the global population. Maintaining high and stable rice yields is vital for global food security. However, rice plants are highly susceptible to various pathogenic infections during their growth cycle, including Rice Blast (Magnaporthe oryzae), Grassy Stunt virus, Bacterial Leaf Blight (Xanthomonas oryzae), and Tungro disease [1]. These phytopathological conditions significantly reduce grain production and degrade rice quality parameters, resulting in substantial economic losses within agricultural systems. Timely and accurate detection methods for rice leaf diseases are essential for developing effective management strategies and targeted control measures [2].

Traditional rice leaf disease detection methods primarily rely on visual inspection and empirical assessment by agricultural experts. This approach has significant limitations, including low operational efficiency, high subjectivity, and limited capability in identifying early-stage disease symptoms. These constraints make it inadequate for large-scale rice cultivation systems. In recent years, the advancement of deep learning has facilitated the integration of Convolutional Neural Networks (CNNs) [3] and attention mechanisms [4] into plant disease identification. While these technological approaches have shown promise in rice leaf analysis, several critical challenges remain. Deep learning techniques have demonstrated remarkable success in image recognition, offering innovative approaches for detecting diseases in rice foliage.

Deep learning models based on Convolutional Neural Networks (CNNs) can automatically extract high-level features from medical images, eliminating the need for manual feature engineering and significantly improving pathological condition detection accuracy. Deari et al [5] proposed a hybrid multi-stage model integrated with an enhanced Inception network for rice leaf disease classification, demonstrating superior detection performance compared to conventional machine learning approaches. Additionally, Kurmi et al. [6] proposed a deep convolutional neural network (CNN) that enhances leaf disease detection accuracy by eliminating redundant information and utilizing color attributes. The proposed methodology proves equally effective in the context of rice leaf disease identification, yielding notable improvements in the precision of pathological recognition. Furthermore, the incorporation of attention mechanisms and multi-scale feature fusion has emerged as a crucial strategy for optimizing detection performance. Zhao et al. [7] proposed the AC-YOLO model, an enhanced YOLOv7-based algorithm that incorporates the Convolutional Block Attention Module (CBAM), Self-Attention and Convolution (ACmix), and the Efficient Complete Intersection over Union (ECIoU) loss function. This framework significantly improves both detection accuracy and generalization in grain pest identification tasks. Li et al. [8] proposed a self-attention-based feature fusion model (SAFFPest) built upon Varifocal Net, enhancing rice pest detection accuracy through self-attention mechanisms and Group Normalization techniques. Kumar et al. [9] presented the RDTNet network based on multi-scale feature fusion, comprising two core modules. The first module extracts discriminative features from three distinct scales through Local Binary Pattern (LBP), grayscale, and Histogram of Oriented Gradients (HOG) image representations. The second module hierarchically integrates semantic global and local features. Tian et al. [10] proposed a V-Space-based multi-scale feature fusion SSD method (VMF-SSD), significantly improving apple leaf disease detection accuracy through multi-scale feature extraction and attention mechanisms.

Current approaches typically employ convolutional operations with fixed geometric configurations, limiting their adaptability to pathological pattern variations in plants under different growth stages and environmental conditions. While conventional attention mechanisms (e.g., channel or spatial attention) partially address feature selection challenges, they often exhibit a limited capacity to integrate multi-scale contextual information and preserve fine-grained local details simultaneously. Existing multi-scale fusion strategies frequently encounter information redundancy and scale conflicts, particularly when processing early-stage subtle symptoms on rice leaves, where symptom manifestations exhibit weak correlations across scales. To better accommodate morphological variations in plant diseases, a Multi-scale Constrained Deformable Convolution Network (MCDCNet) [11] is proposed, effectively detecting apple leaf diseases through a multi-branch architecture and deformable convolution techniques. The MCDCNet captures disease features across multiple scales and dynamically adjusts convolution kernel shapes through deformable convolution operations, enabling adaptive feature extraction aligned with lesion morphology characteristics.

Inspired by previous research, we propose a deep neural network model named Multi-scale closed-loop tuning via spatial frequency collaborative sensitivity for rice leaf disease detection (MCCA-YOLO), which is specifically developed for the identification of rice leaf diseases. The proposed detection network consists of a closed-loop tuning Bi-backbone [12] feature extraction network for system self-verification feedback, and a multi-scale feature weighted and deformable hybrid attention bidirectional feature pyramid [13] fusion network to achieve adaptive extraction and cross-scale fusion of rice leaf lesion features. The model implements a closed-loop feedback control system by connecting the feature fusion network output to the auxiliary backbone network, enabling dynamic adjustment of feature learning through feedback signals. The contributions of this work can be outlined as follows:

1. We propose a bidirectional feature pyramid fusion network for neck feature fusion, which combines cross-scale weighted fusion with deformable spatial hybrid attention enhancement. It dynamically adjusts the fusion weights of multi-scale features, adapts to the deformation and texture direction of rice leaves, and effectively improves the detection ability of complex shapes of rice leaf lesions.
2. We propose a closed-loop tuning composite network architecture that combines a Bi-backbone feature extractor with a two-stage spatial frequency feature enhancement module. This architecture dynamically adjusts the feature extraction process of the dual-backbone network based on the feedback signals from the neck feature fusion. Through the closed-loop feedback mechanism, it reduces error propagation, enhances the texture and edge details of rice leaves, and improves their feature representation.
3. We conduct extensive experiments on different real-world rice leaf datasets. The experimental results show that our method outperforms other state-of-the-art approaches. The model is deployed for inference applications on edge devices. Our code is publicly accessible as open source at: https://github.com/sstan12/MCCA-YOLO.

The remainder of this paper is organized as follows. Section 2 reviews prior research on object detection, attention mechanisms, and rice leaf disease detection. Section 3 presents the proposed MCCA-YOLO detection network in detail, including its overall architecture, deformable hybrid collaborative attention mechanism, two-stage spatial frequency enhancement module, closed-loop tuning dual-backbone network, and scale-weighted fusion network. Section 4 evaluates the algorithm on several rice leaf disease datasets and discusses the experimental results. Finally, Section 5 summarizes the main findings and concludes the paper.

## 2. Related work

### 2.1. Backbone and neck architectures for object detection

Object detection algorithms are typically categorized into two main types: two-stage and one-stage approaches. Two-stage techniques, exemplified by Fast R-CNN [14], initially create region proposals, which are refined in a subsequent phase. These methods often deliver high precision at the expense of processing speed. On the other hand, one-stage detectors perform localization and classification in a single forward pass for real-time efficiency. Representative one-stage detectors include SSD [15] and the YOLO series [16], while Mask R-CNN [17] is a notable two-stage extension of the Faster R-CNN framework. Modern detector designs follow a common architecture: a backbone network (typically a pre-trained classification CNN) extracts basic features, a neck network fuses multi-scale features such as FPN [18], and a detection head predicts bounding boxes and class scores. Among one-stage detectors, the YOLO (You Only Look Once) series is a cornerstone due to its balance of accuracy and speed, with YOLOv8 [19] being one of the most widely adopted models in industrial applications. It adopts an improved architecture featuring an anchor-free detection head and a CSPNet-based backbone with an FPN [18]+PAN [20] neck, achieving higher accuracy and faster inference than its predecessors.

Investigations into detection backbones have primarily focused on two key strategies: designing deeper or wider networks to extract stronger features, and introducing multi-branch architectures to enhance information fusion. The first strategy involves the historical use of increasingly deep classification networks (e.g., VGGNet [21], ResNet [22]) as backbones for detection, which improves accuracy but with diminishing returns and higher computational demands. The latter strategy seeks to increase representational capacity not through depth, but via architecture. A prominent example is the Composite Backbone Network (CBNet) [12]. Experiments have shown that a detector with two fused ResNet50 backbones (CBNet) outperforms a single ResNet101 backbone, yet with comparable model complexity. Inspired by the success of composite backbones, this work introduces a Closed-loop Tuning Mechanism Dual-Backbone architecture for YOLOv8. By coupling two YOLOv8 backbones in parallel and enabling feature-sharing between them, we aim to enhance the network’s feature diversity and expressive power.

In the model’s architecture, the backbone plays a crucial role in deriving hierarchical feature representations from the input data. These multi-scale features are then fused and enhanced by a neck module to form a more discriminative representation for detection. Feature pyramid fusion has emerged as a standard element in contemporary detectors. The pioneering Feature Pyramid Network (FPN) [18] introduced a top-down pathway with lateral connections to propagate strong semantic features from deep layers to higher-resolution features, significantly improving the detection of objects at various scales. PANet [20] augments FPN with an additional bottom-up path, shortening the information flow between low-level and high-level features and thus improving the representation of small objects. More recently, EfficientDet’s Bidirectional Feature Pyramid Network (BiFPN) [13] introduced a bi-directional feature pyramid with learnable fusion weights, facilitating efficient multi-scale feature fusion through iterative top-down and bottom-up processes. Despite BiFPN’s success in multi-scale feature fusion, it faces challenges in situations with significant feature deformation and strong cross-scale contextual dependencies. The proposed Scale-Weighted Fusion Network (SWFN) maintains PAN’s lightweight bidirectional structure while replacing BiFPN’s uniform scalar weights with a scale-specific weight matrix. This matrix assigns unique coefficients to each semantic channel at every scale, allowing the network to prioritize fine-grained details from lower levels whenever they are diagnostically significant. Simultaneously, the network leverages the robust context from higher levels, and this weighted approach provides SWFN with enhanced resilience to shape variability and improved utilization of cross-scale context.

### 2.2. Attention mechanism

In recent years, attention mechanisms have proven to be highly effective in enhancing features within convolutional neural networks (CNNs). They operate by adaptively weighting feature responses, emphasizing informative regions or channels while suppressing less useful ones, which has led to substantial gains in various vision tasks including object detection. The seminal Squeeze-and-Excitation (SE) network [23] models inter-channel dependencies, recalibrating channel-wise feature responses to boost representational capacity. This module reweights features based on the importance of different channels, highlighting those with stronger discriminative capabilities. However, the SE module neglects dependencies in the spatial dimension and thus cannot effectively model spatial feature interactions. To incorporate spatial information, the Convolutional Block Attention Module (CBAM) [24] sequentially applies channel and spatial attention sub-modules. Although it achieves improved performance, CBAM relies on relatively complex convolutional computations, increasing the computational burden of the model. To reduce computational complexity, Wang et al. introduced the Efficient Channel Attention (ECA) module in 2020 [25]. By replacing the fully connected layers in SE with a 1D convolution, ECA drastically reduces the number of parameters while retaining the ability to model inter-channel interactions, striking a favorable balance between efficiency and performance. In 2021, Hou et al. proposed Coordinate Attention (CA) [26], an approach that embeds precise positional information into channel attention by decomposing feature maps and encoding long-range dependencies along the two spatial directions. The resulting coordinate-aware descriptors fuse spatial and channel cues, enabling the network to generate spatially selective responses.

Despite these advances, handling significant geometric variations and capturing broader contextual dependencies remained challenging. The Contextual Transformer (CoT) block [27] integrates local contextual cues into the attention computation, thereby strengthening feature interactions and improving recognition. To better capture global patterns, researchers have turned to the frequency domain, complementing spatial attention mechanisms. Frequency Channel Attention (FCA) [28] employs the discrete cosine transform (DCT) to capture channel information across different frequencies, underscoring the importance of mining feature discrepancies in the frequency domain. Nevertheless, FcaNet’s primary focus on static image classification tasks may limit its effectiveness in addressing the integration of spatial and frequency domain information in object detection scenarios, where features are more intricate and vary across multiple scales. In 2025, Zhang et al. proposed the Unmanned Aerial Vehicle-DETR (UAV-DETR) model [29], which includes a multi-scale feature fusion and frequency-enhancement module capable of capturing spatial and frequency information at different scales. Experimental results demonstrate that this approach achieves significant performance gains on UAV image datasets, such as VisDrone [30]. Frequency Dynamic Convolution (FDConv) [31] learns filters with diverse frequency components to capture multi-frequency patterns more effectively. These frequency-driven approaches can encode fine-grained textures but inherently operate on globally transformed features, limiting their ability to model spatial interactions. Consequently, hybrid strategies have been proposed to integrate spatial and frequency information. A notable example is the dual-branch transformer in SFHformer [32], which synthesizes local spatial-domain features with global frequency domain representations derived from the Fast Fourier Transform (FFT). Other works explore hybrid attention in different architectures. For instance, the FSTA SNN [33] uses spectral statistics to guide spatial and temporal attention, though it is designed for spiking neural networks and operates at a single scale. However, when handling the complex backgrounds and variable targets found in rice leaf disease scenarios, the model still faces limitations in feature representation.

In spite of the notable progress mentioned earlier, there are still several unresolved issues. Many current methods rely on predetermined spatial sampling points, making them less effective or inadequate when faced with significant geometric changes. Furthermore, prevalent attention mechanisms often employ fixed or heuristic-based fusion strategies, failing to dynamically recalibrate feature responses based on the varying characteristics of different lesions and image contexts. These limitations ultimately restrict both the flexibility and generalization capabilities of current models. To overcome these challenges, we propose two core components: a Deformable Hybrid Collaborative Attention (DHCA) mechanism and a Two-Stage Spatial Frequency Enhancement (TSSFE) module. They are designed to address:

- Fine-grained extraction of lesion texture features during feature encoding.
- Adaptive modeling of spatial deformation in rice leaves to jointly enhance leaf texture and positional information.

These modules form the core of our proposed MCCA-YOLO framework, which is tailored for robust rice leaf disease detection.

### 2.3. Rice leaf pathological detection

In the field of plant disease detection, research on rice leaf disease object detection has seen notable progress in recent years, mainly due to breakthroughs in deep learning. In 2019, Kawcher Ahmed et al. [34] utilized machine learning techniques, including KNN [35] and Decision Trees [36], to detect three of the most common rice plant diseases, such as Black Smut, Bacterial Leaf Blight, and Brown Spot. However, these traditional methods are heavily based on hand-crafted features and predefined descriptors, limiting their adaptability and robustness under varied field conditions. Such limitations have driven subsequent research toward more robust deep learning-based detection frameworks. Subsequent work, like that of Sharma et al. [37], applied convolutional neural networks (CNNs) to detect a broader set of rice diseases and pests. While CNNs have demonstrated significant efficacy in detecting rice diseases, they still face challenges in practical scenarios, particularly in adapting to intricate backgrounds, generalizing from limited datasets, and ensuring computational efficiency. Researchers have increasingly adopted lightweight, UAV-compatible object detection models to enable real-time monitoring in variable field conditions. A notable example is UAV T-YOLO-Rice [38], a lightweight detector based on Tiny YOLOv4. It reported 86% mAP on several diseases and offered a favorable speed-accuracy trade-off, though its older architecture may not fully leverage recent advances. However, relying on the older YOLOv4 backbone restricts its ability to leverage modern advancements in anchor-free detection and multi-scale feature processing, potentially limiting performance on a wider range of diseases and more varied environmental factors.

To address these limitations, we propose MCCA-YOLO, an enhanced framework based on YOLOv8. Our primary objective is to achieve superior accuracy while maintaining high computational efficiency suitable for practical deployment. This is achieved through several key innovations integrated into the YOLOv8 architecture, designed specifically to enhance robustness against field complexities such as cluttered backgrounds and varying leaf geometries.

## 3. Methods

### 3.1. MCCA-YOLO model architecture

**Fig 1:**
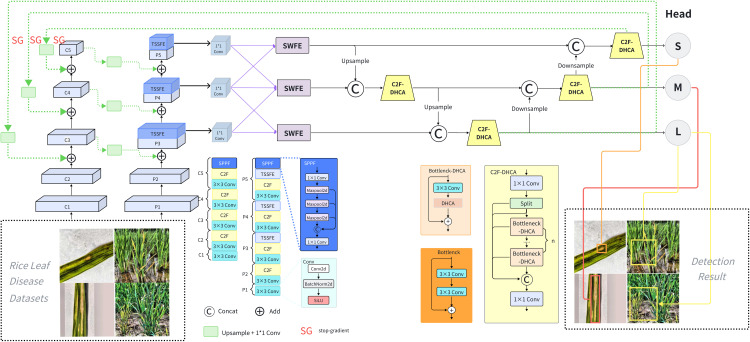
MCCA-YOLO model architecture.

This study proposes a novel framework named Multi-scale Closed-loop tuning via Collaborative spatial-frequency sensitivity Attention YOLO (MCCA-YOLO) for rice leaf disease detection. As shown in Fig 1, the cross-scale feature fusion network generates feedback features. These feedback features are utilized to calibrate the backbone network. This refinement process involves upsampling the features from the neck network and then applying a 1×1 convolutional projection on the relevant layers. The objective of this process is to align the details of the shallow layers with the semantics of the deep layers in the spatial dimension. The model integrates this alignment with the characteristics of the corresponding layers from the auxiliary backbone network. Following this closed-loop calibration, the calibrated values *C*_3_, *C*_4_, and *C*_5_ are upsampled, respectively, and then fed into the main backbone through a 1×1 convolutional layer. At the *P*_3_, *P*_4_, and *P*_5_ layers of the main backbone, a two-stage spatial frequency enhancement module (TSSFE) has been designed to emphasize the detailed texture information characteristic of leaf diseases.

The multi-scale features are then integrated in the Scale-Weighted Fusion Neck. Here, the Scale-Weighted Fusion Entity (SWFE) module performs adaptive weighted fusion of cross-layer features, explicitly learning the contribution weight of each scale to lesion detection. After the fusion process, the features are aggregated along alternating top-down and bottom-up paths. During cross-scale fusion, upsampling enhances spatial resolution, whereas downsampling reinforces global context. In the CSPDarknet53–2-Stage FPN (C2F) model, a deformable spatial hybrid collaborative attention (DHCA) mechanism is designed at each node to enhance the directionality and channel features of striped lesions adaptively. Three detection heads (S/M/L) correspond to small/medium/large objects, each predicting bounding boxes and class probabilities. This design enables the system to maintain a lightweight architecture while preserving high sensitivity to minor disease manifestations and ensuring robust performance in complex environments.

### 3.2. Deformable hybrid collaborative attention

**Fig 2:**
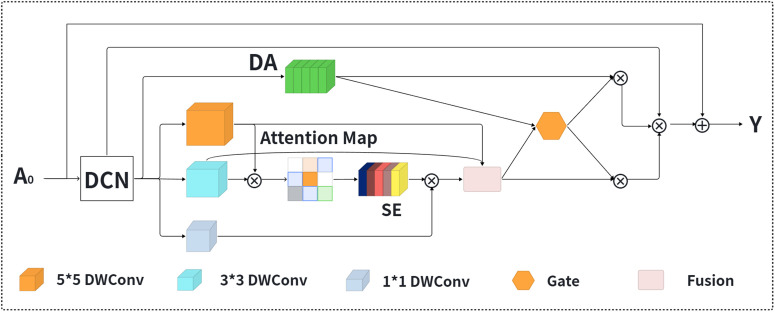
Deformable hybrid collaborative attention architecture.

When detecting rice leaf diseases in natural settings, the leaves themselves are often deformed due to wind and gravity, exhibiting significant bending and tilting. Lesion spots on leaves also tend to arrange in linear patterns, either along the leaf’s longitudinal axis or transverse to it. These flexible shape modifications and linear arrangements make it challenging to reconstruct the pattern of disease lesions accurately. Concurrently, diseased spots and healthy leaf veins frequently exhibit local textures that are highly similar, thereby impeding the efficacy of conventional spatial attention in reliably differentiating between them. To address these challenges, we propose a Deformable Hybrid Collaborative Attention (DHCA) mechanism and integrate it into the C2F component of the YOLOv8 feature fusion network, forming a new bottleneck-DHCA module, as illustrated in Fig 2. This module can pre-generate offsets utilizing a deformable convolution (DCN). Subsequently, it can dynamically adjust the sampling position of the convolution kernel. Following the DCN, the module incorporates two parallel branches: the direction-aware attention branch and the channel-self-attention branch. The features from both branches are fused via a learnable gating mechanism. Furthermore, residual connections are incorporated to stabilize gradient flow. This design enables the module to effectively adapt to the diverse shapes of leaves.

#### 3.2.1. Deformable convolution.

We introduce a Deformable Convolution v4 (DCNv4) [39] layer to perform geometric-adaptive alignment on the input features $A_{0}\in {\mathbb{R}}^{B\times C\times H\times W}$, enabling the network to more accurately capture oblique or curved rice-leaf lesions. The DCNv4 framework augments the conventional 2D convolution process by introducing a parallelisation approach to the prediction of learnable offset and modulation coefficients for each convolution kernel position. Consequently, the sampling grid can adaptively deform to fit the local geometric structure of the input, such as leaf curvature and vein orientation. To adapt the sampling locations, we augment the standard convolution with learnable offsets $\left\{\Delta p_{k}\right\}$ and modulation coefficients $m(p_{k})$. Thus, the output feature map A $\in {\mathbb{R}}^{B\times C\times H\times W}$ at spatial position *p*_0_ is computed as shown in Equation (1).


$$\begin{array}{c} A=\sum_{p_{k}\in \mathbb{R}}w(p_{k})\cdot A_{0}(p_{0}+p_{k}+\Delta p_{k}(p_{0}))\times m(p_{k}|p_{0}). \end{array}$$ (1)

*R* is a fixed (2R+1)×(2R+1) convolution kernel sampling grid, where *R* is the convolution radius. A lightweight convolutional sub-network predicts the offset $\Delta p_{k}(p_{0})$ for each sampling position. The kernel weight $w(p_{k})$ corresponds to the original coordinate $p_{k}$ and linearly transforms the features at the offset location. A parallel sub-network followed by a sigmoid activation generates the modulation coefficient $m(p_{k}|p_{0})$. Each modulation coefficient weights the corresponding transformed feature. Since the deformed sampling location ${𝐩}_{0}+{𝐩}_{k}+\Delta {𝐩}_{k}$ typically has non-integer coordinates, we use bilinear interpolation to compute its feature value from the four nearest pixels in the input feature map. These values are weighted according to the distance and added to obtain the values for the continuous positions.

#### 3.2.2. Directional attention.

**Fig 3:**
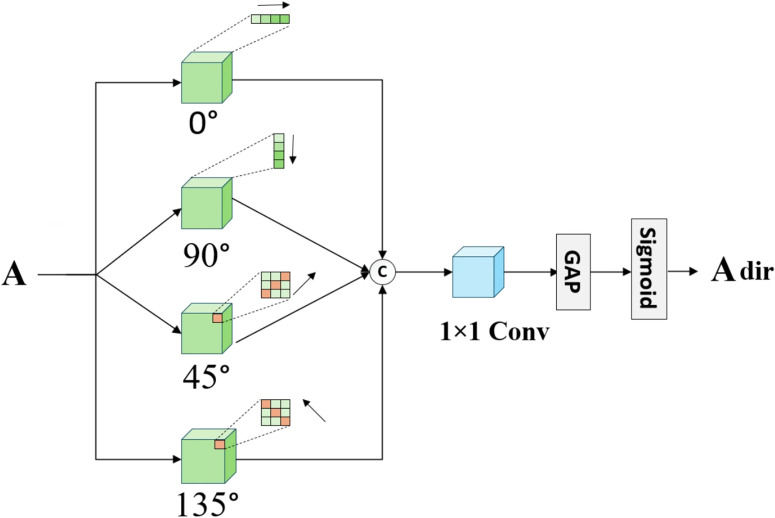
Direction attention architecture.

Following alignment via DCNv4 to compensate for leaf bending and tilting, the pixel positions in the feature maps are geometrically corrected. However, alignment alone is insufficient to capture the linear distribution of lesions along specific directions. To address this, we introduce a Directional-Attention (DA) module, which processes the aligned features using dedicated filters oriented horizontally, vertically, and diagonally, as shown in Fig 3. It explicitly embeds the growth-pattern cues of rice leaves, thereby strengthening the network’s response to lesion-related textures. One pathway, designated as the horizontal branch, employs a convolution kernel of size 1×3 to perform directional filtering across columns. The other path, the vertical branch, employs a 3×1 kernel to filter across rows, which significantly enhances the model’s ability to detect and represent features aligned either horizontally or vertically. For the diagonal approximation branches, 3×3 depth-separable convolutions with a dilation factor of 2 are employed. This design incorporates sampling neighborhoods spaced two pixels apart, resulting in sparser weights that concentrate on the 45° and 135° diagonal orientations. Formula (2) provides a comprehensive overview of the filtering responses exhibited by rice leaves in the horizontal, vertical, and diagonal directions.


$$\begin{array}{cc} F(0^{\circ }) & ={DWConv}_{1\times 3}(A),\\ F(90^{\circ }) & ={DWConv}_{3\times 1}(A),\\ F(45^{\circ }) & ={DWConv}_{3\times 3,d=2}(A),\\ F(135^{\circ }) & ={DWConv}_{3\times 3,d=2}(A). \end{array}$$ (2)

Information obtained from four distinct receptive fields and directional undergoes compression in the channel dimension, followed by concatenation and fusion, subsequently mapped from 4C to C through the utilisation of a 1×1 convolution to obtain $F_{\text{dir}}\in {\mathbb{R}}^{B\times C\times H\times W}$, As shown in formula (3).


$$\begin{array}{c} F_{\text{dir}}={\text{Conv}}_{1\times 1}(\text{Concat}[F(0^{\circ }),F(45^{\circ }),F(90^{\circ }),F(135^{\circ })]). \end{array}$$ (3)

We apply global average pooling over the spatial dimensions of *F*_dir_, producing a tensor of shape B×C×1×1. A subsequent sigmoid activation yields the directional-attention coefficients *A*_dir_ defined in (4). The design outlined above facilitates the model’s capacity to exhibit multi-scale and multi-directional feature perspectives with minimal computational expenditure, thereby enhancing the conspicuity of lesion edges and slender leaf veins.


$$\begin{array}{c} A_{dir}=\sigma \;\left(\frac{1}{HW}\sum_{i=1}^{H}\sum_{j=1}^{W}F_{dir}(i,j)\right). \end{array}$$ (4)

#### 3.2.3. Channel self-attention.

While the directional-attention component enhances the local textures found in leaf blades and veins, relying solely on these directional indicators is insufficient for accurately differentiating lesions from healthy veins. To address this issue, we propose a parallel multi-scale channel self-attention branch. This branch first models global inter-channel correlations to capture a holistic semantic context. This weighting vector is then used to recalibrate the input features, performing channel-wise scaling that can potentially amplify lesion-related features and suppress responses from healthy regions. Consider $A\in {\mathbb{R}}^{B\times C\times H\times W}$ as the input feature map. First, two complementary feature representations, $A_{k}$ and $A_{Q}$, are generated by applying depthwise separable convolutions with different kernel sizes (3×3 and 5×5, respectively) to capture multi-scale context, as defined in formula (5).


$$\begin{array}{c} A_{K}=DWConv_{3\times 3}(A),\;A_{Q}=DWConv_{5\times 5}(A). \end{array}$$ (5)

As demonstrated in Fig 2, the key map $A_{K}$ captures local lesion context within a 3×3 window, whereas the query map $A_{Q}$ aggregates a broader 5×5 window. $A_{K}$ and $A_{Q}$ are concatenated along the channel dimension and then passed to a two-layer 1×1 convolutional module for attention embedding. The first 1×1 convolution compresses the channel dimension from 2C to 2C/f (with a typical reduction ratio f = 4), followed by batch normalization and a ReLU activation. The second 1×1 convolution then expands the channel count to *n*^2^*C*, where n is the side length of a local n×n window. The output of this convolution represents the dynamic weight for each channel within its corresponding n×n local window. The output tensor is reshaped to ${\mathbb{R}}^{B\times C\times n^{2}\times H\times W}$, and subsequently averaged across all window positions along the *n*^2^ dimension. This process yields an attention map of shape B × C × H × W, which is denoted as *A*_avg_ and calculated according to formula (6).


$$\begin{array}{c} A_{\text{avg}}=\frac{1}{n^{2}}\;{\text{Mean}}_{m=1,\ldots ,n^{2}}\left\{\text{Reshape}\;\left({\text{Conv}}_{1\times 1}\;\left({\text{Conv}}_{1\times 1}\;\left([A_{K},A_{Q}]\right)\right)\right)\right\}. \end{array}$$ (6)

The term *Reshape* is used to denote the adjustment of the channel dimension from *n*^2^*C* to [*C*, *n*^2^]. As indicated by the numeral ${Mean}_{m=1,\ldots ,n^{2}}\{\}$, the process involves the averaging of the data at the third dimension position. This results in the generation of an attention map with a shape of ${\mathbb{R}}^{B\times C\times H\times W}$. After generating the attention map, we re-weight *A*_avg_ with the SE [23] channel coefficients and apply global average pooling across the spatial dimensions, producing a channel descriptor vector *z* defined in formula (7).


$$\begin{array}{c} z=\frac{1}{HW}\sum_{i=1}^{H}\sum_{j=1}^{W}A_{\text{avg}}(i,j).\; \end{array}$$ (7)

The channel weight vector is obtained through two fully connected layers and the Sigmoid function. Broadcasting this vector over the spatial dimensions produces the channel-attention map $A_{s}$, which highlights the most informative channels for the network, as illustrated in Formula (8).


$$\begin{array}{c} A_{s}=\sigma (W_{2}\delta (W_{1}z)).\; \end{array}$$ (8)

We denote the ReLU and Sigmoid activations by δ(·) and σ(·). Respectively, two fully connected layers with weights $W_{1}\in {\mathbb{R}}^{\frac{C}{r}\times C}$ and $\;W_{2}\in {\mathbb{R}}^{C\times \frac{C}{r}}\;$ first compress the channel dimension by a factor of r and restore it. The channel-attention map $A_{s}$ is subsequently employed to refine the feature representation. Specifically, the input feature map A is first projected through a 1×1 convolution to obtain the feature representation $A_{v}\in {\mathbb{R}}^{B\times C\times H\times W}$. The channel-attention map is applied $A_{s}$ to $A_{v}$ through element-wise multiplication, producing the refined features *V*_weight_. This operation is summarized in formula (9).


$$\begin{array}{c} V_{\text{weight}}=A_{s}⊙A_{v}. \end{array}$$ (9)

The symbol ⊙ is employed to denote numerical multiplication at the same index position. In the process of designing the feature fusion module, the two feature streams $A_{K}$ and $A_{Q}$ are concatenated in parallel in the channel dimension, thereby yielding the shape [B, 2C, H, W]. Subsequently, the number of channels is remapped from 2C to C via a 1×1 convolution. This operation fuses the features extracted by the 3×3 depth convolution of $A_{K}$ and the 5×5 depth convolution of $A_{Q}$, integrating the two parts of information into the C channel space to obtain the fused feature *F*_fuse_, denoted as formula (10).


$$\begin{array}{c} F_{\text{fuse}}={\text{Conv}}_{1\times 1}(\text{Concat}(A_{K},A_{Q})). \end{array}$$ (10)

We aggregate *V*_weight_ and *F*_fuse_ with global average pooling, then add them element-wise to obtain the final output of the multi-scale channel self-attention branch *A*_channel_, denoted as formula (11). This summation combines the convolution’s local-context features with the inter-channel filtering cues provided by the SE mechanism, thereby uniting spatial-structure sensitivity with adaptive channel selection in a shared feature space.


$$\begin{array}{c} A_{channel}=GAP({𝐅}_{fuse}+V_{weight}).\; \end{array}$$ (11)

#### 3.2.4. Cross-gating mechanism.

We introduce a learnable gating mechanism that adaptively balances directional attention and global-channel attention according to the strength of each channel’s response. The outputs of the two branches, denoted as *A*_dir_ and *A*_channel_, are first concatenated. The combined tensor is then fed into a gating function gate. The gating function consists of a 1×1 convolution followed by a Sigmoid function, thereby producing a gating tensor *G*, as demonstrated in Formula (12).


$$\begin{array}{c} G=\sigma ({Conv}_{1\times 1}({Concat}_{c}(A_{dir},\;A_{channel})))\;. \end{array}$$ (12)

Each element of the gate tensor $G\in {\mathbb{R}}^{B\times C\times H\times W}\;$ lies in the range [0,1], it acts as a balance coefficient between directional and contextual attention for channel C in batch B, the gate fusion process utilizes the gate tensor *G* to generate two complementary attention maps. As shown in formula (13).


$$\begin{array}{c} A_{channel}^{*}=A_{channel}\;⊙\;G,\;A_{dir}^{*}=A_{dir}\;⊙\;(1-G)\;. \end{array}$$ (13)

The symbol ⊙ denotes element-wise multiplication. When the gating coefficient *G* approaches 1, the network places more emphasis on the global contextual information provided by the channel self-attention. Conversely, when *G* approaches 0, the network relies more on directional attention, capturing anisotropic textures. Finally, the gated fusion of the two branches is added to the original input feature map via a residual connection, producing the final output *Y* of the DHCA module, as defined in formula (14). This lightweight gating mechanism achieves dual attention to direction and semantics, thereby effectively improving the discriminative ability of the features.


$$\begin{array}{c} Y=A\;⊙\;A_{channel}^{*}\;⊙\;A_{dir}^{*}\;+\;A_{0}\;. \end{array}$$ (14)

### 3.3. Two-stage spatial frequency enhancement module

**Fig 4:**
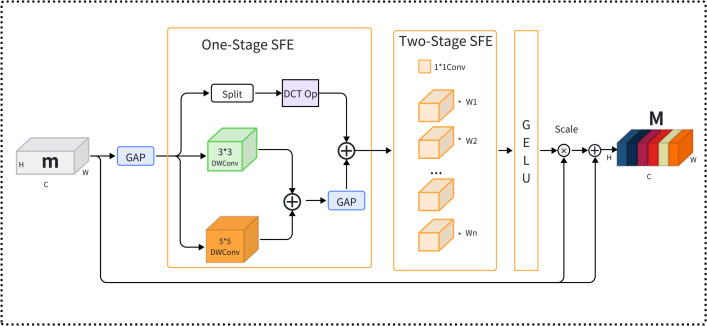
Two-stage spatial frequency enhancement module.

Under complex field conditions involving variable lighting, shading, and natural heterogeneity, lesions on rice leaves often exhibit a distinct set of characteristics. These include sparse high-frequency patterns, slight alterations in low-frequency shades, and uneven spatial arrangements. Conventional frameworks that rely solely on spatial convolutions or single-domain frequency attention struggle to capture this complementary information, which limits their performance in early disease detection. To address this challenge, we propose a two-stage spatial frequency enhancement module (TSSFE), as illustrated in Fig 4. This module aims to uniformly map the fine-grained texture amplitude and macro-shape contour of lesions to a discriminative feature space through spatial frequency collaborative modeling and dynamic weight adaptive aggregation.

In the first stage, the input feature map m$\in {\mathbb{R}}^{N\times C\times H\times W}$ is reshaped via adaptive pooling to obtain $m_{g}\in {\mathbb{R}}^{N\times C\times dct_{h}\times dct_{w}}$, which matches the required dimensions for the subsequent 2D DCT [40]. Inspired by the effective application of frequency domain analysis in visual recognition tasks by FcaNet [28], we propose to address the rice leaf disease detection problem by transforming the input features into the frequency domain and partitioning them into n channel-wise blocks $\left[m_{0},m_{1},\ldots ,m_{n-1}\right]$, each representing a distinct subset of channels, where each $m_{i}\in {\mathbb{R}}^{N\times C_{0}\times dct_{h}\times dct_{w}}$ with $C_{0}=\frac{C}{n}$. Subsequently, the data undergoes compression, and each group feature block is assigned a corresponding 2D DCT frequency component. This process [28] explicitly separates the high-frequency spot boundaries from the low-frequency tone variation features. The 2D DCT basis function $B_{h,w}^{u,v}$ is defined in formula (15). The resulting frequency-domain representation for the i-th block, denoted as $Freq_{i}$, is then computed according to formula 16).


$$\begin{array}{c} B_{h,w}^{u,v}=\cos\left(\frac{\pi h}{dct_{h}}\left(u+\frac{1}{2}\right)\right)\cos\left(\frac{\pi w}{dct_{w}}\left(v+\frac{1}{2}\right)\right). \end{array}$$ (15)

$$\begin{array}{c} Freq_{i}=2DDCT^{2}(m_{i})=\sum_{h=0}^{dct_{h}-1}\sum_{w=0}^{dct_{w}-1}m_{i}^{h,w}B_{h,w}^{u,v},i\in \{0,1,\ldots ,n-1\}. \end{array}$$ (16)

$h\in [0,{dct}_{h}],\;w\in [0,{dct}_{w}]$ are the pixel indices of the feature map in the height and width directions, respectively, and $u\in [0,{dct}_{h}],\;v\in [0,{dct}_{w}]$ represent the row and column oscillation frequency indices. The multi-spectral vector **m**_dct_ is obtained by concatenating each group of ${Freq}_{i}$.


$$\begin{array}{c} {𝐦}_{dct}\;=\;compress(m)\;=\;Concat([{Freq}^{0},\;{Freq}^{1},\;\ldots ,\;{Freq}^{n-1}]). \end{array}$$ (17)

We feed the input feature map $m_{g}\in {\mathbb{R}}^{N\times C\times dct_{h}\times dct_{w}}$ into two depth-wise separable convolutions with kernel sizes 3×3 and 5×5 to capture fine-grained and coarse-grained spatial structures. Global average pooling compresses the resulting feature maps to the channel descriptor $m_{sp}$, as shown in formula (18).


$$\begin{array}{c} m_{3}=DWConv_{3\times 3}(m_{g}),\;m_{5}=DWConv_{5\times 5}(m_{g}),\;m_{sp}=GAP(m_{3}+m_{5}). \end{array}$$ (18)

The subsequent integration of $m_{sp}$ and $m_{dct}$ is pivotal in achieving complementary feature superposition, thereby fusing texture frequency and spatial statistics into a space-frequency domain enhanced feature $m_{O-S}$, as shown in formula (19).


$$\begin{array}{c} m_{O-S}=m_{sp}+m_{dct}. \end{array}$$ (19)

The second stage enhances features by adaptively refining and integrating the space-frequency fusion features $m_{O-S}$. We design a set of *N* parallel 1×1 depthwise-separable convolution kernels *K*^(i)^. Each kernel is dedicated to capturing a latent frequency sub-band and therefore produces the corresponding intermediate feature *f*^(*i*)^, as shown in formula (20).


$$\begin{array}{c} f^{\left(i\right)}=K^{\left(i\right)}⊙m_{O-S},\;i=1,2,\ldots ,N. \end{array}$$ (20)

Each *f*(*i*) corresponds to the response of a specific frequency subband. To facilitate the selection of significant subbands, we incorporate a weight generator. After globally pooling $m_{O-S}$, a linear transformation and normalization (softmax) process derive the weight vector $w=[w_{1},w_{2},\ldots ,w_{N}]$. These weights multiply and sum the intermediate features. An adaptive aggregation of each frequency band by GELU activation, yielding two-stage space-frequency enhanced output $m_{T-S}$, detailed in formula (21).


$$\begin{array}{c} m_{T-S}=\sum_{i=1}^{N}w_{i}\;\text{GELU}(f^{\left(i\right)}). \end{array}$$ (21)

The output $m_{T-S}$ is passed through a multi-layer perceptron (MLP) to obtain attention weights. The resulting two-stage channel space-frequency weights are multiplied by the original feature map $m\in {\mathbb{R}}^{N\times C\times H\times W}$, and the result is output in a residual form as $M\in {\mathbb{R}}^{N\times C\times H\times W}$, according to the relation in formula (22):


$$\begin{array}{c} M=m+\alpha ⊙m. \end{array}$$ (22)

The symbol ⊙ indicates channel-wise multiplication. TSSFE is designed to jointly enhance high-frequency textures and low-frequency contours through a two-stage process of spatial-frequency collaborative modeling and dynamic frequency-band weighting. Concurrently, the residual structure essentially circumvents the suppression of original input features. The module is designed to be computationally efficient and can be readily integrated into existing network architectures. These features significantly enhance its sensitivity to false negatives in the early rice leaf spot detection. It achieves enhancement while maintaining minimal computational overhead.

### 3.4. Closed-loop tuning Bi-backbone network

**Fig 5:**
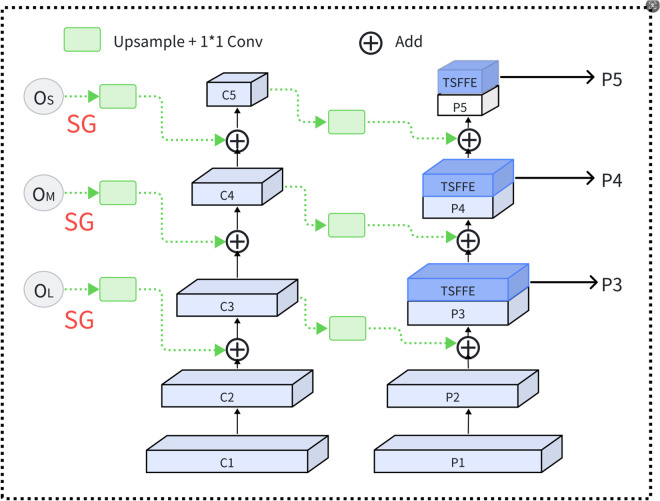
Closed-loop tuning Bi-backbone network.

In rice leaf spot monitoring, traditional single-path convolution methods often struggle to effectively mitigate error propagation. The existing techniques often fail to accurately capture fine details of small spots, convey high-level semantics, and maintain stability in intricate environments. To address these shortcomings, we design a Closed-loop Tuning Bi-Backbone Network (CLTB) architecture that actively fuses the auxiliary backbone’s high-resolution texture features with the primary backbone’s rich contextual information. As shown in Fig 5, the semantic information from the detection-level pyramid features is subsequently fed back to the shallow layer via closed-loop feedback, enabling sensitive detection of early lesions. Furthermore, it rectifies erroneous activations caused by ambient light and wind. Concurrently, the two-stage spatial frequency enhancement module integrated within the dual backbone ensures that the directional characteristics of striped lesions are amplified and sustained across the entire network. Compared with merely deepening the network, CLTB enhances the model’s generalization to leaf-spot images across seasons, cultivars, and shooting conditions. Dual backbones and spatial frequency interactions achieve this enhancement through their endogenous fusion.

The auxiliary backbone outputs $\left\{C_{1}^{\left(1\right)},\ldots ,C_{5}^{\left(1\right)}\right\}$ and the main backbone outputs $\left\{C_{1}^{\left(2\right)},\ldots ,C_{5}^{\left(2\right)}\right\}$ from the same input image $x\in {\mathbb{R}}^{H\times W\times 3}$, where the feature strides are {2,4,8,16,32} for stages 1–5, respectively. These features are subsequently aggregated by the neck to produce the multi-scale detection features $\left(O_{S},O_{M},O_{L}\right)$, as illustrated in formula (23).


$$\begin{array}{c} x_{l}=F_{l}(x_{l-1}). \end{array}$$ (23)

$F_{l}(\cdot )$ denotes the result that ensues after the *l* -th feature extraction. Despite its simplicity and efficiency, unidirectional propagation presents significant challenges in achieving a balance between low-level details and high-level context. In rice‐leaf spot detection, successive down-sampling operations often blur or even erase small lesions, making the problem particularly severe. Consequently, deep semantic analysis in later layers alone cannot fully recover the fine-grained edge information lost in early stages.

**Cross-stage Bi-backbone fusion (CLTB).** To address this issue, we propose a cross-stage bi-backbone fusion mechanism for CLTB, which deploys two backbones with identical structures in parallel: an auxiliary backbone $B^{\left(1\right)}=\{F_{l}^{\left(1\right)}{\}}_{l=1}^{5}$ and a main backbone $B^{\left(2\right)}=\{F_{l}^{\left(2\right)}{\}}_{l=1}^{5}$. Different from a literal cyclic computational graph, CLTB is implemented as an unrolled two-pass refinement with stop-gradient feedback. Specifically, we first run a standard forward pass through the main backbone and the neck to obtain a coarse set of neck outputs $\left(O_{S}^{\left(0\right)},O_{M}^{\left(0\right)},O_{L}^{\left(0\right)}\right)$. These coarse outputs serve solely as semantic guidance. Critically, they are detached using a stop-gradient operator. This ensures that no gradients flow from this feedback branch back to the main backbone during training. This design ensures that the overall training computation remains a directed acyclic graph, avoiding the optimization instability that may arise from cyclic backpropagation. The calculation process can be expressed as formula (24).


$$\begin{array}{c} ({\tilde{O}}_{S},{\tilde{O}}_{M},{\tilde{O}}_{L})=SG(O_{S}^{\left(0\right)},O_{M}^{\left(0\right)},O_{L}^{\left(0\right)}). \end{array}$$ (24)

**Semantic back-feeding in the auxiliary backbone.** For the auxiliary backbone *B*^(1)^, the detached neck features $\left\{{\tilde{O}}_{S},{\tilde{O}}_{M},{\tilde{O}}_{L}\right\}$ are fed to shallow stages (green path in Fig 5) to refine early representations. For each stage l∈{3,4,5}, we resize each feedback feature $O\in \{{\tilde{O}}_{S},{\tilde{O}}_{M},{\tilde{O}}_{L}\}$ to match the spatial resolution of the auxiliary feature map $x_{l-1}^{\left(1\right)}$ using nearest-neighbor interpolation $U_{O\to (l-1)}(\cdot )$, and align channels via a 1×1 convolution. These processed semantic features are summed to form a residual signal, which is added to $x_{l-1}^{\left(1\right)}$ to produce the refined feature. This update is defined in formula (25). The resulting semantic residuals are summed and added to obtain the updated feature, as shown in formula (25).


$$\begin{array}{c} {\bar{x}}_{l-1}^{\left(1\right)}=x_{l-1}^{\left(1\right)}+\sum_{O\in \left\{{\tilde{O}}_{S},\;{\tilde{O}}_{M},\;{\tilde{O}}_{L}\right\}}{Conv}_{1\times 1}\;\left(U_{O\to (l-1)}(O)\right),\;l\in \{3,4,5\}. \end{array}$$ (25)

The auxiliary stage then performs normal feed-forward extraction on the updated representation, as expressed in formula (26).


$$\begin{array}{c} x_{l}^{\left(1\right)}=F_{l}^{\left(1\right)}\;\left({\bar{x}}_{l-1}^{\left(1\right)}\right),\;l\in \{3,4,5\}. \end{array}$$ (26)

We denote the calibrated auxiliary features as $C_{l}≜x_{l}^{\left(1\right)}$. Semantic back-feeding helps suppress spurious activations caused by illumination variation, wind-induced motion, and background clutter, while restoring micro-lesion details attenuated by successive down-sampling.

**Aux→Main cross-stage injection and refinement.** CLTB injects the calibrated auxiliary features $\left\{C_{3},C_{4},C_{5}\right\}$ into the corresponding stages of the main backbone to couple high-resolution textures with multi-scale semantic context. Specifically, each $C_{l}$ is resampled to match the spatial resolution of $x_{l}^{\left(2\right)}$ and channel-aligned by a 1×1 convolution, then added to form the fused main backbone feature, as expressed in formula (27).


$$\begin{array}{c} {\bar{x}}_{l}^{\left(2\right)}=x_{l}^{\left(2\right)}+{Conv}_{1\times 1}\;\left(U_{C_{l}\to l}\;\left(C_{l}\right)\right),\;l\in \{3,4,5\}. \end{array}$$ (27)

We further apply the lightweight TSSFE to the fused main backbone features at P3−P5. Specifically, after injecting the calibrated auxiliary features into the main backbone to obtain ${\bar{x}}_{l}^{\left(2\right)}$ (Eq. (27)), we enhance them by ${\tilde{x}}_{l}^{\left(2\right)}=TSSFE({\bar{x}}_{l}^{\left(2\right)})$ for l∈{3,4,5}.

**Second-pass prediction.** After cross-stage fusion and TSSFE refinement, the second-pass neck aggregates the enhanced main backbone features and outputs three-scale detection features, as shown in formula (28).


$$\begin{array}{c} (O_{S}^{\left(1\right)},O_{M}^{\left(1\right)},O_{L}^{\left(1\right)})=𝒩({\tilde{x}}_{3}^{\left(2\right)},{\tilde{x}}_{4}^{\left(2\right)},{\tilde{x}}_{5}^{\left(2\right)}),\;\hat{Y}=ℋ(O_{S}^{\left(1\right)},O_{M}^{\left(1\right)},O_{L}^{\left(1\right)}). \end{array}$$ (28)

The training objective is computed only on the second-pass prediction, as shown in formula (29).


$$\begin{array}{c} ℒ=Loss(\hat{Y},Y^{*}). \end{array}$$ (29)

Here, 𝒩(·) denotes the neck module. ℋ(·) denotes the YOLO detection head that produces the final prediction $\hat{Y}$, including bounding-box regression and class confidence scores. $Y^{*}$ denotes the ground-truth annotations, and Loss(·) is the standard YOLO detection loss used for supervision. The superscript Pass-1 indicates the second-pass outputs in the unrolled refinement, whereas the Pass-0 outputs are used only for stop-gradient guidance.

**Algorithm 1: CLTB forward with stop-gradient feedback**



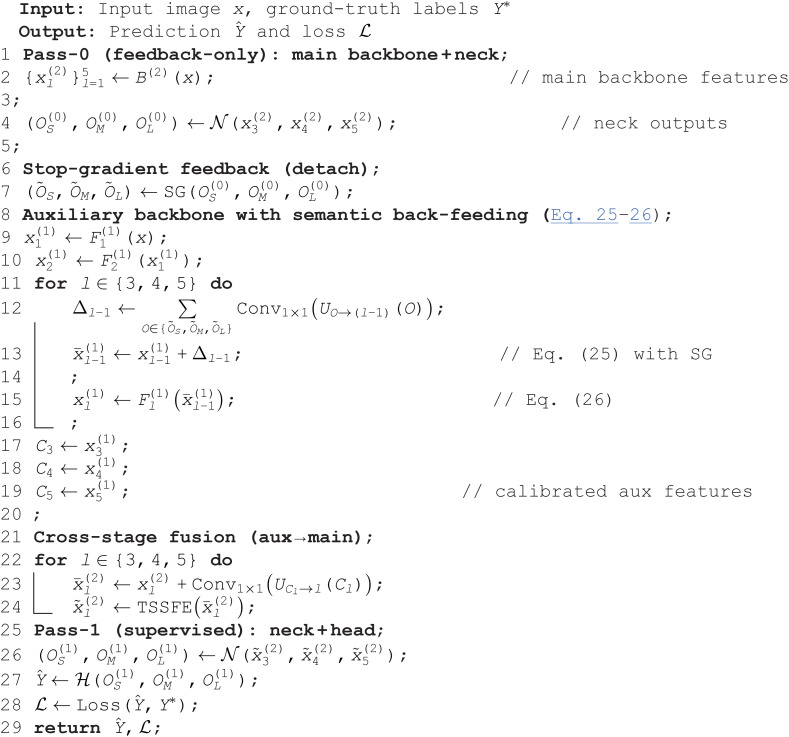



### 3.5. Scale-weighted fusion network

Under natural lighting and wind, rice leaf lesions are characterized by their small size, varied morphology, and low contrast. Consequently, accurate detection of these lesions is contingent upon high-resolution texture. However, YOLOv8 utilizes the PAN [20] architecture for feature fusion, which indiscriminately merges features of various scales and applies convolution. This uniform fusion facilitates the bidirectional flow of semantic and detailed information in general object detection. For fine-grained rice disease detection, this uniform weighting scheme is suboptimal. The PAN structure operates under the assumption that features of all scales hold equal importance. It lacks a mechanism to distinguish the priority between shallow-layer high-resolution textures and deep-layer semantic information. This results in insufficient recall rates for small disease spots in the early stages of the disease.

The Asymptotic Feature Pyramid Network (AFPN) [41] addresses this by employing a fully connected fusion scheme among features *P*_3_, *P*_4_, and *P*_5_. However, the direct fusion between features with extreme scale differences (e.g., high-resolution *P*_3_ and low-resolution *P*_5_) can be problematic due to their significant semantic and resolution gap. This interaction magnifies the semantic disparities between shallow and deep layers. The mismatch may enhance the influence of background highlights and leaf vein reflections, thereby increasing the false positive rate.

**Fig 6:**
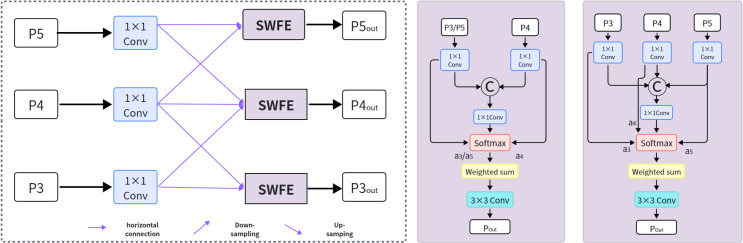
Scale-weighted fusion network.

To address the challenge of balancing uniform feature weighting with the capture of long-range, multi-scale dependencies, we propose the Scale-Weighted Fusion Network (SWFN), as illustrated in Fig 6. This network builds upon the architectural foundations of PAN [20] and AFPN [41]. SWFN takes the *P*_3_, *P*_4_, and *P*_5_ feature maps (80×80, 40×40, 20×20) from the backbone as input. 1×1 convolutions are applied to each to reduce their channel dimensions by half for efficient processing.


$$\begin{array}{c} P(3{)}_{\text{channel}}={\text{Conv}}_{1\times 1}(P3),\;P(4{)}_{\text{channel}}={\text{Conv}}_{1\times 1}(P4),\;P(5{)}_{\text{channel}}={\text{Conv}}_{1\times 1}(P5). \end{array}$$ (30)

After the adjustment of channels, the *P*(3)_channel_ channel undergoes downsampling, the *P*(5)_channel_ channel undergoes upsampling. We upsample and downsample the feature map *P*(4)_channel_ and send the resulting resolutions to the corresponding-scale SWFE blocks for feature fusion. Initially, a Conv1×1 is employed on $P_{k}$ to derive the weighted feature mapping $v_{k}$, which is defined as in formula (31).


$$\begin{array}{c} v_{k}={\text{Conv}}_{1\times 1}(P_{k}). \end{array}$$ (31)

When *k* = 3, 4, 5, the features are concatenated and a 1×1 convolution is applied to convert the concatenated features into unnormalized weights (logits), as shown in formula (32).


$$\begin{array}{c} \beta ={\text{Conv}}_{1\times 1}(\text{Concat}[v_{1},\ldots ,v_{K}]),\;\beta \in {\mathbb{R}}^{K\times H\times W}. \end{array}$$ (32)

For each spatial position (*i*, *j*), the logits vector (*i*, *j*) is normalized using the Softmax function, resulting in a pixel-level weight distribution $w_{k}(i,j)$ that satisfies $\sum_{k=1}^{K}w_{k}(i,j)=1$, as demonstrated in formula (33).


$$\begin{array}{c} w_{k}(i,j)=\frac{\exp(\beta_{k}(i,j))}{\sum_{m=1}^{K}\exp(\beta_{m}(i,j))}. \end{array}$$ (33)

Where *i* and *j* are used to denote spatial position coordinates, and $w_{k}(i,j)$ is defined as the fusion weight of the *k*-th branch at spatial position (*i*,*j*), which is obtained by applying Softmax normalization. $\beta_{k}(i,j)$ is defined as the logit value of the *k*-th branch at position (*i*,*j*). We apply the exponential function exp(·) to transform each branch logit into a non-negative score. For every spatial position (*i*,*j*), we normalise these scores by dividing by the sum of the *K* exponentials at each location, yielding the weight coefficients $w_{k}(i,j)$. We align and fuse the branch features by using $w_{k}(i,j)$ as pixel-wise weights. A final 3×3 convolution produces the output feature map *P*_out_ (see details in Eq. (34)).


$$\begin{array}{c} P_{\text{out}}={\text{Conv}}_{3\times 3}(\sum_{k=1}^{K}w_{k}(i,j)\;P_{k}(i,j)). \end{array}$$ (34)

The proposed SWFE module supports fusion with either two or three input feature maps. For each spatial location, SWFE predicts branch-wise fusion logits and normalizes them with a softmax across branches to obtain pixel-adaptive weights, which are used to compute a weighted sum of multi-scale features. This adaptive fusion enhances lesion-related cues at appropriate scales while keeping the neck lightweight.

## 4. Experiment

### 4.1. Experimental setup

**Datasets**. We employ three publicly available datasets for model training and evaluation: Rice Plant Diseases v9, Rice Plant Diseases v8, and the Rice Leaf Spot Disease dataset. All images are field-collected RGB color images, which were resized to 640×640 pixels and normalized during preprocessing to ensure data uniformity and model compatibility. The Rice Plant Diseases v9 and Rice Plant Diseases v8 datasets cover four categories of rice leaf diseases: Bacterial Leaf Blight, Grassy Stunt, Rice Blast, and Tungro. In contrast, the Rice Leaf Spot Disease Dataset encompasses eight classes: Bacterial Leaf Blight (BLB), Brown Spot, Healthy, Leaf Blast, Leaf Scald, Leaf Spot, Neck Blast, and Rice Hispa. For each dataset, we randomly partition the images into a training set and a test set using a 9:1 ratio. These categories, each with distinct visual characteristics, are crucial for computer vision tasks. The annotations provided are key for training models to classify different rice leaf diseases and assess their severity accurately. These categories, each with distinct visual characteristics, are crucial for computer vision tasks. The annotations provided are key for training models to classify different rice leaf diseases and assess their severity accurately.

**Experimental Settings**. The computational setup comprises an AMD EPYC 9754 CPU paired with an NVIDIA RTX 4090 GPU. The software stack runs on Ubuntu 20.04 LTS, with Python 3.10 and PyTorch 2.1.0 as the core framework, alongside OpenCV 4.11.0 for image preprocessing and augmentation. We employed the Adam optimizer to train the MCCA-YOLO model, with parameters $\beta_{1}$ and $\beta_{2}$ set to 0.9 and 0.999, respectively. The learning rate was initialized at 0.001, and a batch size of 16 was used. A weight decay of 0.0005 was applied for regularization to prevent overfitting. All input images were resized to 640×640 pixels. We employed early stopping with a patience of 50 epochs and set the maximum number of training epochs to 150.

### 4.2. Ablation experiments

We use the standard YOLOv8 model as our baseline and incrementally integrate the proposed modules to assess their individual contributions. We conduct a series of ablation studies to evaluate the proposed components, including Deformable Convolution (DCN), Channel Attention, Direction-Aware Attention (DA), Gating mechanisms, and their integration into the Deformable Hybrid Collaborative Attention (DHCA) module. Table 1 summarizes the ablation experiments for DHCA in the Neck on the Rice Plant Diseases Dataset v9, Rice Plant Diseases Dataset v8, and the Rice Leaf Spot Disease Dataset. Table 2 TSSFE is inserted at different feature-pyramid levels (P2–P4, P2–P5, P5, and P3–P5) for an ablation study. As shown in Table 3, we conducted comprehensive ablation studies on the proposed MCCA-YOLO framework using Rice Plant Diseases v9, Rice Plant Diseases v8, and Rice Leaf Spot Disease Dataset. These experiments systematically evaluated the contributions of our designed modules (DHCA, TSSFE, SWFN, and CLTB) by removing each component individually. In our ablation experiments, we report five metrics: precision, recall, mAP_@50_, mAP_@50:95_, and Params. Because mAP_@50_ is generally considered the most persuasive indicator for YOLO-based detectors, the subsequent analysis focuses on the relationship between each module’s mAP_@50_ and its parameter count.

**Table 1:** Ablation experiments for DHCA in the Neck across different datasets.

Data Set	Model	DCN	CCA	DA	GC	Precision	Recall	mAP_50_	mAP_50:95_	Params (M)
Rice plant v9	YOLOv8s (baseline)	×	×	×	×	0.828	0.844	0.860	0.588	11.1
	YOLOv8s+DCN	✓	×	×	×	0.851	0.840	0.873	0.626	**10.6**
	YOLOv8s+DCN + CCA	✓	✓	×	×	0.869	0.855	0.878	0.620	11.7
	YOLOv8s+DCN + CCA + DA	✓	✓	✓	×	0.873	0.858	0.882	0.625	11.9
	YOLOv8s+DHCA	✓	✓	✓	✓	**0.889**	**0.860**	**0.887**	**0.631**	12.0
Rice plant v8	YOLOv8s (baseline)	×	×	×	×	0.889	0.873	0.896	0.617	11.1
	YOLOv8s+DCN	✓	×	×	×	**0.903**	0.889	0.905	0.650	**10.6**
	YOLOv8s+DCN + CCA	✓	✓	×	×	0.888	0.881	0.908	0.653	11.7
	YOLOv8s+DCN + CCA + DA	✓	✓	✓	×	0.896	0.895	0.914	0.652	11.9
	YOLOv8s+DHCA	✓	✓	✓	✓	0.901	**0.900**	**0.918**	**0.659**	12.0
RLSD	YOLOv8s (baseline)	×	×	×	×	0.658	0.557	0.559	0.334	11.1
	YOLOv8s+DCN	✓	×	×	×	0.671	0.558	0.566	0.350	**10.6**
	YOLOv8s+DCN + CCA	✓	✓	×	×	0.630	0.567	0.571	0.359	11.7
	YOLOv8s+DCN + CCA + DA	✓	✓	✓	×	0.670	0.557	0.575	0.358	11.9
	YOLOv8s+DHCA	✓	✓	✓	✓	**0.684**	**0.573**	**0.578**	**0.366**	12.0

**Table 2:** Ablation experiments for TSSFE in the Backbone across different datasets.

Data Set	Model	Precision	Recall	mAP_50_	mAP_50:95_	Params (M)
Rice plant v9	P2–P4 with TSSFE	0.861	0.843	0.871	0.599	**11.5**
	P2–P5 with TSSFE	0.839	0.845	0.861	0.604	12.6
	P5 with TSSFE	0.841	0.852	0.861	0.600	12.2
	P3–P5 with TSSFE (ours)	**0.871**	**0.860**	**0.874**	**0.606**	12.6
Rice plant v8	P2–P4 with TSSFE	0.877	0.874	0.905	**0.641**	**11.5**
	P2–P5 with TSSFE	0.882	0.874	0.909	0.632	12.6
	P5 with TSSFE	0.864	0.889	0.906	0.638	12.2
	P3–P5 with TSSFE (ours)	**0.890**	**0.888**	**0.910**	0.634	12.6
RLSD	P2–P4 with TSSFE	0.640	0.541	0.565	0.325	**11.5**
	P2–P5 with TSSFE	0.628	0.555	0.569	0.334	12.6
	P5 with TSSFE	**0.683**	0.553	0.570	0.336	12.2
	P3–P5 with TSSFE (ours)	0.660	**0.565**	**0.574**	**0.341**	12.6

**Table 3:** Ablation experiments for MCCA-YOLO across different datasets.

Data Set	Model	TSSFE	CLTB	DHCA	SWFN	Precision	Recall	mAP_50_	mAP_50:95_	Params (M)
Rice plant v9	YOLOv8s (baseline)	×	×	×	×	0.828	0.844	0.860	0.588	11.1
	MambaYOLO (SOTA) [42]	×	×	×	×	0.848	0.857	0.875	0.606	21.8
	YOLOv8s+TSSFE	✓	×	×	×	0.871	0.860	0.874	0.606	12.6
	YOLOv8s+TSSFE+CLTB	✓	✓	×	×	0.884	0.844	0.884	0.626	30.5
	YOLOv8s+TSSFE+CLTB+DHCA	✓	✓	✓	×	0.874	0.876	0.893	**0.637**	31.0
	MCCA-YOLO (ours)	✓	✓	✓	✓	**0.888**	**0.880**	**0.904**	**0.637**	35.6
Rice plant v8	YOLOv8s (baseline)	×	×	×	×	0.889	0.873	0.896	0.617	11.1
	MambaYOLO (SOTA) [42]	×	×	×	×	0.891	0.866	0.908	0.641	21.8
	YOLOv8s+TSSFE	✓	×	×	×	0.890	0.888	0.910	0.634	12.6
	YOLOv8s+TSSFE+CLTB	✓	✓	×	×	0.886	0.899	0.915	0.661	30.5
	YOLOv8s+TSSFE+CLTB+DHCA	✓	✓	✓	×	0.899	0.888	0.920	0.658	31.0
	MCCA-YOLO (ours)	✓	✓	✓	✓	**0.915**	**0.900**	**0.922**	**0.662**	35.6
RLSD	YOLOv8s (baseline)	×	×	×	×	0.658	0.557	0.559	0.334	11.1
	MambaYOLO (SOTA) [42]	×	×	×	×	0.666	0.567	0.578	0.341	21.8
	YOLOv8s+TSSFE	✓	×	×	×	0.660	0.565	0.574	0.341	12.6
	YOLOv8s+TSSFE+CLTB	✓	✓	×	×	0.670	0.567	0.582	0.351	30.5
	YOLOv8s+TSSFE+CLTB+DHCA	✓	✓	✓	×	0.672	**0.576**	0.589	0.362	31.0
	MCCA-YOLO (ours)	✓	✓	✓	✓	**0.682**	0.574	**0.612**	**0.382**	35.6

Table 1 compares the baseline YOLOv8s with the modules introduced in Section 3.2. The baseline achieves mAP_@50_ of 86% with 11.1M parameters on the v9 dataset. The initial integration of Deformable Convolutional Networks [39] (DCN) into the YOLOv8s neck (YOLOv8s + DCN) demonstrates a notable improvement. This modification enables the network to adapt its sampling locations to capture the intricate contours of leaves better and differentiate them from surrounding healthy tissue. This yields mAP_@50_ improvements of +1.3% (v9), + 0.9% (v8), and +0.7% (RLSD) over the baseline, while reducing parameters by 0.5M. Further enhancement is observed with the fusion of DCN and the Channel Autocorrelation Attention (CCA) mechanism (YOLOv8s + DCN + CCA). CCA is designed to capture both local and global channel information, contributing to a slight improvement in mAP_@50_. This configuration improves mAP_@50_ by +1.8% (v9), + 1.2% (v8), and +1.2% (RLSD), at the cost of a 0.6M parameter increase relative to the DCN-only variant. The integration of Directional Attention (DA) in conjunction with DCN and CCA (YOLOv8s + DCN + CCA + DA) enhances the model’s performance by amplifying the network’s response to lesion-related textures. This configuration yields additional performance gains, with mAP_@50_ increasing by 2.2% on v9 datasets, 1.8% on v8 datasets, and 1.6% on RLSD datasets. The parameter count shows a modest increase to 11.9M. Finally, integrating the complete DHCA module yields the most significant gains, with mAP_@50_ improvements of +2.7% (v9), + 2.2% (v8), and +1.9% (RLSD) over the baseline, while adding only 0.9M parameters.

Table 2 analyzes the impact of incorporating the two-stage spatial frequency enhancement module (TSSFE) at various backbone levels on detection performance using the Rice Plant Diseases v9 dataset, Rice Plant Diseases v8 dataset, and Rice Leaf Spot Disease Dataset. The evaluation tested four integration approaches: (i) exclusively at the highest-level feature map P5; (ii) on the shallow feature maps P2˜P4; (iii) across all levels P2˜P5; (iv) on the mid-to-high levels P3˜P5 (the configuration ultimately selected). The designations P2, P3, P4, and P5, respectively, represent backbone feature maps with strides of 4, 8, 16, and 32. All additional training hyperparameters remained constant to isolate the effects of insertion position. When TSSFE is restricted to P5, precision increases by 1.3% compared to the YOLOv8s baseline on the v9 dataset; however, mAP_@50_ shows a minimal improvement of 0.1% due to the underutilization of fine-grained shallow features. Implementing TSSFE across P2˜P4 better utilizes these shallow features, resulting in an additional 1% improvement in mAP_@50_ relative to the P5 configuration on the v9 dataset. On the v8 and RLSD datasets, however, the P2˜P4 configuration performs comparably to or slightly below the P5-only setup. Furthermore, although extending implementation across all levels (P2˜P5) enhances information flow, it introduces feature interference, causing mAP_@50_ to decrease to 86.1% on the v9 dataset. Consequently, the P3˜P5 strategy was selected as it achieves an optimal balance between texture detail and semantic context while minimizing low-level noise. This approach delivers superior overall performance across all datasets. Compared to the baseline, mAP_@50_ improved by 1.4%, 1.4%, and 1.5% on the v9, v8, and RLSD datasets, respectively. Similarly, mAP_@50:95_ increased by 1.8%, 1.7%, and 0.7% on these datasets, requiring only 1.5M additional parameters. These consistent results indicate that the fusion of mid-to-high-level features proves particularly effective and robust for rice disease spot detection.

Table 3 demonstrates the individual and combined contributions of TSSFE, CLTB, DHCA, and SWFN components to the MCCA-YOLO detector. The baseline YOLOv8s achieves a mAP_@50_ of 86% on dataset V9, 89.6% on V8, and 55.9% on the RLSD dataset. Incorporating TSSFE in the backbone alone improves performance by 1.4% on the v9 and v8 datasets, and by 1.5% on the RLSD dataset. The combination of CLTB with TSSFE enhances mAP_@50_ by 2.4% on the v9 dataset, 1.9% on the v8 dataset, and 2.3% on the RLSD dataset, exceeding the performance gains of individual components and confirming their complementary nature. Integration of CLTB structure with TSSFE and DHCA yields mAP_@50_ improvements of 3.3% on the Rice Plant Diseases v9 dataset, 2.4% on the v8 dataset, and 3.0% on the RLSD dataset. The final MCCA-YOLO architecture incorporates four key components: the TSSFE module in the backbone, the DHCA module in the neck, and the concurrent CLTB and SWFN structures for enhanced feature aggregation. This comprehensive integration achieves optimal performance with mAP_@50_ improvements of 4.4% on the Rice Plant Diseases v9 dataset, 2.6% on the v8 dataset, and 5.3% on the RLSD dataset, as validated across all evaluation metrics. The proposed MCCA-YOLO demonstrates superior performance compared to the current state-of-the-art model, Mamba YOLO.

### 4.3. Comparison experiments

Comparative experiments were conducted by incorporating different modules (including SE [23], SEv2 [43], COT [27], ECA [25], CBAM [24], CA [26]) and the proposed TSSFE (F-block) and DHCA (S-block) into the YOLOv8s model to evaluate the impact of various attention modules on YOLOv8s detection performance. Table 4 shows the experimental outcomes for each variant, including Precision, Recall, mAP_@50_, mAP_@50:95_, and number of parameters (Param). The integration of attention modules generally enhances YOLOv8’s performance, demonstrating improved precision and recall compared to the baseline model without attention. Additionally, the mAP_@50_ and mAP_@50:95_ metrics have improved relative to the baseline model. However, the extent of these improvements varies among different attention types. The channel attention module SE improves mAP_@50_ by +0.8% (v9), + 1.2% (v8), and +0.6% (RLSD) with an addition of 0.06M parameters. SEv2 yields gains of +0.2% (v9), + 0.3% (v8), and +0.9% (RLSD) for 0.1M additional parameters. ECA, while adding only 0.03M parameters, enhances mAP@50 by +0.9% (v9), + 0.9% (v8), and +0.9% (RLSD). Modules incorporating spatial information achieve additional improvements. CBAM enhances mAP_@50_ by 0.7% on v9 and v8 datasets, and 0.4% on the RLSD dataset, with 0.3M additional parameters, while CA improves mAP_@50_ by 0.4% on v9, 1.0% on v8, and 0.8% on the RLSD dataset with a modest 0.03M parameter increase. Multi-scale attention through COT produces mAP_@50_ gains of 0.8% on v9, 0.6% on v8, and 1.2% on the RLSD dataset, though requiring 2.33M additional parameters, highlighting the trade-off between accuracy and model complexity when capturing extended dependencies. The proposed TSSFE and DHCA modules demonstrate superior performance characteristics. TSSFE module (F-block) increases YOLOv8’s mAP_@50_ by 1.4% on V9 and V8 datasets, and 1.5% on the RLSD dataset with 1.5M additional parameters, validating the F-block design’s effectiveness. The DHCA module (S-block) achieves optimal results among all variants, enhancing mAP_@50_ by 2.7% on V9, 2.2% on V8, and 1.9% on the RLSD dataset while adding only 0.9M parameters, maintaining model efficiency. The TSSFE and DHCA modules deliver substantial accuracy improvements with minimal computational overhead, demonstrating the advantages of this approach in both detection precision and computational efficiency.

**Table 4:** Comparison experiments for different attention blocks across different datasets.

Data Set	Model	Precision	Recall	mAP_50_	mAP_50:95_	Params (M)
Rice Plant v9	YOLOv8s+SE [23]	0.857	0.852	0.868	0.594	11.16
	YOLOv8s+SEv2 [43]	0.849	0.848	0.862	0.597	11.20
	YOLOv8s+COT [27]	0.833	0.848	0.868	0.598	13.43
	YOLOv8s+ECA [25]	0.829	0.858	0.869	0.592	11.13
	YOLOv8s+CBAM [24]	0.838	0.848	0.867	0.580	11.40
	YOLOv8s+CA [26]	0.870	0.831	0.864	0.602	11.16
	YOLOv8s+TSSFE (ours)	**0.871**	**0.860**	**0.874**	**0.606**	12.60
	YOLOv8s+DHCA (ours)	**0.889**	**0.860**	**0.887**	**0.631**	12.00
Rice Plant v8	YOLOv8s+SE [23]	0.873	0.879	0.908	0.621	11.16
	YOLOv8s+SEv2 [43]	0.891	0.842	0.899	0.622	11.20
	YOLOv8s+COT [27]	0.877	0.869	0.902	0.626	13.43
	YOLOv8s+ECA [25]	0.891	0.865	0.905	0.624	11.13
	YOLOv8s+CBAM [24]	0.890	0.878	0.903	0.621	11.40
	YOLOv8s+CA [26]	0.886	0.861	0.906	0.628	11.16
	YOLOv8s+TSSFE (ours)	0.890	**0.888**	**0.910**	**0.634**	12.60
	YOLOv8s+DHCA (ours)	**0.901**	**0.890**	**0.918**	**0.659**	12.00
RLSD	YOLOv8s+SE [23]	0.625	0.562	0.565	0.327	11.16
	YOLOv8s+SEv2 [43]	0.619	0.553	0.568	0.334	11.20
	YOLOv8s+COT [27]	0.663	0.523	0.571	0.328	13.43
	YOLOv8s+ECA [25]	0.628	0.565	0.568	0.324	11.13
	YOLOv8s+CBAM [24]	0.660	0.538	0.563	0.330	11.40
	YOLOv8s+CA [26]	0.619	0.572	0.567	0.335	11.16
	YOLOv8s+TSSFE (ours)	0.660	**0.565**	**0.574**	**0.341**	12.60
	YOLOv8s+DHCA (ours)	**0.684**	**0.573**	**0.578**	**0.366**	12.00

**Fig 7:**
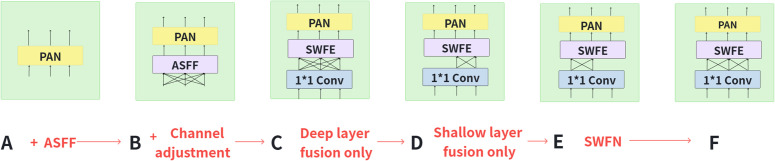
Evolution and comparison analysis of the SWFN architecture.

Table 5 demonstrates the impact of cross-scale feature fusion on YOLOv8 for rice disease detection. The Baseline (Model A, Fig 7(A)) employs the original PANet neck. Implementing a complete AFPN [41] (Model B, Fig 7(B)) facilitates comprehensive P3 P5 information exchange. While it improves mAP_@50_ by 1.4% on v9 and by 1.1% on the RLSD dataset, it leads to a 1.8% drop on v8 and introduces an additional 6.5M parameters. The potential channel mismatch and gradient conflict issues were addressed through precise convolution resizing and feature channel partitioning. Decreasing channel width before fusion (Model C, Fig 7(C)) reduces parameters by 4.7M compared to Model B. Although mAP_@50_ decreases 0.3% on the v9 dataset and 0.5% on the RLSD dataset, the method yields a notable gain of 3.2% on the v8 dataset. Considering both the substantial parameter reduction and the overall performance profile, Model C provides a well-balanced accuracy–complexity trade-off. When restricting fusion to deep layers P4 and P5 (Model D, Fig 7(D)), the mAP_@50_ increases marginally by 0.2% on v9, 0.7% on v8, and 1.9% on the RLSD dataset. Prioritizing shallow cues through P3 and P4 fusion (Model E, Fig 7(E)) provides more uniform improvements, increasing mAP_@50_ by 1.0% on v9, 1.3% on v8, and significantly by 2.8% on the RLSD dataset, while notably improving recall. The enhanced recall highlights the significance of reinforced low-level features in small-object detection. Our proposed SWFN (Model F, Fig 7(F)) selectively fuses context into P4. It achieves mAP@50 improvements of +1.4% (v9), + 1.4% (v8), and +3.8% (RLSD) over the baseline, adding only 1.3M parameters. Model F also achieves high recall (84.4% v9, 88.2% v8, 57.1% RLSD) and precision (86.6% v9, 89.9% v8, 68.8% RLSD). This comprehensive performance indicates that strategic cross-scale fusion can substantially reduce detection failures while maintaining effective model scale and false positive control.

**Table 5:** Comparison experiment for SWFN in the Neck across different datasets.

Data Set	Model	Precision	Recall	mAP_50_	mAP_50:95_	Params (M)
Rice Plant v9	A	0.828	0.844	0.860	0.588	11.10
	B	0.831	0.844	**0.874**	0.597	17.60
	C	0.858	0.842	0.871	0.603	12.90
	D	0.848	0.841	0.862	0.597	12.10
	E	0.849	**0.851**	0.870	0.598	11.40
	F (ours)	**0.866**	0.844	**0.874**	**0.609**	12.40
Rice Plant v8	A	0.889	0.873	0.896	0.617	11.10
	B	0.872	0.847	0.878	0.619	17.60
	C	0.897	0.868	**0.910**	0.635	12.90
	D	0.889	0.875	0.903	0.630	12.10
	E	0.891	0.873	0.909	0.634	11.40
	F (ours)	**0.899**	**0.882**	**0.910**	**0.642**	12.40
RLSD	A	0.658	0.557	0.559	0.334	11.10
	B	0.668	0.559	0.570	0.330	17.60
	C	0.631	0.552	0.564	0.329	12.90
	D	0.668	0.568	0.578	0.350	12.10
	E	0.676	0.558	0.587	0.343	11.40
	F (ours)	**0.688**	**0.571**	**0.597**	**0.361**	12.40

Table 6 compares the proposed MCCA-YOLO with several state-of-the-art detectors—Faster R-CNN [44], YOLOv5s, YOLOv8, BGF-YOLO [45], Mamba YOLO [42], and RT-DETR [46]—on the rice disease datasets. The evaluation metrics include precision, recall, mAP_@50_, mAP_@50:95_, and parameters. On the v9 dataset, MCCA-YOLO achieves the highest mAP_@50_ (0.904) and mAP_@50:95_ (0.637) among all compared models. These results surpass YOLOv8 and Mamba YOLO. The consistently high mAP values of MCCA-YOLO demonstrate its robust capability to identify and localize rice plant diseases in challenging environments with high accuracy. The model’s precision of 0.888 and recall of 0.880 indicate an effective balance between minimizing false positives and maximizing true positives, enabling reliable detection across various disease categories.

**Table 6:** Comparison experiment of different models across different datasets.

Data Set	Model	Precision	Recall	mAP_50_	mAP_50:95_	Params (M)
Rice plant v9	Faster R-CNN [44]	–	–	0.623	0.379	6.7
	YOLOv5s	0.843	0.827	0.840	0.532	7.0
	YOLOv8s	0.828	0.844	0.860	0.588	11.1
	BGF-YOLO (MICCAI 2024) [45]	0.814	0.804	0.843	0.550	3.4
	RT-DETR (CVPR 2024) [46]	0.832	0.834	0.851	0.564	32.8
	MambaYOLO (AAAI 2025) [42]	0.848	0.857	0.875	0.606	21.8
	MCCA-YOLO (ours)	**0.888**	**0.880**	**0.904**	**0.637**	35.6
Rice plant v8	Faster R-CNN [44]	–	–	0.783	0.506	6.7
	YOLOv5s	0.868	0.856	0.882	0.533	7.0
	YOLOv8s	0.889	0.873	0.896	0.617	11.1
	BGF-YOLO (MICCAI 2024) [45]	0.861	0.859	0.891	0.583	3.4
	RT-DETR (CVPR 2024) [46]	0.896	0.878	0.905	0.603	32.8
	MambaYOLO (AAAI 2025) [42]	0.891	0.866	0.908	0.641	21.8
	MCCA-YOLO (ours)	**0.915**	**0.900**	**0.922**	**0.662**	35.6
RLSD	Faster R-CNN [44]	–	–	0.365	0.161	6.7
	YOLOv5s	0.629	0.543	0.540	0.299	7.0
	YOLOv8s	0.658	0.557	0.559	0.334	11.1
	BGF-YOLO (MICCAI 2024) [45]	0.595	0.495	0.509	0.278	3.4
	RT-DETR (CVPR 2024) [46]	0.680	0.570	0.582	0.353	32.8
	MambaYOLO (AAAI 2025) [42]	0.666	0.567	0.578	0.341	21.8
	MCCA-YOLO (ours)	**0.682**	**0.574**	**0.612**	**0.382**	35.6

MCCA-YOLO maintains its superior performance on the v8 dataset, achieving a precision of 0.915 and a recall of 0.900. The model surpasses other state-of-the-art detectors in both mAP_@50_ (0.922) and mAP_@50:95_ (0.662), further validating its effectiveness in handling diverse and complex plant disease patterns. This performance demonstrates MCCA-YOLO’s accuracy and adaptability to varying real-world agricultural conditions.

On the more challenging RLSD dataset, MCCA-YOLO achieves a precision of 0.682 and a recall of 0.574. The model outperforms other advanced detectors, including RT DETR and Mamba YOLO, with a mAP_@50_ of 0.612 and mAP_@50:95_ of 0.382. These results underscore the model’s capability to handle the complex background and small lesion features characteristic of the RLSD dataset, significantly outperforming the YOLOv8s baseline and other recent architectures.

MCCA-YOLO has 35.6M parameters. The consistent and substantial accuracy gains it delivers across all three datasets demonstrate a favorable accuracy-complexity trade-off compared to other high-performance models. The model’s ability to deliver enhanced detection capabilities while maintaining competitive parameter efficiency demonstrates an optimal balance between computational requirements and accuracy.

**Fig 8:**
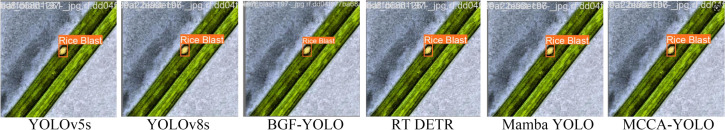
Comparison of the visualization results between our model and other models on dataset V9.

**Fig 9:**
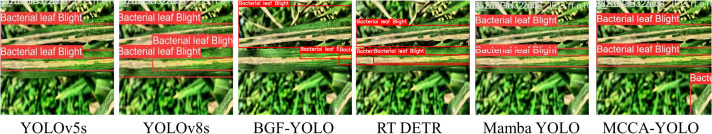
Comparison of the visualization results between our model and other models on dataset V8.

**Fig 10:**
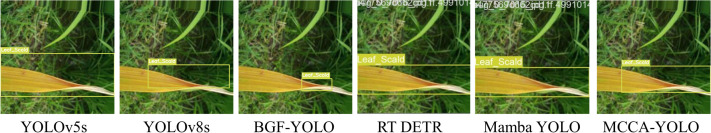
Comparison of the visualization results between our model and other models on dataset RLSD.

To further evaluate the robustness and cross-dataset generalization of MCCA-YOLO, we conduct a qualitative comparison on three datasets: Rice Plant Diseases v9, Rice Plant Diseases v8, and RLSD. Figs 8, 9, and 10 present representative cases with typical challenges, including tiny lesions with low contrast (Rice Blast), elongated disease regions in cluttered backgrounds (Bacterial Leaf Blight), and large-area symptoms with ambiguous boundaries (Leaf Scald). Detection results from MCCA-YOLO are compared with those from several state-of-the-art detectors (YOLOv5s, YOLOv8s, BGF-YOLO, RT-DETR, Mamba YOLO) under identical conditions. As shown in Fig 8, the Rice Blast lesion is small and visually similar to surrounding textures, which often leads to imprecise localization or missed detections for baseline models. For the elongated Bacterial Leaf Blight pattern in Fig 9, some detectors produce fragmented or over-extended bounding boxes that do not align well with the true lesion extent. In contrast, MCCA-YOLO yields more consistent localization with tighter bounding boxes around the actual diseased areas. For RLSD in Fig 10, where symptoms cover a relatively large region and the boundary is weak, several methods either under-localize or over-localize, whereas MCCA-YOLO better balances completeness and precision. Overall, these visual results indicate that MCCA-YOLO is more robust to variations in scale, appearance, and background complexity, demonstrating strong generalization across different agricultural datasets.

**Fig 11:**
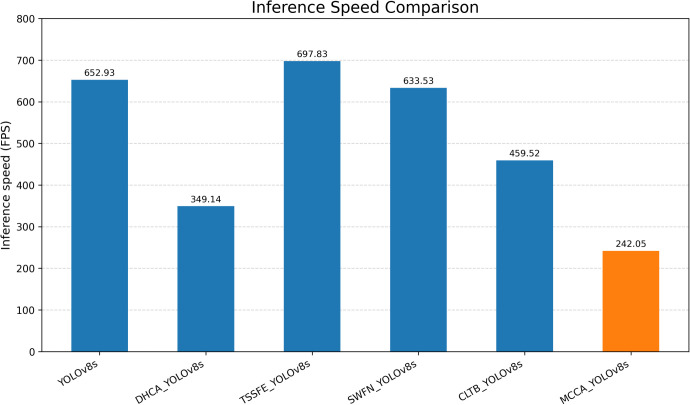
Comparison experiment on inference speed across different models.

Fig 11 shows the inference speeds of the six evaluated models on the RTX 4090 device. The baseline YOLOv8 achieves 652.93 FPS. After integrating the proposed modules, MCCA-YOLO’s inference speed is 242.05 FPS. Even with the reduced speed, our MCCA-YOLO is still capable of real-time inference for practical applications. The widely accepted threshold for real-time detection is approximately 30 FPS, as this rate aligns with standard video frame rates and ensures smooth visual perception for human observers [47]. These studies show that the inference speed for our model is practical. MCCA-YOLO is designed to balance detection accuracy with computational efficiency. It is crucial to recognize the inherent trade-off between model accuracy and inference speed. While advancements in computational hardware can significantly accelerate inference, gains in accuracy are fundamentally driven by the sophistication of the model’s architecture, which often entails greater computational complexity.

### 4.4. Real-world model deployment

To validate the practical applicability of MCCA-YOLO, we deploy and evaluate it on the Huawei Atlas 200I DK edge AI platform. The edge device used in this experiment is the Atlas 200I DK hardware platform.

To test the effectiveness of the MCCA-YOLO model it is deploved on the Atlas 200I DK edge device platform in this paper. The Atlas 200I DK is equipped with the Ascend 310P AI processor, which offers an AI computing power of 20 TOPS and 10 TFLOPS. The Central Processing Unit (CPU) configuration features the Advanced RISC Machine (ARM) Cortex-A76, along with 8GB of LPDDR4 memory. Based on the proposed model, we designed an inference algorithm for the local deployment model of MCCA-YOLO on the Atlas 200I DK edge device. We converted the trained .pt model file in PyTorch into an .om model file that can be supported for inference by the Ascend AI processor of the edge device. In our testing pipeline, a camera captures images of rice leaves, which are then processed by the deployed MCCA-YOLO model. Detection results are rendered in real-time on the device’s screen. This section introduces the experimental process of implementing rice leaf pathology detection by deploying the proposed model on the edge device to verify the effectiveness of the MCCA-YOLO method in the real world.

**Fig 12:**
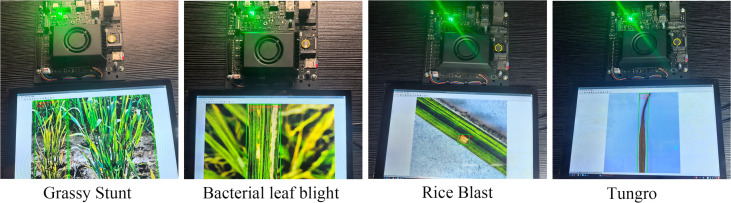
Local deployment of the model and visualization of detection results.

Fig 12 shows real-time detection visualizations for four major rice leaf diseases on the Atlas 200I DK. The experimental results show that the MCCA-YOLO model can continuously locate and identify the symptoms of rice leaf diseases. The model generates precise bounding boxes around the affected areas, accompanied by confidence scores (Rice Blast 0.87, Grassy Stunt 0.86, Bacterial Leaf Blight 0.78, Tungro 0.73). These high-confidence predictions demonstrate the model’s robustness and suitability for deployment on resource-constrained edge hardware. The model can effectively identify the characteristic yellow lesions of Bacterial Leaf Blight. It can also successfully distinguish plants infected with Grassy Stunt disease among dense foliage. In terms of Rice Blast detection, the model can accurately identify the typical spindle-shaped lesions. This deployment experiment validates the practical viability of MCCA-YOLO for real-time, in-field rice disease detection using edge computing devices.

## 5. Conclusion

With the wide application of computer vision technology in fields such as agriculture and ecological monitoring, fast and accurate automatic target detection in complex natural scenes has become a key challenge. The performance of traditional detection methods declines in complex situations such as drastic changes in lighting and large differences in target scales. Therefore, it is essential to develop new target detection models. To address this, we propose MCCA-YOLO, a novel YOLO-based architecture for automated rice leaf disease detection. It incorporates four key innovations to significantly enhance detection performance.

Since a single backbone network faces difficulties in handling the error propagation problem in deep neural networks, we propose a Closed-Loop Tuning Bi-Backbone network (CLTB). It reduces error propagation through a closed-loop feedback mechanism, effectively enhances the model’s multi-scale feature extraction ability, and improves the feature representation ability. To reduce the high-frequency noise generated by soil particles on rice leaves and the interference of water droplet reflections, which leads to a decline in system performance, we propose a Two-Stage Spatial Frequency Feature Enhancement (TSSFE) model in the main backbone network. This model enhances texture and edge information in the frequency domain and effectively improves the recognition ability for small targets and complex backgrounds. To enhance the complementarity of rice leaf feature information and strengthen the responsiveness of multi-scale features to rice leaf lesions, we designed a Scale Weight Fusion Network (SWFN). It dynamically adjusts the fusion weights of multi-scale features and improves the system’s robustness in complex scenarios. To achieve precise focusing on the key lesion areas of rice leaves, we propose a Deformable Hybrid Collaborative Attention (DHCA) mechanism during neck feature fusion. This mechanism combines direction-aware attention and channel self-attention to adapt to leaf deformation and texture directions. Extensive experiments on multiple rice disease datasets show that MCCA-YOLO achieves state-of-the-art detection accuracy and robustly handles the complexities of in-field imagery.

Future work will explore several promising directions to build upon this research. Currently, data augmentation techniques mainly rely on visual data and existing domain expertise. In the future, parameterized generation methods can be adopted, which will facilitate more accurate and diverse augmentation of the lesion areas, thereby enhancing the model’s generalization ability in rare cases and challenging scenarios. In addition, although this study focuses on RGB imaging, the fusion of thermal infrared and hyperspectral imaging remains unexplored. Cross-spectral feature fusion has great potential in revealing pathophysiological mechanisms and early potential changes. By exploring cross-spectral feature fusion networks and researching fusion strategies at different levels (data level, feature level, and decision level), the fusion effect of different information sources can be further enhanced. Finally, although the model performs excellently on the existing datasets, there is still room for improvement in optimizing its lightweight performance on edge devices. Future research aims to utilize methods such as neural architecture search (NAS) and knowledge distillation to enhance inference speed while maintaining accuracy. With the increasing popularity of multispectral agricultural imaging data, it will be crucial to verify the scalability and robustness of the model.

## Supporting information

S1 Table Arrangement of the rice plant diseases v9 dataset. (PDF)

S2 Table Arrangement of the rice plant diseases v8 dataset. (PDF)

S3 Table Arrangement of the rice plant diseases RLSD dataset. (PDF)

S4 Table Hardware configuration. (PDF)

S5 Table Software configuration. (PDF)

S6 Table Key hyperparameter settings. (PDF)
