# Supplementary material for: Multi-scale closed-loop tuning via spatial frequency collaborative sensitivity for rice leaf disease detection
Source: PLoS One. 2026 Jun 18;21(6):e0351727. doi: 10.1371/journal.pone.0351727 (PMC13278584; doi:10.1371/journal.pone.0351727)
Supplement: S1 Table — (PDF) [file pone.0351727.s001.pdf]

S1 Table. Arrangement of the rice plant diseases v9 dataset.

Class	Bacterial Leaf Blight	Grassy Stunt	Rice Blast	Tungro	Total
Train	1500	1341	2501	2073	7415
Validation	155	123	199	226	703
Total	1655	1464	2700	2299	8118
