# Supplementary material for: Multi-scale closed-loop tuning via spatial frequency collaborative sensitivity for rice leaf disease detection
Source: PLoS One. 2026 Jun 18;21(6):e0351727. doi: 10.1371/journal.pone.0351727 (PMC13278584; doi:10.1371/journal.pone.0351727)
Supplement: S2 Table — (PDF) [file pone.0351727.s002.pdf]

S2 Table. Arrangement of the rice plant diseases v8 dataset.

Class	Bacterial Leaf Blight	Grassy Stunt	Rice Blast	Tungro	Total
Train	1374	1716	2502	2352	7944
Validation	145	168	199	236	748
Total	1519	1884	2701	2588	8692
