# Supplementary material for: Multi-scale closed-loop tuning via spatial frequency collaborative sensitivity for rice leaf disease detection
Source: PLoS One. 2026 Jun 18;21(6):e0351727. doi: 10.1371/journal.pone.0351727 (PMC13278584; doi:10.1371/journal.pone.0351727)
Supplement: S3 Table — (PDF) [file pone.0351727.s003.pdf]

S3 Table. Arrangement of the rice leaf spot disease RLSD dataset.

Class	BLB	Brown Spot	Healthy	Leaf Blast	Leaf Scald	Leaf Spot	Neck Blast	Rice Hispa	Total
Train	531	1148	662	634	452	572	770	697	5466
Validation	58	128	76	63	60	66	100	105	656
Total	589	1276	738	697	512	638	870	802	6122
