# Supplementary material for: Multi-scale closed-loop tuning via spatial frequency collaborative sensitivity for rice leaf disease detection
Source: PLoS One. 2026 Jun 18;21(6):e0351727. doi: 10.1371/journal.pone.0351727 (PMC13278584; doi:10.1371/journal.pone.0351727)
Supplement: S4 Table — (PDF) [file pone.0351727.s004.pdf]

S4 Table. Hardware configuration.

Hardware	Configuration
CPU	AMD EPYC 9754 128-Core Processor
GPU	NVIDIA RTX 4090 (24 GB)
RAM	60 GB
Storage	120 GB SSD
