# Supplementary material for: Multi-scale closed-loop tuning via spatial frequency collaborative sensitivity for rice leaf disease detection
Source: PLoS One. 2026 Jun 18;21(6):e0351727. doi: 10.1371/journal.pone.0351727 (PMC13278584; doi:10.1371/journal.pone.0351727)
Supplement: S5 Table — (PDF) [file pone.0351727.s005.pdf]

S5 Table. Software configuration.

Software	Configuration
Operating system	Ubuntu 20.04 LTS
Python	3.10
PyTorch (GPU)	2.1.0
OpenCV	4.11.0
