# Supplementary material for: Multi-scale closed-loop tuning via spatial frequency collaborative sensitivity for rice leaf disease detection
Source: PLoS One. 2026 Jun 18;21(6):e0351727. doi: 10.1371/journal.pone.0351727 (PMC13278584; doi:10.1371/journal.pone.0351727)
Supplement: S6 Table — (PDF) [file pone.0351727.s006.pdf]

S6 Table. Key hyperparameter settings.

Parameter	Value
Model	MCCA-YOLO
Epochs	150
Batch size	16
Optimizer	Adam ($\beta_1 = 0.9$, $\beta_2 = 0.999$)
Learning rate	0.001
Weight decay	0.0005
Image size	640×640 px
Early stopping patience	50 epochs
