# Supplementary material for: Multi-scale closed-loop tuning via spatial frequency collaborative sensitivity for rice leaf disease detection
Source: PLoS One. 2026 Jun 18;21(6):e0351727. doi: 10.1371/journal.pone.0351727 (PMC13278584; doi:10.1371/journal.pone.0351727)
Supplement: Attachment — Submitted filename: PONE-D-25-58819-review.docx [file pone.0351727.s007.docx]

**Summary:**

This paper presents a well-structured and logically coherent study addressing the critical need for early rice leaf disease detection. The proposed MCCA-YOLO model, with its closed-loop tuning architecture and adaptive feature fusion strategies, effectively targets the challenges of subtle early lesion identification and complex lesion morphology. Its strong experimental performance underscores its value, offering meaningful insights for agricultural disease diagnosis and contributing a practical reference to the field.

However, three areas require improvement:

1. **Image Layout Misalignment:** Several images in the paper exhibit misaligned layouts, which compromises visual clarity and may distract readers from key findings.

**2. Insufficient Comparative Visuals:** While quantitative results are compelling, additional images illustrating detection effects across diverse datasets would better demonstrate the model’s robustness and real-world applicability.

**3. Excessive Length:** The paper is overly lengthy; non-innovative details (e.g., routine method descriptions or standard experimental setup steps) should be relocated to supplementary materials to streamline the main text and enhance focus.

Overall, this work has significant merits, and addressing these minor flaws will further elevate its academic impact and readability.
