# Supplementary material for: Multi-scale closed-loop tuning via spatial frequency collaborative sensitivity for rice leaf disease detection
Source: PLoS One. 2026 Jun 18;21(6):e0351727. doi: 10.1371/journal.pone.0351727 (PMC13278584; doi:10.1371/journal.pone.0351727)
Supplement: Attachment — Submitted filename: Response to Reviewers.docx [file pone.0351727.s009.docx]

Revision Report:

Article title: Multi-scale closed-loop tuning via spatial-frequency collaborative sensitivity for rice leaf disease detection

Dear reviewer,

We would like to express our sincere gratitude to the reviewer for the encouraging comments and the recognition of our work's value in agricultural disease diagnosis. We have carefully considered the three suggestions regarding image layout, comparative visuals, and manuscript length. We have revised the manuscript accordingly. The revised texts are marked in red in the manuscript. Please find our point-by-point responses below.

# Reviewer 1:

1) Comment 1: Image Layout Misalignment: Several images in the paper exhibit misaligned layouts, which compromises visual clarity and may distract readers from key findings.

**Response**: We apologize for the oversight regarding the image layout in the original submission. Thank you for pointing this error. We have professionally reformatted all figures in the revised manuscript to ensure proper alignment, consistent margins, and high visual clarity. Specifically, we have adjusted Figure to correct the layout issues. We have also checked the final PDF to ensure that text and figures are correctly positioned and do not overlap.

The PLOS ONE system requires figures to be uploaded as individual files, and they appear as such in the auto-generated review PDF. They are typically compiled and placed at the end of the document by default rather than displayed next to their captions in the main text. As a result, captions may appear in the manuscript body while the corresponding images are collected at the bottom, and the hyperlinks may direct to the caption location instead of an in-text embedded figure. To help readers navigate the manuscript more easily, we have checked that all figures are clearly and consistently cited in the text (e.g., “Fig X”) and that each caption matches the correct uploaded figure file.

2) Comment 2: Insufficient Comparative Visuals: While quantitative results are compelling, additional images illustrating detection effects across diverse datasets would better demonstrate the model’s robustness and real-world applicability.

**Response:** We sincerely appreciate the reviewer’s valuable suggestion, we agree that visual comparisons are crucial for demonstrating the model's robustness. To address this, we have added three new figures (Figure [8][9][10]) in the revised manuscript. This figure presents a qualitative comparison between our MCCA-YOLO and the baseline model (YOLOv8s) as well as other state-of-the-art models (e.g., Mamba YOLO) on challenging samples from both the Rice Plant Diseases v8 dataset ,Rice Plant Diseases v9 dataset,and RLSD dataset. These samples include scenarios with complex backgrounds, small early-stage lesions, and variable lighting conditions. As shown in figure [8][9][10], our model demonstrates superior localization accuracy and a lower false-negative rate compared to other methods, visually confirming the quantitative results presented in Table 19, Table 20 and Table 21, as below:

*The following paragraph has been added to section 4.3 (on page 25).*

*“To further evaluate the robustness and cross-dataset generalization of MCCA-YOLO, we conduct a qualitative comparison on three datasets: Rice Plant Diseases v9, Rice Plant Diseases v8, and RLSD. Fig 8, Fig 9, and Fig 10 present representative cases with typical challenges, including tiny lesions with low contrast (Rice Blast), elongated disease regions in cluttered backgrounds (Bacterial Leaf Blight), and large-area symptoms with ambiguous boundaries (Leaf Scald). We compare MCCA-YOLO with several competitive detectors (YOLOv5s, YOLOv8s, BGF-YOLO, RT-DETR, and Mamba YOLO) under the same visualization settings. As shown in Fig 8, the rice blast lesion is small and visually similar to surrounding textures, which often leads to imprecise localization or missed detections for baseline models. In Fig 9, bacterial leaf blight exhibits long stripe-like patterns across leaves, competing methods tend to produce fragmented predictions, duplicated boxes, or over-extended boxes covering irrelevant regions. In contrast, MCCA-YOLO yields more consistent localization with tighter bounding boxes around the actual diseased areas. For RLSD in Fig 10, where symptoms cover a relatively large region and the boundary is weak, several methods either under-localize or over-localize, whereas MCCA-YOLO better balances completeness and precision. Overall, these visual results indicate that MCCA-YOLO is more robust to variations in scale, appearance, and background complexity, demonstrating strong generalization across different agricultural datasets.”*

*Fig 8. Visualization of detection results for Dataset V9.*

*Fig 9. Visualization of detection results for Dataset V8.*

*Fig 10. Visualization of detection results for the RLSD Dataset.*

3) Comment 3: Excessive Length: The paper is overly lengthy; non-innovative details (e.g., routine method descriptions or standard experimental setup steps) should be relocated to supplementary materials to streamline the main text and enhance focus.

**Response:** We greatly appreciate the reviewer’s suggestion to improve readability. We strictly followed this suggestion to streamline the manuscript. We have made the following major revisions:

1. Removed Routine Descriptions: We deleted the detailed description of the standard YOLOv8 detection head (original submission Section 3.6) and condensed the background descriptions of the datasets (Section 4.1). To enhance the generalization capability of the model, we have additionally incorporated an RLSD dataset for rice leaf pathology analysis. The updates have been highlighted in red color in the manuscript, as below:

*The following paragraph has been updated to section 4.1 (on page 17).*

*“This study utilizes the Rice Plant Diseases v9, Rice Plant Diseases v8 datasets,and Rice Leaf Spot Disease Dataset, labeled by agricultural specialists, aimed at offering varied visual data for model training and assessment.The images are field-captured RGB color images, resized to 640×640 px. To ensure uniformity and compatibility with the model, normalization was applied during the preprocessing stage. The Rice Plant Diseases v9 and Rice Plant Diseases v8 datasets cover four rice leaf diseases: Bacterial leaf blight, Grassy stunt, Rice blast, and Tungro, the Rice Leaf Spot Disease Dataset covers eight rice leaf classes: Bacterial leaf blight(BLB), Brown Spot, Healthy,*

*Leaf Blast, Leaf Scald, Leaf Spot, Neck Blast, and Rice Hispa, all of which display unique visual signatures essential for computer vision tasks. These annotated features are critical for training models to accurately classify and assess the severity of different rice leaf diseases.”*

*The following paragraph has been updated to section 4.1 (on page 17).*

*“Table 3 displays the Rice Leaf Spot Disease Dataset (RLSD), which is employed in this research to evaluate model generalization across a diverse range of disease categories. The dataset contains a total of 3567 images (6122 annotations) distributed across eight distinct classes. The training dataset includes 531 labels for BLB, 1148 for Brown Spot, 662 for Healthy samples, 634 for Leaf Blast, 452 for Leaf Scald, 572 for Leaf Spot, 770 for Neck Blast, and 697 for Rice Hispa. Meanwhile, the validation dataset comprises 58 labels for BLB, 128 for Brown Spot, 76 for Healthy samples, 63 for Leaf Blast, 60 for Leaf Scald, 66 for Leaf Spot, 100 for Neck Blast, and 105 for Rice Hispa.”*

2. Relocated Experimental Setup: We moved the detailed Hardware/Software Configuration Tables and the Hyperparameter Settings Table from the main text to the Supplementary Materials (Supporting information), as below:

*Supporting Information file has a legend listed in the manuscript after the references list. The following paragraph has been updated to section Supporting information on page 32.*

*“Supporting information*

*S1 Table. Hardware configuration*

*S2 Table. Software configuration.*

*S3 Table. Key hyperparameter settings.”*

*supporting information files*

3. Simplified Metrics: We removed the standard mathematical formulas for Precision, Recall, and mAP in original Section 4.3. These changes have significantly reduced the length of the main text and improved the focus on our proposed core innovations.

Revision Report:

Article title: Multi-scale closed-loop tuning via spatial-frequency collaborative sensitivity for rice leaf disease detection

Dear reviewer,

We sincerely appreciate the reviewer for thorough assessment and positive comments regarding the innovation and empirical validation of our work. We have carefully addressed the concerns regarding the model architecture, input preprocessing, generalization, and writing quality. Please find our detailed responses below.

# Reviewer 2:

1) Comment 1:The first concern is about the model architecture. In figure 1, the green dot lines show the feature feedback process. However, there forms a cycle in the data flow and this will lead to collapse in gradient descent. The authors are suggested to provide more explanation about how to solve this problem in the training/fine-tuning process or provide clearer dataflow of the architecture.

**Response:** We sincerely appreciate the reviewer’s valuable suggestion, thank you for pointing out the potential ambiguity of the feedback connections in Fig 1. We agree that a cyclic computational graph could cause instability during backpropagation. In our implementation, the proposed “closed-loop” tuning is not realized as a true cyclic graph. Instead, it is implemented as a two-pass (unrolled) refinement with a stop-gradient (SG) operation on the feedback features, which prevents gradients from flowing through the feedback path and therefore avoids any cyclic dependency in gradient descent.

Specifically, we first perform a standard forward pass to obtain the multi-scale semantic outputs

{O_S_,O_M_,O_L_}. These outputs are then detached (stop-gradient) and only used as guidance signals to calibrate the auxiliary shallow features in the subsequent refinement step. The detection loss is computed on the final predictions, while the first-pass outputs are used solely for feature guidance. To improve clarity, we have revised the relevant paragraph in the updated version. The modified text can now be found on page 13-15 in section 3.4.

We have revised Fig 1 and Fig 5 by explicitly marking the feedback edges with “SG” to indicate stop-gradient on{O_S_,O_M_,O_L_}, clarifying that the feedback is guidance-only and does not form a cyclic backpropagation graph.

Expanded Section 3.4 (CLTB) with a clearer description of the training/inference dataflow, and added pseudocode (Algorithm 1) to present the unrolled two-step procedure and the exact locations where SG is applied.

These revisions clarify that the feedback mechanism does not create a gradient loop and thus does not lead to collapse in gradient descent.

*Fig 1. MCCA-YOLO model architecture*

*Fig 5. Closed-loop tuning Bi-backbone network*

The following paragraph has been updated to section 3.4 (on page 13-15).

2) Comment 2: The second concern is about the input of the framework. Since there are images with different resolutions serving as input. In figure 1, it seem that the left input images for C1 are the super-resolutioned version or a patch of the input images on the right for P1. Thus, the problem is where does the author obtain or how to decide which part should be extracted to perform as these image patchs? The authors are suggested to give more explaination about the image pre-processing part.

**Response:** We apologize for any ambiguity in the original manuscript that may have led to misunderstanding. We appreciate the reviewer for pointing out this ambiguity. The input to our framework is uniform. We do not use super-resolution or separate patch extraction algorithms as input preprocessing. The "patches" illustrated in the original figure were intended to four distinct categories of disease, not distinct input crops.

**Revision:** In the revised Figure 1, the diagram now displays representative sample images corresponding to the four distinct disease categories (e.g., Bacterial leaf blight, Blast, etc.) as the network input. This visualization serves to illustrate the diversity of disease types handled by the model, rather than a patch-based preprocessing step. We have also added a clear description in As noted in Section 4.1, we apply only standard resizing and normalization as preprocessing.

*Fig 1. MCCA-YOLO model architecture*

*The following paragraph has been added to section 4.1 (on page 17).*

*“The images are field-captured RGB color images, resized to 640×640 px. To ensure uniformity and compatibility with the model, normalization was applied during the preprocessing stage.”*

3) Comment 3: In this paper, both rice plant diseases dataset v8 and v9 are from Roboflow, likely lab-collected images, which are limited to some specified regions or type of rice plants. Real-world field conditions (motion blur, occlusion, varying lighting, mixed diseases) may not be adequately represented. Different regions may also face different diseases. Thus, the generalizability of the proposed method is not adequatly investigated and it is suggested to include more real-world datasets and use cross-domain evaluation (train on v8 and test on real-world images).

**Response:** We sincerely regret the oversight in the original submission. Thank you for pointing outthis error. As suggested, we strictly followed the reviewer’s suggestion to rigorously validate the model's generalizability and robustness in real-world field conditions. We have made comprehensive revisions in the following aspects:

1. First, although the Rice Plant Diseases v8 and v9 datasets are hosted on Roboflow, the images contained within them were indeed captured in real-world field environments, rather than controlled laboratory settings. However, we fully agree with the reviewer that broader cross-domain evaluation is necessary to prove the model's robustness against varying conditions.

2. Inclusion of the RLSD Dataset: To address the concern about "limited regions" and "complex field conditions," we have expanded our evaluation to include the RLSD (Real-life Rice Leaf Diseases) dataset. This dataset features images collected in complex field environments with diverse backgrounds. We have updated Table 3 to include the detailed description of the RLSD dataset, as below:

*The following paragraph has been updated to section 4.1 (on page 18).*

*“Table 3 displays the Rice Leaf Spot Disease Dataset (RLSD), which is employed in this research to evaluate model generalization across a diverse range of disease categories. The dataset contains a total of 3567 images (6122 annotations) distributed across eight distinct classes. The training dataset includes 531 labels for BLB, 1148 for Brown Spot, 662 for Healthy samples, 634 for Leaf Blast, 452 for Leaf Scald, 572 for Leaf Spot, 770 for Neck Blast, and 697 for Rice Hispa. Meanwhile, the validation dataset comprises 58 labels for BLB, 128 for Brown Spot, 76 for Healthy samples, 63 for Leaf Blast, 60 for Leaf Scald, 66 for Leaf Spot, 100 for Neck Blast, and 105 for Rice Hispa.”*

3. Comprehensive Cross-Domain Experiments: We have conducted extensive quantitative experiments on the RLSD dataset to verify generalizability:

Ablation Studies: We added results on the RLSD dataset to Table 6, Table 9, and Table 12, confirming the effectiveness of our proposed modules (TSSFE, DHCA, etc.) across different domains.

Comparative Experiments: We extended the comparison with state-of-the-art models to include the RLSD dataset, as presented in Table 15, Table 18, and Table 21, as shown below. The results demonstrate that MCCA-YOLO maintains superior performance even on this new, challenging dataset.

4. Qualitative Analysis on Unseen Data: We have added a new figure [8][9][10] to present a cross-domain qualitative analysis. This analysis utilizes images from the test set (which the model had never seen during training) to visually demonstrate that MCCA-YOLO maintains high-level detection capabilities in unfamiliar environments with complex backgrounds.

*The following paragraph has been added to section 4.3 (on page 25-26).*

*“To further evaluate the robustness and cross-dataset generalization of MCCA-YOLO, we conduct a qualitative comparison on three datasets: Rice Plant Diseases v9, Rice Plant Diseases v8, and RLSD. Fig 8, Fig 9, and Fig 10 present representative cases with typical challenges, including tiny lesions with low contrast (Rice Blast), elongated disease regions in cluttered backgrounds (Bacterial Leaf Blight), and large-area symptoms with ambiguous boundaries (Leaf Scald). We compare MCCA-YOLO with several competitive detectors (YOLOv5s, YOLOv8s, BGF-YOLO, RT-DETR, and Mamba YOLO) under the same visualization settings. As shown in Fig 8, the rice blast lesion is small and visually similar to surrounding textures, which often leads to imprecise localization or missed detections for baseline models. In Fig 9, bacterial leaf blight exhibits long stripe-like patterns across leaves, competing methods tend to produce fragmented predictions, duplicated boxes, or over-extended boxes covering irrelevant regions. In contrast, MCCA-YOLO yields more consistent localization with tighter bounding boxes around the actual diseased areas. For RLSD in Fig 10, where symptoms cover a relatively large region and the boundary is weak, several methods either under-localize or over-localize, whereas MCCA-YOLO better balances completeness and precision. Overall, these visual results indicate that MCCA-YOLO is more robust to variations in scale, appearance, and background complexity, demonstrating strong generalization across different agricultural datasets.”*

*Fig 8. Visualization of detection results for Dataset V9.*

*Fig 9. Visualization of detection results for Dataset V8.*

*Fig 10. Visualization of detection results the RLSD Dataset.*

5. Real-World Deployment Verification: Furthermore, as described on page26-27 in Section 4.4, we deployed the trained model on an Atlas 200 DK edge computing device. We conducted real-time detection tests under varying illumination and motion conditions. The successful deployment further validates the model's applicability and robustness in dynamic, real-world agricultural scenarios.

4) Comment 4: The manuscript suffers from numerous writing flaws.

(1) In the manuscript, all abbreviations and their full forms should be defined at first use. But there are several abbreviations whoes full forms are not provided, e.g. MCCA-YOLO, what does MCCA mean?; C2F; and some of the abbreviations used in evaluation metrics tables cannot be found in previous contents etc.

**Response:** We sincerely regret the oversight in the original submission, thank you for pointing outthis error. We apologize for the writing and formatting errors. We have conducted a meticulous proofreading of the entire manuscript and made the following corrections:

1. Abbreviations: We have ensured that all abbreviations are defined at their first occurrence. For example, we explicitly defined MCCA (Multi-scale Closed-loop Collaborative Attention) on page 1-3 in the Abstract and Introduction, and defined C2f (CSPBottleneck with 2 convolutions) on page 7 in Section 3.1.

*The following paragraph has been updated to section abstract (on page 1).*

*“This paper proposes a Multi-scale closed-loop tuning via spatial-frequency collaborative sensitivity for rice leaf disease detection (MCCA-YOLO) model with a multiscale closed-loop tuning and spatial-frequency collaborative attention mechanism for the early detection and classification of rice crop diseases”*

*The following paragraph has been updated to section introduction (on page 3).*

*“Inspired by previous research, we propose a deep neural network model named Multi-scale closed-loop tuning via spatial-frequency collaborative sensitivity for rice leaf disease detection (MCCA-YOLO)”*

*The following paragraph has been updated to section 2.2 (on page 5).*

*“However, it operates only at a single scale and suffers from a domain Global Average Pooling (GAP) when applied to CNN-based contexts”*

*The following paragraph has been updated to section 3.1 (on page 7).*

*“In the CSPDarknet53 to 2-Stage FPN (C2F) model”*

*The following paragraph has been updated to section introduction (on page 2).*

*“Self-Attention and Convolution (ACmix)”*

*The following paragraph has been updated to section 2.2 (on page 5).*

*“Unmanned Aerial Vehicle (UAV-DETR)”*

(2) The evaluation metrics are not properly formatted. e.g. In Table 7, why 0.895 is presented in bold format? The results of MCCA-YOLO are not the same if Table 9 and Table 15. The table title of Table 16 should be v8.

**Response:** We sincerely regret the oversight in the original submission, thank you for pointing outthis error. We apologize for the writing and formatting errors. We have conducted a meticulous proofreading of the entire manuscript and made the following corrections:

Table Formatting:

We corrected the bold formatting in Table 5 (original manuscript Table 7) to ensure only the best results are highlighted.

We verified and ensured consistency between the results in Table 10 and Table 19 (original manuscript Table 9 and Table 15) .

We corrected the caption of Table 17 (original manuscript Table 16) to explicitly refer to the "Rice Plant Diseases v8" dataset.

(3) The images are not positioned with their titles in pdf file and the hypper ref link of these images lead to titles without images, which makes the paper hard to read.

**Response:** We thank the reviewer for the comment, thank you for your comment regarding the figure placement in the PDF and the figure hyperlinks, which indeed can affect readability. The PLOS ONE system requires figures to be uploaded as individual files, and they appear as such in the auto-generated review PDF. they are typically compiled and placed at the end of the document by default rather than displayed next to their captions in the main text. As a result, captions may appear in the manuscript body while the corresponding images are collected at the bottom, and the hyperlinks may direct to the caption location instead of an in-text embedded figure.To help readers navigate the manuscript more easily, we have checked that all figures are clearly and consistently cited in the text (e.g., “Fig X”) and that each caption matches the correct uploaded figure file.

Revision Report:

Article title: Multi-scale closed-loop tuning via spatial-frequency collaborative sensitivity for rice leaf disease detection

Dear reviewer,

We would like to express our sincere gratitude to the reviewer for the encouraging comments and the recognition of our work's value in agricultural disease diagnosis. Thanks for taking your precious time to review this manuscript. We have thoroughly revised the manuscript based on your comments. The revised texts are marked in red in the manuscript. Below we provide our responses point by point.

# Reviewer 3:

1) Comment 1: Lack of ablation experiments for TSSFE in Backbone based on rice plant diseases dataset v8.

**Response**:We greatly appreciate the reviewer’s suggestion to strengthen our validation. We have performed the additional ablation study for the TSSFE module using the Rice Plant Diseases v8 dataset and RLSD dataset. The results are consistent with those from the v9 dataset, further confirming the module's effectiveness. We have added these results in Table 8 and Table 9 in the revised manuscript.

*The following paragraph has been added to section 4.2 (on page 20).*

*“When TSSFE is restricted to P5, precision increases by 1.3% compared to the YOLOv8s baseline on the v9 dataset; however, mAP@50 shows a minimal improvement of 0.1% due to the underutilization of fine-grained shallow features. Implementing TSSFE across P2˜P4 better utilizes these shallow features, resulting in an additional 1% improvement in mAP@50 relative to the P5 configuration on the v9 dataset. However, it is worth noting that on the v8 and RLSD datasets, the P2˜P4 configuration demonstrated performance comparable to or slightly lower than the P5 setup. Furthermore, although extending implementation across all levels (P2˜P5) enhances information flow, it introduces feature interference, causing mAP@50 to decrease to 86.1% on the v9 dataset. Consequently, the P3˜P5 strategy was selected as it achieves an optimal balance between texture detail and semantic context while minimizing low-level noise. This approach delivers superior overall performance across all datasets. Compared to the baseline, mAP@50 improved by 1.4%, 1.4%, and 1.5% on the v9, v8, and RLSD datasets, respectively. Similarly, mAP@50:95 increased by 1.8%, 1.7%, and 0.7% on these datasets, requiring only 1.5M additional parameters. These consistent results indicate that the fusion of mid-to-high-level features proves particularly effective and robust for rice disease spot detection.”*

2) Comment 2: Check the use and interpretation of the α variable in the formula.

**Response:** We sincerely regret the oversight in the original submission. Thank you for identifying this ambiguity. We realized that the symbol α was used to denote two different parameters: the attention weights in the TSSFE module (Formula 22) and the logic values in the SWFN (Formula 32). To avoid confusion, we have changed the notation in the SWFN module (Formula 32) from α to β. We have ensured consistent variable notation throughout the "Method" section 3.5.

3) Comment 3: There are several details in the paper, such as inconsistent variable formats, layout of formulas, the lack of punctuation.

**Response:** We sincerely regret the oversight in the original submission. Thank you for pointing outthis error. As suggested, we carefully proofread the entire manuscript and systematically corrected these presentation issues. Specifically, we unified variable and symbol formatting across the paper. All multiplication symbols in the text have been standardized.

4) Comment 4: Check the lowercase at the beginning of paragraphs and the details of the text in the tables.

**Response:** We sincerely regret the oversight in the original submission. Thank you for pointing outthis error. We have corrected the capitalization errors found in the text. We re-checked all paragraph openings and fixed any instances where a paragraph began with a lowercase letter, ensuring consistent sentence-case style throughout the manuscript.

In addition, we thoroughly reviewed all tables for formatting and content consistency, including table titles, dataset labels, metric names, and numeric entries. As a concrete example, we corrected table-detail inconsistencies by ensuring dataset identifiers in table titles/subtitles are consistent. We also re-checked bold/highlight rules so that emphasis is applied consistently.

5) Comment 5: The DA (Directional Attention) module should have detailed illustrations.

**Response:** We thank the reviewer for the comment. We have created a new, detailed schematic diagram for the Deformable Hybrid Collaborative Attention (DHCA) module, specifically highlighting the Directional Attention (DA) component. The new figure visually details the parallel processing of horizontal (1×3), vertical (3×1), and diagonal convolutions. This is presented as Fig 3 in the revised manuscript, as below:

*Fig 3. Direction attention architecture*

6) Comment 6: The flowchart should have appropriate annotations, such as the experimental categories shown in Figure 9, the input and output of the flowchart.

**Response:** We thank the reviewer for the comment. We have revised the overall architecture flowchart (Figure 2 and Figure 4) to explicitly label the "Input Image" and the "Detection Output" . Additionally, for the deployment result (Figure 12), we have added clear annotations indicating the specific disease categories detected in the real-world samples, as below:

*Fig 4. Two-stage spatial frequency enhancement module*

*Fig 2. Deformable hybrid collaborative attention architecture*

*Fig 12. Local deployment of the model and visualization of detection results*
