# Supplementary material for: Multi-scale closed-loop tuning via spatial frequency collaborative sensitivity for rice leaf disease detection
Source: PLoS One. 2026 Jun 18;21(6):e0351727. doi: 10.1371/journal.pone.0351727 (PMC13278584; doi:10.1371/journal.pone.0351727)
Supplement: Attachment — Submitted filename: Response_to_Reviewers_auresp_3.docx [file pone.0351727.s011.docx]

Revision Report:

Article title: Multi-scale closed-loop tuning via spatial-frequency collaborative sensitivity for rice leaf disease detection

Response to the Academic Editor

Dear Academic Editor,

We sincerely thank you for your positive evaluation of our manuscript and for your valuable suggestion regarding language polishing. In response to your comment, we carefully proofread the entire manuscript and revised the grammar, sentence structure, terminology, and linguistic expression to improve clarity, fluency, and academic professionalism.

Comment:

Please focus on polishing the grammar and linguistic expression to ensure the highest professional standard before formal acceptance.

**Response:**

Thank you very much for your helpful suggestion. We have carefully revised the manuscript throughout and highlighted all language-related changes in the marked-up version. The main revisions are listed below.

The following text has been revised in the Abstract section on page 1:

"making stable yields and quality improvements vital for food security and sustainable agricultural development."

The following text has been revised in the Abstract section on page 1:

"Early infections of rice leaf diseases often exhibit subtle symptoms"

The following text has been revised in the Abstract section on page 1:

"A Multi-scale closed-loop tuning via spatial frequency collaborative sensitivity (MCCA-YOLO) model has been proposed in this paper with a multiscale closed-loop tuning and spatial frequency collaborative attention mechanism for the early detection and classification of rice crop diseases."

The following text has been revised in the Abstract section on page 1:

"feature adaptation""comprehensive" " achieves a mean average precision" "demonstrates"

The following text has been revised in the Introduction section on page 1:

"Rice is a fundamental cereal crop worldwide, serving as the primary dietary staple for a large portion of the global population."

The following text has been revised in the Introduction section on page 1:

"However, rice plants are highly susceptible to various pathogenic infections during their growth cycle."

The following text has been revised in the Introduction section on page 1:

"degrade"

The following text has been revised in the Introduction section on pages 1–2:

"Timely and accurate detection methods for rice leaf diseases are essential for developing effective management strategies and targeted control measures."

The following text has been revised in the Introduction section on page 2:

"This framework significantly improves both detection accuracy and generalization in grain pest identification tasks." "Li et al."

The following text has been revised in the Introduction section on page 2:

"The first module extracts discriminative features from three distinct scales through Local Binary Pattern (LBP), grayscale, and Histogram of Oriented Gradients (HOG) image representations. The second module hierarchically integrates semantic global and local features."

The following text has been revised in the Introduction section on page 2:

"Existing multi-scale fusion strategies frequently encounter information redundancy and scale conflicts, particularly when processing early-stage subtle symptoms on rice leaves, where symptom manifestations exhibit weak correlations across scales."

The following text has been revised in the Introduction section on page 3:

"The proposed detection network consists of a closed-loop tuning Bi-backbone feature extraction network for system self-verification feedback, and a multi-scale feature weighted and deformable hybrid attention bidirectional feature pyramid fusion network to achieve adaptive extraction and cross-scale fusion of rice leaf lesion features."

The following text has been revised in the Introduction section on page 3:

"enhances the texture and edge details of rice leaves, and improves their feature representation."

The following text has been revised in the Introduction section on page 3:

"The experimental results show that our method outperforms other state-of-the-art approaches."

The following text has been revised in the Introduction section on page 3:

"The model is deployed for inference applications on edge devices."

The following text has been revised in the Introduction section on page 3:

"The remainder of this paper is organized as follows. Section 2 reviews prior research on object detection, attention mechanisms, and rice leaf disease detection. Section 3 presents the proposed MCCA-YOLO detection network in detail, including its overall architecture, deformable hybrid collaborative attention mechanism, two-stage spatial frequency enhancement module, closed-loop tuning dual-backbone network, and scale-weighted fusion network. Section 4 evaluates the algorithm on several rice leaf disease datasets and discusses the experimental results. Finally, Section 5 summarizes the main findings and concludes the paper."

The following text has been revised in Section 2.1 on page 3:

"Representative one-stage detectors include SSD and the YOLO series, while Mask R-CNN is a notable two-stage extension of the Faster R-CNN framework."

The following text has been revised in Section 2.1 on page 4:

"The latter strategy seeks to increase representational capacity not through depth, but via architecture. A prominent example is the Composite Backbone Network (CBNet)."

The following text has been revised in Section 2.1 on page 4:

"These multi-scale features are then fused and enhanced by a neck module to form a more discriminative representation for detection."

The following text has been revised in Section 2.1 on page 4:

"PANet augments FPN with an additional bottom-up path, shortening the information flow between low-level and high-level features and thus improving the representation of small objects."

The following text has been revised in Section 2.2 on page 4:

"They operate by adaptively weighting feature responses, emphasizing informative regions or channels while suppressing less useful ones, which has led to substantial gains in various vision tasks including object detection. The seminal Squeeze-and-Excitation (SE) network models inter-channel dependencies, recalibrating channel-wise feature responses to boost representational capacity."

The following text has been revised in Section 2.2 on page 5:

"To incorporate spatial information, the Convolutional Block Attention Module (CBAM) sequentially applies channel and spatial attention sub-modules."

The following text has been revised in Section 2.2 on page 5:

"To better capture global patterns, researchers have turned to the frequency domain, complementing spatial attention mechanisms."

The following text has been revised in Section 2.2 on page 5:

"Other works explore hybrid attention in different architectures. For instance, the FSTA SNN uses spectral statistics to guide spatial and temporal attention, though it is designed for spiking neural networks and operates at a single scale."

The following text has been revised in Section 2.2 on page 5:

"Furthermore, prevalent attention mechanisms often employ fixed or heuristic-based fusion strategies, failing to dynamically recalibrate feature responses based on the varying characteristics of different lesions and image contexts."

The following text has been revised in Section 2.2 on page 5:

"To overcome these challenges, we propose two core components: a Deformable Hybrid Collaborative Attention (DHCA) mechanism and a Two-Stage Spatial Frequency Enhancement (TSSFE) module."

The following text has been revised in Section 2.2 on page 6:

"These modules form the core of our proposed MCCA-YOLO framework, which is tailored for robust rice leaf disease detection."

The following text has been revised in Section 2.3 on page 6:

"Subsequent work, like that of Sharma et al., applied convolutional neural networks (CNNs) to detect a broader set of rice diseases and pests."

The following text has been revised in Section 2.3 on page 6:

"A notable example is UAV T-YOLO-Rice, a lightweight detector based on Tiny YOLOv4. It reported 86% mAP on several diseases and offered a favorable speed-accuracy trade-off, though its older architecture may not fully leverage recent advances."

The following text has been revised in Section 2.3 on page 6:

"To address these limitations, we propose MCCA-YOLO, an enhanced framework based on YOLOv8. Our primary objective is to achieve superior accuracy while maintaining high computational efficiency suitable for practical deployment. This is achieved through several key innovations integrated into the YOLOv8 architecture, designed specifically to enhance robustness against field complexities such as cluttered backgrounds and varying leaf geometries."

The following text has been revised in Section 3.1 on page 6:

"This study proposes a novel framework named Multi-scale Closed-loop tuning via Collaborative spatial-frequency sensitivity Attention YOLO (MCCA-YOLO) for rice leaf disease detection."

The following text has been revised in Section 3.1 on page 6:

"These feedback features are utilized to calibrate the backbone network. This refinement process involves upsampling the features from the neck network and then applying a 1×1 convolutional projection on the relevant layers."

The following text has been revised in Section 3.1 on page 7:

"Following this closed-loop calibration, the calibrated values C3, C4, and C5 are upsampled, respectively, and then fed into the main backbone through a 1×1 convolutional layer."

The following text has been revised in Section 3.1 on page 7:

"The multi-scale features are then integrated in the Scale-Weighted Fusion Neck. Here, the Scale-Weighted Fusion Entity (SWFE) module performs adaptive weighted fusion of cross-layer features, explicitly learning the contribution weight of each scale to lesion detection."

The following text has been revised in Section 3.1 on page 7:

"This design enables the system to maintain a lightweight architecture while preserving high sensitivity to minor disease manifestations and ensuring robust performance in complex environments."

The following text has been revised in Section 3.2 on page 7:

"When detecting rice leaf diseases in natural settings, the leaves themselves are often deformed due to wind and gravity, exhibiting significant bending and tilting. Lesion spots on leaves also tend to arrange in linear patterns, either along the leaf’s longitudinal axis or transverse to it."

The following text has been revised in Section 3.2 on page 7:

"To address these challenges, we propose a Deformable Hybrid Collaborative Attention (DHCA) mechanism and integrate it into the C2F component of the YOLOv8 feature fusion network, forming a new bottleneck-DHCA module."

The following text has been revised in Section 3.2 on page 7:

"Following the DCN, the module incorporates two parallel branches: the direction-aware attention branch and the channel-self-attention branch.The features from both branches are fused via a learnable gating mechanism. Furthermore, residual connections are incorporated to stabilize gradient flow. This design enables the module to effectively adapt to the diverse shapes of leaves."

The following text has been revised in Section 3.2.1 on page 7:

"Consequently, the sampling grid can adaptively deform to fit the local geometric structure of the input, such as leaf curvature and vein orientation."

The following text has been revised in Section 3.2.1 on page 8:

"Thus, the output feature map A” “ is computed as shown in Equation (1)."

The following text has been revised in Section 3.2.1 on page 8:

"Each modulation coefficient weights the corresponding transformed feature. Since the deformed sampling location” "typically has non-integer coordinates, we use bilinear interpolation to compute its feature value from the four nearest pixels in the input feature map."

The following text has been revised in Section 3.2.2 on page 8:

"Following alignment via DCNv4 to compensate for leaf bending and tilting, the pixel positions in the feature maps are geometrically corrected. However, alignment alone is insufficient to capture the linear distribution of lesions along specific directions. To address this, we introduce a Directional-Attention (DA) module, which processes the aligned features using dedicated filters oriented horizontally, vertically, and diagonally."

The following text has been revised in Section 3.2.3 on page 9:

"This weighting vector is then used to recalibrate the input features, performing channel-wise scaling that can potentially amplify lesion-related features and suppress responses from healthy regions.” "First, two complementary feature representations, AK and AQ, are generated by applying depthwise separable convolutions with different kernel sizes (3×3 and 5×5, respectively) to capture multi-scale context."

The following text has been revised in Section 3.2.3 on page 9:

"A_K_ and A_Q_ are concatenated along the channel dimension and then passed to a two-layer 1×1 convolutional module for attention embedding."

The following text has been revised in Section 3.2.3 on page 9:

"The second 1×1 convolution then expands the channel count to n²C, where n is the side length of a local n×n window. The output of this convolution represents the dynamic weight for each channel within its corresponding n×n local window."

The following text has been revised in Section 3.2.3 on page 10:

"The channel-attention map As is applied to Av through element-wise multiplication, producing the refined features Vweight."

The following text has been revised in Section 3.2.4 on page 10:

"The outputs of the two branches, denoted as A_dir_ and A_channel_, are first concatenated. The combined tensor is then fed into a gating function. The gating function consists of a 1×1 convolution followed by a Sigmoid function, thereby producing a gating tensor G."

The following text has been revised in Section 3.2.4 on page 11:

"Finally, the gated fusion of the two branches is added to the original input feature map via a residual connection, producing the final output Y of the DHCA module."

The following text has been revised in Section 3.3 on page 11:

"Under complex field conditions involving variable lighting, shading, and natural heterogeneity, lesions on rice leaves often exhibit a distinct set of characteristics."

The following text has been revised in Section 3.3 on page 11:

"Conventional frameworks that rely solely on spatial convolutions or single-domain frequency attention struggle to capture this complementary information, which limits their performance in early disease detection."

The following text has been revised in Section 3.3 on page 11:

"In the first stage, the input feature map m is reshaped via adaptive pooling to obtain m_g_, which matches the required dimensions for the subsequent 2D DCT."

The following text has been revised in Section 3.3 on page 11:

"The 2D DCT basis function is defined in formula (15). The resulting frequency-domain representation for the i-th block, denoted as F_reqi_, is then computed according to formula (16)."

The following text has been revised in Section 3.3 on page 12:

"TSSFE is designed to jointly enhance high-frequency textures and low-frequency contours through a two-stage process of spatial-frequency collaborative modeling and dynamic frequency-band weighting."

The following text has been revised in Section 3.3 on page 12:

"The residual structure essentially circumvents the suppression of original input features. The module is designed to be computationally efficient and can be readily integrated into existing network architectures."

The following text has been revised in Section 3.4 on page 12:

"In rice leaf spot monitoring, traditional single-path convolution methods often struggle to effectively mitigate error propagation."

The following text has been revised in Section 3.4 on page 13:

" as illustrated in formula (23)."

The following text has been revised in Section 3.4 on page 13:

"Consequently, deep semantic analysis in later layers alone cannot fully recover the fine-grained edge information lost in early stages."

The following text has been revised in Section 3.4 on page 13:

"These coarse outputs serve solely as semantic guidance. Critically, they are detached using a stop-gradient operator. This ensures that no gradients flow from this feedback branch back to the main backbone during training."

The following text has been revised in Section 3.4 on page 13:

"The calculation process can be expressed as formula (24)."

The following text has been revised in Section 3.4 on page 13:

"These processed semantic features are summed to form a residual signal, which is added to the auxiliary feature map to produce the refined feature. This update is defined in formula (25)"

The following text has been revised in Section 3.4 on page 14:

"as expressed in formula (26)." " as expressed in formula (27)" "as shown in formula (28)." "as shown in formula (29)." "Here, N(·) denotes the neck module."

The following text has been revised in Section 3.5 on page 16:

"Under natural lighting and wind, rice leaf lesions are characterized by their small size, varied morphology, and low contrast."

The following text has been revised in Section 3.5 on page 16:

"For fine-grained rice disease detection, this uniform weighting scheme is suboptimal."

The following text has been revised in Section 3.5 on page 16:

"The Asymptotic Feature Pyramid Network (AFPN) addresses this by employing a fully connected fusion scheme among features P3, P4, and P5. However, the direct fusion between features with extreme scale differences can be problematic due to their significant semantic and resolution gap."

The following text has been revised in Section 3.5 on page 16:

"SWFN takes the feature maps from the backbone as input. 1×1 convolutions are applied to each to reduce their channel dimensions by half for efficient processing." "When"

The following text has been revised in Section 4.1 on page 17:

"We employ three publicly available datasets for model training and evaluation: Rice Plant Diseases v9, Rice Plant Diseases v8, and the Rice Leaf Spot Disease dataset."

The following text has been revised in Section 4.1 on page 17:

"For each dataset, we randomly partition the images into a training set and a test set using a 9:1 ratio."

The following text has been revised in Section 4.1 on page 17:

"We employed early stopping with a patience of 50 epochs and set the maximum number of training epochs to 150."

The following text has been revised in Section 4.2 on page 17–18:

"We use the standard YOLOv8 model as our baseline and incrementally integrate the proposed modules to assess their individual contributions. We conduct a series of ablation studies to evaluate the proposed components, including Deformable Convolution (DCN), Channel Attention, Direction-Aware Attention (DA), Gating mechanisms, and their integration into the Deformable Hybrid Collaborative Attention (DHCA) module."

The following text has been revised in Section 4.2 on page 18:

"Params" "This yields mAP_@50_ improvements of +1.3% (v9), +0.9% (v8), and +0.7% (RLSD) over the baseline, while reducing parameters by 0.5M."

The following text has been revised in Section 4.2 on page 18:

"This configuration improves mAP_@50_ by +1.8% (v9), +1.2% (v8), and +1.2% (RLSD), at the cost of a 0.6M parameter increase relative to the DCN-only variant."

The following text has been revised in Section 4.2 on page 18:

"Finally, integrating the complete DHCA module yields the most significant gains, with mAP_@50_ improvements of +2.7% (v9), +2.2% (v8), and +1.9% (RLSD) over the baseline, while adding only 0.9M parameters."

The following text has been revised in Section 4.2 on page 19:

"On the v8 and RLSD datasets, however, the P2-P4 configuration performs comparably to or slightly below the P5-only setup."

The following text has been revised in Section 4.2 on page 20:

"The channel attention module SE improves mAP_@50_ by +0.8% (v9), +1.2% (v8), and +0.6% (RLSD) with an addition of 0.06M parameters. SEv2 yields gains of +0.2% (v9), +0.3% (v8), and +0.9% (RLSD) for 0.1M additional parameters. ECA, while adding only 0.03M parameters, enhances mAP@50 by +0.9% (v9), +0.9% (v8), and +0.9% (RLSD)."

The following text has been revised in Section 4.2 on page 21:

"Our proposed SWFN selectively fuses context into P4. It achieves mAP_@50_ improvements of +1.4% (v9), +1.4% (v8), and +3.8% (RLSD) over the baseline, adding only 1.3M parameters. Model F also achieves high recall (84.4% v9, 88.2 % v8, 57.1 % RLSD) and precision (86.6% v9, 89.9% v8, 68.8% RLSD). "

The following text has been revised in Section 4.3 on page 21:

"Table 6 compares the proposed MCCA-YOLO with several state-of-the-art detectors—Faster R-CNN, YOLOv5s, YOLOv8, BGF-YOLO, and RT-DETR—on the rice disease datasets."

The following text has been revised in Section 4.3 on page 22:

"On the v9 dataset, MCCA-YOLO achieves the highest mAP_@50_ (0.904) and mAP_@50:95_ (0.637) among all compared models."

The following text has been revised in Section 4.3 on page 22:

"On the more challenging RLSD dataset, MCCA-YOLO achieves a precision of 0.682 and a recall of 0.574."

The following text has been revised in Section 4.3 on page 22:

"MCCA-YOLO has 35.6M parameters. The consistent and substantial accuracy gains it delivers across all three datasets demonstrate a favorable accuracy-complexity trade-off compared to other high-performance models."

The following text has been revised in Section 4.4 on page 23:

"Detection results from MCCA-YOLO are compared with those from several state-of-the-art detectors (YOLOv5s, YOLOv8s, BGF-YOLO, RT-DETR, Mamba YOLO) under identical conditions."

The following text has been revised in Section 4.4 on page 23:

"For the elongated Bacterial Leaf Blight pattern, some detectors produce fragmented or over-extended bounding boxes that do not align well with the true lesion extent."

The following text has been revised in Section 4.4 on page 23:

"The baseline YOLOv8 achieves 652.93 FPS. After integrating the proposed modules, MCCA-YOLO’s inference speed is 242.05 FPS."

The following text has been revised in Section 4.5 on page 24:

"To validate the practical applicability of MCCA-YOLO, we deploy and evaluate it on the Huawei Atlas 200I DK edge AI platform."

The following text has been revised in Section 4.5 on page 24:

"In our testing pipeline, a camera captures images of rice leaves, which are then processed by the deployed MCCA-YOLO model. Detection results are rendered in real time on the device’s screen."

The following text has been revised in Section 4.5 on page 24:

"Figure.12 shows real-time detection visualizations for four major rice leaf diseases on the Atlas 200I DK."

The following text has been revised in Section 4.5 on page 24:

"These high-confidence predictions demonstrate the model’s robustness and suitability for deployment on resource-constrained edge hardware."

The following text has been revised in Section 4.5 on page 24:

"This deployment experiment validates the practical viability of MCCA-YOLO for real-time, in-field rice disease detection using edge computing devices."

The following text has been revised in the Conclusion section on page 25:

"To address this, we propose MCCA-YOLO, a novel YOLO-based architecture for automated rice leaf disease detection. It incorporates four key innovations to significantly enhance detection performance."

The following text has been revised in the Conclusion section on page 25:

"Extensive experiments on multiple rice disease datasets show that MCCA-YOLO achieves state-of-the-art detection accuracy and robustly handles the complexities of in-field imagery."

The following text has been revised in the Conclusion section on page 25:

"Future work will explore several promising directions to build upon this research."

In summary, we have thoroughly polished the grammar, terminology, and linguistic expression throughout the manuscript. All revisions have been highlighted in the marked-up manuscript, and a clean version has also been provided. We hope that the revised manuscript now meets the highest professional language standard required for formal acceptance.

Sincerely,

Yandong Song, Kang An, Lidong Wang, and Bin Zhou

On behalf of all authors
