# Supplementary material for: Multi-scale closed-loop tuning via spatial frequency collaborative sensitivity for rice leaf disease detection
Source: PLoS One. 2026 Jun 18;21(6):e0351727. doi: 10.1371/journal.pone.0351727 (PMC13278584; doi:10.1371/journal.pone.0351727)
Supplement: Peer review history

# Peer review: Decision Letter 0

**DOI:** 10.1371/journal.pone.0351727.r001

**Authors:** Yun Zhang

1 Dec 2025

PONE-D-25-58819

Multi-scale closed-loop tuning via spatial-frequency collaborative sensitivity for rice leaf disease detection

PLOS ONE

Dear Dr. An,

Thank you for submitting your manuscript to PLOS ONE. After careful consideration, we feel that it has merit but does not fully meet PLOS ONE’s publication criteria as it currently stands. Therefore, we invite you to submit a revised version of the manuscript that addresses the points raised during the review process.

Please submit your revised manuscript by Jan 15 2026 11:59PM. If you will need more time than this to complete your revisions, please reply to this message or contact the journal office at plosone@plos.org. When you're ready to submit your revision, log on to https://www.editorialmanager.com/pone/ and select the 'Submissions Needing Revision' folder to locate your manuscript file.

Please include the following items when submitting your revised manuscript:

- A rebuttal letter that responds to each point raised by the academic editor and reviewer(s). You should upload this letter as a separate file labeled 'Response to Reviewers'.
- A marked-up copy of your manuscript that highlights changes made to the original version. You should upload this as a separate file labeled 'Revised Manuscript with Track Changes'.
- An unmarked version of your revised paper without tracked changes. You should upload this as a separate file labeled 'Manuscript'.

If you would like to make changes to your financial disclosure, please include your updated statement in your cover letter. Guidelines for resubmitting your figure files are available below the reviewer comments at the end of this letter.

If applicable, we recommend that you deposit your laboratory protocols in protocols.io to enhance the reproducibility of your results. Protocols.io assigns your protocol its own identifier (DOI) so that it can be cited independently in the future. For instructions see: https://journals.plos.org/plosone/s/submission-guidelines#loc-laboratory-protocols. Additionally, PLOS ONE offers an option for publishing peer-reviewed Lab Protocol articles, which describe protocols hosted on protocols.io. Read more information on sharing protocols at https://plos.org/protocols?utm_medium=editorial-email&utm_source=authorletters&utm_campaign=protocols.

We look forward to receiving your revised manuscript.

Kind regards,

Yun Zhang

Academic Editor

PLOS ONE

Journal requirements:

When submitting your revision, we need you to address these additional requirements.

1. Please ensure that your manuscript meets PLOS ONE's style requirements, including those for file naming. The PLOS ONE style templates can be found at

https://journals.plos.org/plosone/s/file?id=wjVg/PLOSOne_formatting_sample_main_body.pdf and

https://journals.plos.org/plosone/s/file?id=ba62/PLOSOne_formatting_sample_title_authors_affiliations.pdf

2. Thank you for stating the following financial disclosure:

“This research was co-supported by Zhejiang Provincial Educational Science Planning

Project (No. 2024SCG027), Kang An.”

Please state what role the funders took in the study.  If the funders had no role, please state: "The funders had no role in study design, data collection and analysis, decision to publish, or preparation of the manuscript."

If this statement is not correct you must amend it as needed.

Please include this amended Role of Funder statement in your cover letter; we will change the online submission form on your behalf.

3. Thank you for stating the following in the Acknowledgments Section of your manuscript:

“This research was co-supported by Zhejiang Provincial Educational Science Planning

Project (No. 2024SCG027), Hangzhou Normal University Foundation.”

We note that you have provided funding information that is currently declared in your Funding Statement. However, funding information should not appear in the Acknowledgments section or other areas of your manuscript. We will only publish funding information present in the Funding Statement section of the online submission form.

Please remove any funding-related text from the manuscript and let us know how you would like to update your Funding Statement. Currently, your Funding Statement reads as follows:

“This research was co-supported by Zhejiang Provincial Educational Science Planning

Project (No. 2024SCG027), Kang An.”

Please include your amended statements within your cover letter; we will change the online submission form on your behalf.

If the reviewer comments include a recommendation to cite specific previously published works, please review and evaluate these publications to determine whether they are relevant and should be cited. There is no requirement to cite these works unless the editor has indicated otherwise.

Additional Editor Comments:

The paper proposes MCCA-YOLO, an enhanced YOLOv8-based object detection model tailored for early-stage rice leaf disease detection. The Innovative Architecture with Strong Motivation, and the Strong Empirical Validation give me a deep impression. However, this paper also has some weaknesses as illustrated by three reviewers. Given the reviews of three reviewers, I tend to give a major revision, and I hope authors can improve this paper to achieve the level of PLOS One.

[Note: HTML markup is below. Please do not edit.]

Reviewers' comments:

Reviewer's Responses to Questions

**Comments to the Author**

1. Is the manuscript technically sound, and do the data support the conclusions?

The manuscript must describe a technically sound piece of scientific research with data that supports the conclusions. Experiments must have been conducted rigorously, with appropriate controls, replication, and sample sizes. The conclusions must be drawn appropriately based on the data presented.

Reviewer #1: Yes

Reviewer #2: Yes

Reviewer #3: Yes

**********

2. Has the statistical analysis been performed appropriately and rigorously?

Reviewer #1: Yes

Reviewer #2: Yes

Reviewer #3: Yes

**********

3. Have the authors made all data underlying the findings in their manuscript fully available?

The PLOS Data policy requires authors to make all data underlying the findings described in their manuscript fully available without restriction, with rare exception (please refer to the Data Availability Statement in the manuscript PDF file). The data should be provided as part of the manuscript or its supporting information, or deposited to a public repository. For example, in addition to summary statistics, the data points behind means, medians and variance measures should be available. If there are restrictions on publicly sharing data—e.g. participant privacy or use of data from a third party—those must be specified.

Reviewer #1: Yes

Reviewer #2: Yes

Reviewer #3: Yes

**********

4. Is the manuscript presented in an intelligible fashion and written in standard English?

PLOS ONE does not copyedit accepted manuscripts, so the language in submitted articles must be clear, correct, and unambiguous. Any typographical or grammatical errors should be corrected at revision, so please note any specific errors here.

Reviewer #1: Yes

Reviewer #2: Yes

Reviewer #3: Yes

**********

5. Review Comments to the Author

Please use the space provided to explain your answers to the questions above. You may also include additional comments for the author, including concerns about dual publication, research ethics, or publication ethics. (Please upload your review as an attachment if it exceeds 20,000 characters)

Reviewer #1: Summary:

This paper presents a well-structured and logically coherent study addressing the critical need for early rice leaf disease detection. The proposed MCCA-YOLO model, with its closed-loop tuning architecture and adaptive feature fusion strategies, effectively targets the challenges of subtle early lesion identification and complex lesion morphology. Its strong experimental performance underscores its value, offering meaningful insights for agricultural disease diagnosis and contributing a practical reference to the field.

However, three areas require improvement:

1.Image Layout Misalignment: Several images in the paper exhibit misaligned layouts, which compromises visual clarity and may distract readers from key findings.

2. Insufficient Comparative Visuals: While quantitative results are compelling, additional images illustrating detection effects across diverse datasets would better demonstrate the model’s robustness and real-world applicability.

3. Excessive Length: The paper is overly lengthy; non-innovative details (e.g., routine method descriptions or standard experimental setup steps) should be relocated to supplementary materials to streamline the main text and enhance focus.

Overall, this work has significant merits, and addressing these minor flaws will further elevate its academic impact and readability.

Reviewer #2: **Summary:**

The paper proposes MCCA-YOLO, an enhanced YOLOv8-based object detection model tailored for early-stage rice leaf disease detection. The core innovations include:

1. Closed-loop tuning dual-backbone architecture: A feedback mechanism from the neck (feature fusion module) to an auxiliary backbone enables dynamic refinement of shallow features using high-level semantic cues.

2. Deformable Hybrid Collaborative Attention (DHCA): Integrates deformable convolution with directional attention (horizontal/vertical/diagonal) and channel-wise self-attention, gated by a learnable cross-branch fusion mechanism.

3. Two-Stage Spatial-Frequency Enhancement (TSSFE): Leverages Discrete Cosine Transform (DCT) to jointly model high-frequency textures (lesion edges) and low-frequency tone variations in the frequency domain, fused with spatial features.

4. Scale-Weighted Fusion Network (SWFN): Replaces standard PANet fusion with pixel-adaptive, softmax-normalized weights across P3–P5 scales to prioritize diagnostically relevant features.

Exprimental results demonstrate the effectiveness of the proposed method.

**Strengths:**

1. Innovative Architecture with Strong Motivation: The novelty is sufficient and the designed modules and techniques are effective.

2. Strong Empirical Validation: Experiments on two versions (v8 & v9) of a public dataset add robustness. Sufficient ablation studies validate each component.

**Weaknesses:**

1. The first concern is about the model architecture. In figure 1, the green dot lines show the feature feedback process. However, there forms a cycle in the data flow and this will lead to collapse in gradient descent. The authors are suggested to provide more explanation about how to solve this problem in the training/fine-tuning process or provide clearer dataflow of the architecture.

2. The second concern is about the input of the framework. Since there are images with different resolutions serving as input. In figure 1, it seem that the left input images for C1 are the super-resolutioned version or a patch of the input images on the right for P1. Thus, the problem is where does the author obtain or how to decide which part should be extracted to perform as these image patchs? The authors are suggested to give more explaination about the image pre-processing part.

3. In this paper, both rice plant diseases dataset v8 and v9 are from Roboflow, likely lab-collected images, which are limited to some specified regions or type of rice plants. Real-world field conditions (motion blur, occlusion, varying lighting, mixed diseases) may not be adequately represented. Different regions may also face different diseases. Thus, the generalizability of the proposed method is not adequatly investigated and it is suggested to include more real-world datasets and use cross-domain evaluation (train on v8 and test on real-world images).

4. The manuscript suffers from numerous writing flaws.

(1) In the amnusript, all abbreviations and their full forms should be defined at first use. But there are several abbreviations whoes full forms are not provided, e.g. MCCA-YOLO, what does MCCA mean?; C2F; and some of the abbreviations used in evaluation metrics tables cannot be found in previous contents etc.

(2) The evaluation metrics are not properly formatted. e.g. In Table 7, why 0.895 is presented in bold format? The results of MCCA-YOLO are not the same if Table 9 and Table 15. The table title of Table 16 should be v8.

(3) The images are not positioned with their titles in pdf file and the hypper ref link of these images lead to titles without images, which makes the paper hard to read.

It is suggested the authors check carefully about the content and fix these problems.

Reviewer #3: 1.Lack of ablation experiments for TSSFE in Backbone based on rice plant diseases dataset v8.

2.Check the use and interpretation of the α variable in the formula.

3.There are several details in the paper, such as inconsistent variable formats, layout of formulas, the lack of punctuation.

4.Check the lowercase at the beginning of paragraphs and the details of the text in the tables.

5.The DA module should have detailed illustrations.

6.The flowchart should have appropriate annotations, such as the experimental categories shown in Figure 9, the input and output of the flowchart.

**********

6. PLOS authors have the option to publish the peer review history of their article (what does this mean?). If published, this will include your full peer review and any attached files.

If you choose “no”, your identity will remain anonymous but your review may still be made public.

**Do you want your identity to be public for this peer review?**    For information about this choice, including consent withdrawal, please see our Privacy Policy.

Reviewer #1: No

Reviewer #2: No

Reviewer #3: No

**********

[NOTE: If reviewer comments were submitted as an attachment file, they will be attached to this email and accessible via the submission site. Please log into your account, locate the manuscript record, and check for the action link "View Attachments". If this link does not appear, there are no attachment files.]

To ensure your figures meet our technical requirements, please review our figure guidelines: https://journals.plos.org/plosone/s/figures

You may also use PLOS’s free figure tool, NAAS, to help you prepare publication quality figures: https://journals.plos.org/plosone/s/figures#loc-tools-for-figure-preparation.

NAAS will assess whether your figures meet our technical requirements by comparing each figure against our figure specifications.

Attachment Submitted filename: PONE-D-25-58819-review.docx

# Author comment: Author response to Decision Letter 1

**DOI:** 10.1371/journal.pone.0351727.r002

2 Feb 2026

Revision Report:

Article title: Multi-scale closed-loop tuning via spatial-frequency collaborative sensitivity for rice leaf disease detection

Dear reviewer,

We would like to express our sincere gratitude to the reviewer for the encouraging comments and the recognition of our work's value in agricultural disease diagnosis. We have carefully considered the three suggestions regarding image layout, comparative visuals, and manuscript length. We have revised the manuscript accordingly. The revised texts are marked in red in the manuscript. Please find our point-by-point responses below.

Reviewer 1:

1) Comment 1: Image Layout Misalignment: Several images in the paper exhibit misaligned layouts, which compromises visual clarity and may distract readers from key findings.

Response: We apologize for the oversight regarding the image layout in the original submission. Thank you for pointing this error. We have professionally reformatted all figures in the revised manuscript to ensure proper alignment, consistent margins, and high visual clarity. Specifically, we have adjusted Figure to correct the layout issues. We have also checked the final PDF to ensure that text and figures are correctly positioned and do not overlap.

The PLOS ONE system requires figures to be uploaded as individual files, and they appear as such in the auto-generated review PDF. They are typically compiled and placed at the end of the document by default rather than displayed next to their captions in the main text. As a result, captions may appear in the manuscript body while the corresponding images are collected at the bottom, and the hyperlinks may direct to the caption location instead of an in-text embedded figure. To help readers navigate the manuscript more easily, we have checked that all figures are clearly and consistently cited in the text (e.g., “Fig X”) and that each caption matches the correct uploaded figure file.

2) Comment 2: Insufficient Comparative Visuals: While quantitative results are compelling, additional images illustrating detection effects across diverse datasets would better demonstrate the model’s robustness and real-world applicability.

Response: We sincerely appreciate the reviewer’s valuable suggestion, we agree that visual comparisons are crucial for demonstrating the model's robustness. To address this, we have added three new figures (Figure [8][9][10]) in the revised manuscript. This figure presents a qualitative comparison between our MCCA-YOLO and the baseline model (YOLOv8s) as well as other state-of-the-art models (e.g., Mamba YOLO) on challenging samples from both the Rice Plant Diseases v8 dataset ,Rice Plant Diseases v9 dataset,and RLSD dataset. These samples include scenarios with complex backgrounds, small early-stage lesions, and variable lighting conditions. As shown in figure [8][9][10], our model demonstrates superior localization accuracy and a lower false-negative rate compared to other methods, visually confirming the quantitative results presented in Table 19, Table 20 and Table 21, as below:

The following paragraph has been added to section 4.3 (on page 25).

“To further evaluate the robustness and cross-dataset generalization of MCCA-YOLO, we conduct a qualitative comparison on three datasets: Rice Plant Diseases v9, Rice Plant Diseases v8, and RLSD. Fig 8, Fig 9, and Fig 10 present representative cases with typical challenges, including tiny lesions with low contrast (Rice Blast), elongated disease regions in cluttered backgrounds (Bacterial Leaf Blight), and large-area symptoms with ambiguous boundaries (Leaf Scald). We compare MCCA-YOLO with several competitive detectors (YOLOv5s, YOLOv8s, BGF-YOLO, RT-DETR, and Mamba YOLO) under the same visualization settings. As shown in Fig 8, the rice blast lesion is small and visually similar to surrounding textures, which often leads to imprecise localization or missed detections for baseline models. In Fig 9, bacterial leaf blight exhibits long stripe-like patterns across leaves, competing methods tend to produce fragmented predictions, duplicated boxes, or over-extended boxes covering irrelevant regions. In contrast, MCCA-YOLO yields more consistent localization with tighter bounding boxes around the actual diseased areas. For RLSD in Fig 10, where symptoms cover a relatively large region and the boundary is weak, several methods either under-localize or over-localize, whereas MCCA-YOLO better balances completeness and precision. Overall, these visual results indicate that MCCA-YOLO is more robust to variations in scale, appearance, and background complexity, demonstrating strong generalization across different agricultural datasets.”

Fig 8. Visualization of detection results for Dataset V9.

Fig 9. Visualization of detection results for Dataset V8.

Fig 10. Visualization of detection results for the RLSD Dataset.

3) Comment 3: Excessive Length: The paper is overly lengthy; non-innovative details (e.g., routine method descriptions or standard experimental setup steps) should be relocated to supplementary materials to streamline the main text and enhance focus.

Response: We greatly appreciate the reviewer’s suggestion to improve readability. We strictly followed this suggestion to streamline the manuscript. We have made the following major revisions:

1.Removed Routine Descriptions: We deleted the detailed description of the standard YOLOv8 detection head (original submission Section 3.6) and condensed the background descriptions of the datasets (Section 4.1). To enhance the generalization capability of the model, we have additionally incorporated an RLSD dataset for rice leaf pathology analysis. The updates have been highlighted in red color in the manuscript, as below:

The following paragraph has been updated to section 4.1 (on page 17).

“This study utilizes the Rice Plant Diseases v9, Rice Plant Diseases v8 datasets,and Rice Leaf Spot Disease Dataset, labeled by agricultural specialists, aimed at offering varied visual data for model training and assessment.The images are field-captured RGB color images, resized to 640×640 px. To ensure uniformity and compatibility with the model, normalization was applied during the preprocessing stage. The Rice Plant Diseases v9 and Rice Plant Diseases v8 datasets cover four rice leaf diseases: Bacterial leaf blight, Grassy stunt, Rice blast, and Tungro, the Rice Leaf Spot Disease Dataset covers eight rice leaf classes: Bacterial leaf blight(BLB), Brown Spot, Healthy,

Leaf Blast, Leaf Scald, Leaf Spot, Neck Blast, and Rice Hispa, all of which display unique visual signatures essential for computer vision tasks. These annotated features are critical for training models to accurately classify and assess the severity of different rice leaf diseases.”

The following paragraph has been updated to section 4.1 (on page 17).

“Table 3 displays the Rice Leaf Spot Disease Dataset (RLSD), which is employed in this research to evaluate model generalization across a diverse range of disease categories. The dataset contains a total of 3567 images (6122 annotations) distributed across eight distinct classes. The training dataset includes 531 labels for BLB, 1148 for Brown Spot, 662 for Healthy samples, 634 for Leaf Blast, 452 for Leaf Scald, 572 for Leaf Spot, 770 for Neck Blast, and 697 for Rice Hispa. Meanwhile, the validation dataset comprises 58 labels for BLB, 128 for Brown Spot, 76 for Healthy samples, 63 for Leaf Blast, 60 for Leaf Scald, 66 for Leaf Spot, 100 for Neck Blast, and 105 for Rice Hispa.”

2.Relocated Experimental Setup: We moved the detailed Hardware/Software Configuration Tables and the Hyperparameter Settings Table from the main text to the Supplementary Materials (Supporting information), as below:

Supporting Information file has a legend listed in the manuscript after the references list. The following paragraph has been updated to section Supporting information on page 32.

“Supporting information

S1 Table. Hardware configuration

S2 Table. Software configuration.

S3 Table. Key hyperparameter settings.”

supporting information files

3.Simplified Metrics: We removed the standard mathematical formulas for Precision, Recall, and mAP in original Section 4.3. These changes have significantly reduced the length of the main text and improved the focus on our proposed core innovations.

Revision Report:

Article title: Multi-scale closed-loop tuning via spatial-frequency collaborative sensitivity for rice leaf disease detection

Dear reviewer,

We sincerely appreciate the reviewer for thorough assessment and positive comments regarding the innovation and empirical validation of our work. We have carefully addressed the concerns regarding the model architecture, input preprocessing, generalization, and writing quality. Please find our detailed responses below.

Reviewer 2:

1)Comment 1:The first concern is about the model architecture. In figure 1, the green dot lines show the feature feedback process. However, there forms a cycle in the data flow and this will lead to collapse in gradient descent. The authors are suggested to provide more explanation about how to solve this problem in the training/fine-tuning process or provide clearer dataflow of the architecture.

Response: We sincerely appreciate the reviewer’s valuable suggestion, thank you for pointing out the potential ambiguity of the feedback connections in Fig 1. We agree that a cyclic computational graph could cause instability during backpropagation. In our implementation, the proposed “closed-loop” tuning is not realized as a true cyclic graph. Instead, it is implemented as a two-pass (unrolled) refinement with a stop-gradient (SG) operation on the feedback features, which prevents gradients from flowing through the feedback path and therefore avoids any cyclic dependency in gradient descent.

Specifically, we first perform a standard forward pass to obtain the multi-scale semantic outputs

{OS,OM,OL}. These outputs are then detached (stop-gradient) and only used as guidance signals to calibrate the auxiliary shallow features in the subsequent refinement step. The detection loss is computed on the final predictions, while the first-pass outputs are used solely for feature guidance. To improve clarity, we have revised the relevant paragraph in the updated version. The modified text can now be found on page 13-15 in section 3.4.

We have revised Fig 1 and Fig 5 by explicitly marking the feedback edges with “SG” to indicate stop-gradient on{OS,OM,OL}, clarifying that the feedback is guidance-only and does not form a cyclic backpropagation graph.

Expanded Section 3.4 (CLTB) with a clearer description of the training/inference dataflow, and added pseudocode (Algorithm 1) to present the unrolled two-step procedure and the exact locations where SG is applied.

These revisions clarify that the feedback mechanism does not create a gradient loop and thus does not lead to collapse in gradient descent.

Fig 1. MCCA-YOLO model architecture

Fig 5. Closed-loop tuning Bi-backbone network

The following paragraph has been updated to section 3.4 (on page 13-15).

2)Comment 2: The second concern is about the input of the framework. Since there are images with different resolutions serving as input. In figure 1, it seem that the left input images for C1 are the super-resolutioned version or a patch of the input images on the right for P1. Thus, the problem is where does the author obtain or how to decide which part should be extracted to perform as these image patchs? The authors are suggested to give more explaination about the image pre-processing part.

Response: We apologize for any ambiguity in the original manuscript that may have led to misunderstanding. We appreciate the reviewer for pointing out this ambiguity. The input to our framework is uniform. We do not use super-resolution or separate patch extraction algorithms as input preprocessing. The "patches" illustrated in the original figure were intended to four distinct categories of disease, not distinct input crops.

Revision: In the revised Figure 1, the diagram now displays representative sample images corresponding to the four distinct disease categories (e.g., Bacterial leaf blight, Blast, etc.) as the network input. This visualization serves to illustrate the diversity of disease types handled by the model, rather than a patch-based preprocessing step. We have also added a clear description in As noted in Section 4.1, we apply only standard resizing and normalization as preprocessing.

Fig 1. MCCA-YOLO model architecture

The following paragraph has been added to section 4.1 (on page 17).

“The images are field-captured RGB color images, resized to 640×640 px. To ensure uniformity and compatibility with the model, normalization was applied during the preprocessing stage.”

3) Comment 3: In this paper, both rice plant diseases dataset v8 and v9 are from Roboflow, likely lab-collected images, which are limited to some specified regions or type of rice plants. Real-world field conditions (motion blur, occlusion, varying lighting, mixed diseases) may not be adequately represented. Different regions may also face different diseases. Thus, the generalizability of the proposed method is not adequatly investigated and it is suggested to include more real-world datasets and use cross-domain evaluation (train on v8 and test on real-world images).

Response: We sincerely regret the oversight in the original submission. Thank you for pointing outthis error. As suggested, we strictly followed the reviewer’s suggestion to rigorously validate the model's generalizability and robustness in real-world field conditions. We have made comprehensive revisions in the following aspects:

1. First, although the Rice Plant Diseases v8 and v9 datasets are hosted on Roboflow, the images contained within them were indeed captured in real-world field environments, rather than controlled laboratory settings. However, we fully agree with the reviewer that broader cross-domain evaluation is necessary to prove the model's robustness against varying conditions.

2. Inclusion of the RLSD Dataset: To address the concern about "limited regions" and "complex field conditions," we have expanded our evaluation to include the RLSD (Real-life Rice Leaf Diseases) dataset. This dataset features images collected in complex field environments with diverse backgrounds. We have updated Table 3 to include the detailed description of the RLSD dataset, as below:

The following paragraph has been updated to section 4.1 (on page 18).

“Table 3 displays the Rice Leaf Spot Disease Dataset (RLSD), which is employed in this research to evaluate model generalization across a diverse range of disease categories. The dataset contains a total of 3567 images (6122 annotations) distributed across eight distinct classes. The training dataset includes 531 labels for BLB, 1148 for Brown Spot, 662 for Healthy samples, 634 for Leaf Blast, 452 for Leaf Scald, 572 for Leaf Spot, 770 for Neck Blast, and 697 for Rice Hispa. Meanwhile, the validation dataset comprises 58 labels for BLB, 128 for Brown Spot, 76 for Healthy samples, 63 for Leaf Blast, 60 for Leaf Scald, 66 for Leaf Spot, 100 for Neck Blast, and 105 for Rice Hispa.”

3. Comprehensive Cross-Domain Experiments: We have conducted extensive quantitative experiments on the RLSD dataset to verify generalizability:

Ablation Studies: We added results on the RLSD dataset to Table 6, Table 9, and Table 12, confirming the effectiveness of our proposed modules (TSSFE, DHCA, etc.) across different domains.

Comparative Experiments: We extended the comparison with state-of-the-art models to include the RLSD dataset, as presented in Table 15, Table 18, and Table 21, as shown below. The results demonstrate that MCCA-YOLO maintains superior performance even on this new, challenging dataset.

4.Qualitative Analysis on Unseen Data: We have added a new figure [8][9][10] to present a cross-domain qualitative analysis. This analysis utilizes images from the test set (which the model had never seen during training) to visually demonstrate that MCCA-YOLO maintains high-level detection capabilities in unfamiliar environments with complex backgrounds.

Attachment Submitted filename: Response to Reviewers.docx

# Editor report: Decision Letter 1

**DOI:** 10.1371/journal.pone.0351727.r003

**Authors:** Yun Zhang

9 Feb 2026

PONE-D-25-58819R1

Multi-scale closed-loop tuning via spatial-frequency collaborative sensitivity for rice leaf disease detection

PLOS One

Dear Dr. An,

Thank you for submitting your manuscript to PLOS ONE. After careful consideration, we feel that it has merit but does not fully meet PLOS ONE’s publication criteria as it currently stands. Therefore, we invite you to submit a revised version of the manuscript that addresses the points raised during the review process.

Please submit your revised manuscript by Mar 26 2026 11:59PM. If you will need more time than this to complete your revisions, please reply to this message or contact the journal office at plosone@plos.org. When you're ready to submit your revision, log on to https://www.editorialmanager.com/pone/ and select the 'Submissions Needing Revision' folder to locate your manuscript file.

Please include the following items when submitting your revised manuscript:

- A letter that responds to each point raised by the academic editor and reviewer(s). You should upload this letter as a separate file labeled 'Response to Reviewers'.
- A marked-up copy of your manuscript that highlights changes made to the original version. You should upload this as a separate file labeled 'Revised Manuscript with Track Changes'.
- An unmarked version of your revised paper without tracked changes. You should upload this as a separate file labeled 'Manuscript'.

If you would like to make changes to your financial disclosure, please include your updated statement in your cover letter. Guidelines for resubmitting your figure files are available below the reviewer comments at the end of this letter.

If applicable, we recommend that you deposit your laboratory protocols in protocols.io to enhance the reproducibility of your results. Protocols.io assigns your protocol its own identifier (DOI) so that it can be cited independently in the future. For instructions see: https://journals.plos.org/plosone/s/submission-guidelines#loc-laboratory-protocols. Additionally, PLOS ONE offers an option for publishing peer-reviewed Lab Protocol articles, which describe protocols hosted on protocols.io. Read more information on sharing protocols at https://plos.org/protocols?utm_medium=editorial-email&utm_source=authorletters&utm_campaign=protocols.

We look forward to receiving your revised manuscript.

Kind regards,

Yun Zhang

Academic Editor

PLOS One

Journal Requirements:

If the reviewer comments include a recommendation to cite specific previously published works, please review and evaluate these publications to determine whether they are relevant and should be cited. There is no requirement to cite these works unless the editor has indicated otherwise.

Additional Editor Comments:

The revision is well prepared, but all the figures are missing, please re-organize and check your manuscript carefully. Then submit it again.

There are too many tables in this paper, I hope author reduce some of them, or at least put some of them to the appendix.

[Note: HTML markup is below. Please do not edit.]

[NOTE: If reviewer comments were submitted as an attachment file, they will be attached to this email and accessible via the submission site. Please log into your account, locate the manuscript record, and check for the action link "View Attachments". If this link does not appear, there are no attachment files.]

To ensure your figures meet our technical requirements, please review our figure guidelines: https://journals.plos.org/plosone/s/figures

You may also use PLOS’s free figure tool, NAAS, to help you prepare publication quality figures: https://journals.plos.org/plosone/s/figures#loc-tools-for-figure-preparation.

NAAS will assess whether your figures meet our technical requirements by comparing each figure against our figure specifications.

# Author comment: Author response to Decision Letter 2

**DOI:** 10.1371/journal.pone.0351727.r004

12 Apr 2026

Revision Report:

Article title: Multi-scale closed-loop tuning via spatial-frequency collaborative sensitivity for rice leaf disease detection

Dear reviewer,

We would like to express our sincere gratitude to the reviewer for the encouraging comments and the recognition of our work's value in agricultural disease diagnosis. We have carefully considered the three suggestions regarding image layout, comparative visuals, and manuscript length. We have revised the manuscript accordingly. The revised texts are marked in red in the manuscript. Please find our point-by-point responses below.

Reviewer 1:

1) Comment 1: Image Layout Misalignment: Several images in the paper exhibit misaligned layouts, which compromises visual clarity and may distract readers from key findings.

Response: We apologize for the oversight regarding the image layout in the original submission and appreciate the reviewer’s attention to this issue. In the marked-up copy of our manuscript (labeled “Revised Manuscript with Track Changes”), we have professionally reformatted all figures to ensure proper alignment, consistent margins, and high visual clarity. Specifically, we adjusted all figures to correct the layout issues and verified the final PDF to confirm that text and figures are correctly positioned without overlap.

Regarding the unmarked version of the revised manuscript (labeled “Manuscript”), as required by the PLOS ONE system, figures must be uploaded as separate files. Consequently, we removed all figures from the manuscript file, retaining only the individual TIFF/EPS image files. In the auto-generated review PDF, these images are typically compiled and placed at the end of the document by default, rather than appearing next to their corresponding captions in the main text. As a result, figure captions remain in the manuscript body while the images are grouped at the bottom, hyperlinks may also direct to the caption location instead of to an embedded figure within the text.

2) Comment 2: Insufficient Comparative Visuals: While quantitative results are compelling, additional images illustrating detection effects across diverse datasets would better demonstrate the model’s robustness and real-world applicability.

Response: We sincerely appreciate the reviewer’s valuable suggestion, we agree that visual comparisons are crucial for demonstrating the model's robustness. To address this, we have added three new figures (Figure [8][9][10]) in the revised manuscript. This figure presents a qualitative comparison between our MCCA-YOLO and the baseline model (YOLOv8s) as well as other state-of-the-art models (e.g., Mamba YOLO) on challenging samples from both the Rice Plant Diseases v8 dataset ,Rice Plant Diseases v9 dataset,and RLSD dataset. These samples include scenarios with complex backgrounds, small early-stage lesions, and variable lighting conditions. As shown in figure [8][9][10], our model demonstrates superior localization accuracy and a lower false-negative rate compared to other methods, visually confirming the quantitative results presented in Table 6, as below:

The following paragraph has been added to section 4.3 (on page 26).

“To further evaluate the robustness and cross-dataset generalization of MCCA-YOLO, we conduct a qualitative comparison on three datasets: Rice Plant Diseases v9, Rice Plant Diseases v8, and RLSD. Fig 8, Fig 9, and Fig 10 present representative cases with typical challenges, including tiny lesions with low contrast (Rice Blast), elongated disease regions in cluttered backgrounds (Bacterial Leaf Blight), and large-area symptoms with ambiguous boundaries (Leaf Scald). We compare MCCA-YOLO with several competitive detectors (YOLOv5s, YOLOv8s, BGF-YOLO, RT-DETR, and Mamba YOLO) under the same visualization settings. As shown in Fig 8, the rice blast lesion is small and visually similar to surrounding textures, which often leads to imprecise localization or missed detections for baseline models. In Fig 9, bacterial leaf blight exhibits long stripe-like patterns across leaves, competing methods tend to produce fragmented predictions, duplicated boxes, or over-extended boxes covering irrelevant regions. In contrast, MCCA-YOLO yields more consistent localization with tighter bounding boxes around the actual diseased areas. For RLSD in Fig 10, where symptoms cover a relatively large region and the boundary is weak, several methods either under-localize or over-localize, whereas MCCA-YOLO better balances completeness and precision. Overall, these visual results indicate that MCCA-YOLO is more robust to variations in scale, appearance, and background complexity, demonstrating strong generalization across different agricultural datasets.”

Fig 8. Comparison of the visualization results between our model and other models on dataset V9.

Fig 9. Comparison of the visualization results between our model and other models on dataset V8.

Fig 10. Comparison of the visualization results between our model and other models on dataset RLSD.

3) Comment 3: Excessive Length: The paper is overly lengthy; non-innovative details (e.g., routine method descriptions or standard experimental setup steps) should be relocated to supplementary materials to streamline the main text and enhance focus.

Response: We greatly appreciate the reviewer’s suggestion to improve readability. We strictly followed this suggestion to streamline the manuscript. We have made the following major revisions:

1.Removed Routine Descriptions: We deleted the detailed description of the standard YOLOv8 detection head (original submission Section 3.6) and statistical metrics (original submission Section 4.3), and condensed the background descriptions of the datasets (original submission Section 4.1) and training setting (original submission Section 4.2). To enhance the generalization capability of the model, we have additionally incorporated an RLSD dataset for rice leaf pathology analysis. The updates have been highlighted in red color in the manuscript, as below:

The following paragraph has been updated to section 4.1 (on page 19-20).

“4.1. Experimental Setup

Datasets. We use the Rice Plant Diseases v9, Rice Plant Diseases v8, and Rice Leaf Spot Disease datasets to provide diverse visual data for model training and evaluation. All images are field-collected RGB color images, which were resized to 640×640 pixels and normalized during preprocessing to ensure data uniformity and model compatibility. The Rice Plant Diseases v9 and Rice Plant Diseases v8 datasets cover four categories of rice leaf diseases: Bacterial Leaf Blight, Grassy Stunt, Rice Blast, and Tungro. In contrast, the Rice Leaf Spot Disease Dataset encompasses eight classes: Bacterial Leaf Blight (BLB), Brown Spot, Healthy, Leaf Blast, Leaf Scald, Leaf Spot, Neck Blast, and Rice Hispa. We randomly split them to train set and test set in a 9:1 ratio. These categories, each with distinct visual characteristics, are crucial for computer vision tasks. The annotations provided are key for training models to classify different rice leaf diseases and assess their severity accurately. These categories, each with distinct visual characteristics, are crucial for computer vision tasks. The annotations provided are key for training models to classify different rice leaf diseases and assess their severity accurately.

Experimental Settings. The computational setup comprises an AMD EPYC 9754 CPU paired with an NVIDIA RTX 4090 GPU. The software stack runs on Ubuntu 20.04 LTS, with Python 3.10 and PyTorch 2.1.0 as core framework, alongside OpenCV 4.11.0 for image preprocessing and augmentation. We employed the Adam optimizer to train the MCCA-YOLO model, with parameters beta1 and beta2 set to 0.9 and 0.999, respectively. The learning rate was initialized at 0.001 ,and a batch size of 16 was used. A weight decay of 0.0005 was applied for regularization to prevent overfitting. All input images were resized to 640×640 pixels. We adopted an early stopping strategy with a patience of 50 epochs to halt training if the validation performance ceased to improve, limiting the total number of epochs to 150.”

2.Relocated Experimental Setup: We moved the detailed Datasets, Hardware/Software Configuration Tables and the Hyperparameter Settings Table from the main text to the Supplementary Materials (Supporting information), as below:

Supporting Information file has a legend listed in the manuscript after the references list. The following paragraph has been updated to section Supporting information on page 33.

“Supporting information

S1 Table. Arrangement of the rice plant diseases v9 dataset.

S2 Table. Arrangement of the rice plant diseases v8 dataset.

S3 Table. Arrangement of the rice plant diseases RLSD dataset.

S4 Table. Hardware configuration.

S5 Table. Software configuration.

S6 Table. Key hyperparameter settings.”

supporting information files

3.Simplified Metrics: We removed the standard mathematical formulas for Precision, Recall, and mAP in original Section 4.3. These changes have significantly reduced the length of the main text and improved the focus on our proposed core innovations.

Revision Report:

Article title: Multi-scale closed-loop tuning via spatial-frequency collaborative sensitivity for rice leaf disease detection

Dear reviewer,

We sincerely appreciate the reviewer for thorough assessment and positive comments regarding the innovation and empirical validation of our work. We have carefully addressed the concerns regarding the model architecture, input preprocessing, generalization, and writing quality. Please find our detailed responses below.

Reviewer 2:

1)Comment 1:The first concern is about the model architecture. In figure 1, the green dot lines show the feature feedback process. However, there forms a cycle in the data flow and this will lead to collapse in gradient descent. The authors are suggested to provide more explanation about how to solve this problem in the training/fine-tuning process or provide clearer dataflow of the architecture.

Response: We sincerely appreciate the reviewer’s valuable suggestion, thank you for pointing out the potential ambiguity of the feedback connections in Fig 1. We agree that a cyclic computational graph could cause instability during backpropagation. In our implementation, the proposed “closed-loop” tuning is not realized as a true cyclic graph. Instead, it is implemented as a two-pass (unrolled) refinement with a stop-gradient (SG) operation on the feedback features, which prevents gradients from flowing through the feedback path and therefore avoids any cyclic dependency in gradient descent.

Specifically, we first perform a standard forward pass to obtain the multi-scale semantic outputs

{OS,OM,OL}. These outputs are then detached (stop-gradient) and only used as guidance signals to calibrate the auxiliary shallow features in the subsequent refinement step. The detection loss is computed on the final predictions, while the first-pass outputs are used solely for feature guidance. To improve clarity, we have revised the relevant paragraph in the updated version. The modified text can now be found on page 14-17 in section 3.4.

We have revised Fig 1 and Fig 5 by explicitly marking the feedback edges with “SG” to indicate stop-gradient on{OS,OM,OL}, clarifying that the feedback is guidance-only and does not form a cyclic backpropagation graph.

Expanded Section 3.4 (CLTB) with a clearer description of the training/inference dataflow, and added pseudocode (Algorithm 1) to present the unrolled two-step procedure and the exact locations where SG is applied.

These revisions clarify that the feedback mechanism does not create a gradient loop and thus does not lead to collapse in gradient descent.

Fig 1. MCCA-YOLO model architecture

Fig 5. Closed-loop tuning Bi-backbone network

The following paragraph has been updated to section 3.4 (on page 14-17).

2)Comment 2: The second concern is about the input of the framework. Since there are images with different resolutions serving as input. In figure 1, it seem that the left input images for C1 are the super-resolutioned version or a patch of the input images on the right for P1. Thus, the problem is where does the author obtain or how to decide which part should be extracted to perform as these image patchs? The authors are suggested to give more explaination about the image pre-processing part.

Response: We apologize for any ambiguity in the original manuscript that may have led to misunderstanding. We appreciate the reviewer for pointing out this ambiguity. The input to our framework is uniform. We do not use super-resolution or separate patch extraction algorithms as input preprocessing. The "patches" illustrated in the original figure were intended to four distinct categories of disease, not distinct input crops.

Revision: In the revised Figure 1, the diagram now displays representative sample images corresponding to the four distinct disease categories (e.g., Bacterial leaf blight, Blast, etc.) as the network input. This visualization serves to illustrate the diversity of disease types handled by the model, rather than a patch-based preprocessing step. We have also added a clear description in As noted in Section 4.1, we apply only standard resizing and normalization as preprocessing.

Fig 1. MCCA-YOLO model architecture

The following paragraph has been added to section 4.1 (on page 19).

“Datasets. We use the Rice Plant Diseases v9, Rice Plant Diseases v8, and Rice Leaf Spot Disease datasets to provide diverse visual data for model training and evaluation. All images are field-collected RGB color images, which were resized to 640×640 pixels and normalized during preprocessing to ensure data uniformity and model compatibility.”

3) Comment 3: In this paper, both rice plant diseases dataset v8 and v9 are from Roboflow, likely lab-collected images, which are limited to some specified regions or type of rice plants. Real-world field conditions (motion blur, occlusion, varying lighting, mixed diseases) may not be adequately represented. Different regions may also face different diseases. Thus, the generalizability of the proposed method is not adequatly investigated and it is suggested to include more real-world datasets and use cross-domain evaluation (train on v8 and test on real-world images).

Response: We sincerely regret the oversight in the original submission. Thank you for pointing outthis error. As suggested, we strictly followed the reviewer’s suggestion to rigorously validate the model's generalizability and robustness in real-world field conditions. We have made comprehensive revisions in the following aspects:

1. First, although the Rice Plant Diseases v8 and v9 datasets are hosted on Roboflow, the images contained within them were indeed captured in real-world field environments, rather than controlled laboratory settings. However, we fully agree with the reviewer that broader cross-domain evaluation is necessary to prove the model's robustness against varying conditions.

2. Inclusion of the RLSD Dataset: To address the concern about "limited regions" and "complex field conditions," we have expanded our evaluation to include the RLSD (Real-life Rice Leaf Diseases) dataset. This dataset features images collected in complex field environments with diverse backgrounds. We have updated S3 table in Supporting information to include the detailed description of the RLSD dataset, as below:

The following paragraph has been added to the Supporting information (on page 33).

“Supporting information

S1 Table. Arrangement of the rice plant diseases v9 dataset.

S2 Table. Arrangement of the rice plant diseases v8 dataset.

S3 Table. Arrangement of the rice plant diseases RLSD dataset.”

Table 3 displays the Rice Leaf Spot Disease Dataset (RLSD), which is employed in this research to evaluate model generalization across a diverse range of disease categories. The dataset contains a total of 3567 images (6122 annotations) distributed across eight distinct classes. The training dataset includes 531 labels for BLB, 1148 for Brown Spot, 662 for Healthy samples, 634 for Leaf Blast, 452 for Leaf Scald, 572 for Leaf Spot, 770 for

Attachment Submitted filename: Response_to_Reviewers_auresp_2.docx

# Editor report: Decision Letter 2

**DOI:** 10.1371/journal.pone.0351727.r005

**Authors:** Yun Zhang

21 Apr 2026

PONE-D-25-58819R2

Multi-scale closed-loop tuning via spatial-frequency collaborative sensitivity for rice leaf disease detection

PLOS One

Dear Dr. An,

Thank you for submitting your manuscript to PLOS ONE. After careful consideration, we feel that it has merit but does not fully meet PLOS ONE’s publication criteria as it currently stands. Therefore, we invite you to submit a revised version of the manuscript that addresses the points raised during the review process.

I think this manuscript is now significantly strengthened and meets our criteria for publication. I am satisfied with the current depth and scope of the work. At this stage, I hope authors can make a careful proofreading for this paper. Specifically, please focus on polishing the grammar and linguistic expression to ensure the highest professional standard before formal acceptance.

Please submit your revised manuscript by Jun 05 2026 11:59PM. If you will need more time than this to complete your revisions, please reply to this message or contact the journal office at plosone@plos.org. When you're ready to submit your revision, log on to https://www.editorialmanager.com/pone/ and select the 'Submissions Needing Revision' folder to locate your manuscript file.

Please include the following items when submitting your revised manuscript:

- A letter that responds to each point raised by the academic editor and reviewer(s). You should upload this letter as a separate file labeled 'Response to Reviewers'.
- A marked-up copy of your manuscript that highlights changes made to the original version. You should upload this as a separate file labeled 'Revised Manuscript with Track Changes'.
- An unmarked version of your revised paper without tracked changes. You should upload this as a separate file labeled 'Manuscript'.

If you would like to make changes to your financial disclosure, please include your updated statement in your cover letter. Guidelines for resubmitting your figure files are available below the reviewer comments at the end of this letter.

If applicable, we recommend that you deposit your laboratory protocols in protocols.io to enhance the reproducibility of your results. Protocols.io assigns your protocol its own identifier (DOI) so that it can be cited independently in the future. For instructions see: https://journals.plos.org/plosone/s/submission-guidelines#loc-laboratory-protocols. Additionally, PLOS ONE offers an option for publishing peer-reviewed Lab Protocol articles, which describe protocols hosted on protocols.io. Read more information on sharing protocols at https://plos.org/protocols?utm_medium=editorial-email&utm_source=authorletters&utm_campaign=protocols.

As the corresponding author, your ORCID iD is verified in the submission system and will appear in the published article. PLOS supports the use of ORCID, and we encourage all coauthors to register for an ORCID iD and use it as well. Please encourage your coauthors to verify their ORCID iD within the submission system before final acceptance, as unverified ORCID iDs will not appear in the published article. *Only*    the individual author can complete the verification step; PLOS staff *cannot*    verify ORCID iDs on behalf of authors.

We look forward to receiving your revised manuscript.

Kind regards,

Yun Zhang

Academic Editor

PLOS One

Journal Requirements:

If the reviewer comments include a recommendation to cite specific previously published works, please review and evaluate these publications to determine whether they are relevant and should be cited. There is no requirement to cite these works unless the editor has indicated otherwise.

Please review your reference list to ensure that it is complete and correct. If you have cited papers that have been retracted, please include the rationale for doing so in the manuscript text, or remove these references and replace them with relevant current references. Any changes to the reference list should be mentioned in the rebuttal letter that accompanies your revised manuscript. If you need to cite a retracted article, indicate the article’s retracted status in the References list and also include a citation and full reference for the retraction notice.

[Note: HTML markup is below. Please do not edit.]

To ensure your figures meet our technical requirements, please review our figure guidelines: https://journals.plos.org/plosone/s/figures

You may also use PLOS’s free figure tool, NAAS, to help you prepare publication quality figures: https://journals.plos.org/plosone/s/figures#loc-tools-for-figure-preparation.

NAAS will assess whether your figures meet our technical requirements by comparing each figure against our figure specifications.

# Author comment: Author response to Decision Letter 3

**DOI:** 10.1371/journal.pone.0351727.r006

26 May 2026

Revision Report:

Article title: Multi-scale closed-loop tuning via spatial-frequency collaborative sensitivity for rice leaf disease detection

Response to the Academic Editor

Dear Academic Editor,

We sincerely thank you for your positive evaluation of our manuscript and for your valuable suggestion regarding language polishing. In response to your comment, we carefully proofread the entire manuscript and revised the grammar, sentence structure, terminology, and linguistic expression to improve clarity, fluency, and academic professionalism.

Comment:

Please focus on polishing the grammar and linguistic expression to ensure the highest professional standard before formal acceptance.

Response:

Thank you very much for your helpful suggestion. We have carefully revised the manuscript throughout and highlighted all language-related changes in the marked-up version. The main revisions are listed below.

The following text has been revised in the Abstract section on page 1:

"making stable yields and quality improvements vital for food security and sustainable agricultural development."

The following text has been revised in the Abstract section on page 1:

"Early infections of rice leaf diseases often exhibit subtle symptoms"

The following text has been revised in the Abstract section on page 1:

"A Multi-scale closed-loop tuning via spatial frequency collaborative sensitivity (MCCA-YOLO) model has been proposed in this paper with a multiscale closed-loop tuning and spatial frequency collaborative attention mechanism for the early detection and classification of rice crop diseases."

The following text has been revised in the Abstract section on page 1:

"feature adaptation""comprehensive" " achieves a mean average precision" "demonstrates"

The following text has been revised in the Introduction section on page 1:

"Rice is a fundamental cereal crop worldwide, serving as the primary dietary staple for a large portion of the global population."

The following text has been revised in the Introduction section on page 1:

"However, rice plants are highly susceptible to various pathogenic infections during their growth cycle."

The following text has been revised in the Introduction section on page 1:

"degrade"

The following text has been revised in the Introduction section on pages 1–2:

"Timely and accurate detection methods for rice leaf diseases are essential for developing effective management strategies and targeted control measures."

The following text has been revised in the Introduction section on page 2:

"This framework significantly improves both detection accuracy and generalization in grain pest identification tasks." "Li et al."

The following text has been revised in the Introduction section on page 2:

"The first module extracts discriminative features from three distinct scales through Local Binary Pattern (LBP), grayscale, and Histogram of Oriented Gradients (HOG) image representations. The second module hierarchically integrates semantic global and local features."

The following text has been revised in the Introduction section on page 2:

"Existing multi-scale fusion strategies frequently encounter information redundancy and scale conflicts, particularly when processing early-stage subtle symptoms on rice leaves, where symptom manifestations exhibit weak correlations across scales."

The following text has been revised in the Introduction section on page 3:

"The proposed detection network consists of a closed-loop tuning Bi-backbone feature extraction network for system self-verification feedback, and a multi-scale feature weighted and deformable hybrid attention bidirectional feature pyramid fusion network to achieve adaptive extraction and cross-scale fusion of rice leaf lesion features."

The following text has been revised in the Introduction section on page 3:

"enhances the texture and edge details of rice leaves, and improves their feature representation."

The following text has been revised in the Introduction section on page 3:

"The experimental results show that our method outperforms other state-of-the-art approaches."

The following text has been revised in the Introduction section on page 3:

"The model is deployed for inference applications on edge devices."

The following text has been revised in the Introduction section on page 3:

"The remainder of this paper is organized as follows. Section 2 reviews prior research on object detection, attention mechanisms, and rice leaf disease detection. Section 3 presents the proposed MCCA-YOLO detection network in detail, including its overall architecture, deformable hybrid collaborative attention mechanism, two-stage spatial frequency enhancement module, closed-loop tuning dual-backbone network, and scale-weighted fusion network. Section 4 evaluates the algorithm on several rice leaf disease datasets and discusses the experimental results. Finally, Section 5 summarizes the main findings and concludes the paper."

The following text has been revised in Section 2.1 on page 3:

"Representative one-stage detectors include SSD and the YOLO series, while Mask R-CNN is a notable two-stage extension of the Faster R-CNN framework."

The following text has been revised in Section 2.1 on page 4:

"The latter strategy seeks to increase representational capacity not through depth, but via architecture. A prominent example is the Composite Backbone Network (CBNet)."

The following text has been revised in Section 2.1 on page 4:

"These multi-scale features are then fused and enhanced by a neck module to form a more discriminative representation for detection."

The following text has been revised in Section 2.1 on page 4:

"PANet augments FPN with an additional bottom-up path, shortening the information flow between low-level and high-level features and thus improving the representation of small objects."

The following text has been revised in Section 2.2 on page 4:

"They operate by adaptively weighting feature responses, emphasizing informative regions or channels while suppressing less useful ones, which has led to substantial gains in various vision tasks including object detection. The seminal Squeeze-and-Excitation (SE) network models inter-channel dependencies, recalibrating channel-wise feature responses to boost representational capacity."

The following text has been revised in Section 2.2 on page 5:

"To incorporate spatial information, the Convolutional Block Attention Module (CBAM) sequentially applies channel and spatial attention sub-modules."

The following text has been revised in Section 2.2 on page 5:

"To better capture global patterns, researchers have turned to the frequency domain, complementing spatial attention mechanisms."

The following text has been revised in Section 2.2 on page 5:

"Other works explore hybrid attention in different architectures. For instance, the FSTA SNN uses spectral statistics to guide spatial and temporal attention, though it is designed for spiking neural networks and operates at a single scale."

The following text has been revised in Section 2.2 on page 5:

"Furthermore, prevalent attention mechanisms often employ fixed or heuristic-based fusion strategies, failing to dynamically recalibrate feature responses based on the varying characteristics of different lesions and image contexts."

The following text has been revised in Section 2.2 on page 5:

"To overcome these challenges, we propose two core components: a Deformable Hybrid Collaborative Attention (DHCA) mechanism and a Two-Stage Spatial Frequency Enhancement (TSSFE) module."

The following text has been revised in Section 2.2 on page 6:

"These modules form the core of our proposed MCCA-YOLO framework, which is tailored for robust rice leaf disease detection."

The following text has been revised in Section 2.3 on page 6:

"Subsequent work, like that of Sharma et al., applied convolutional neural networks (CNNs) to detect a broader set of rice diseases and pests."

The following text has been revised in Section 2.3 on page 6:

"A notable example is UAV T-YOLO-Rice, a lightweight detector based on Tiny YOLOv4. It reported 86% mAP on several diseases and offered a favorable speed-accuracy trade-off, though its older architecture may not fully leverage recent advances."

The following text has been revised in Section 2.3 on page 6:

"To address these limitations, we propose MCCA-YOLO, an enhanced framework based on YOLOv8. Our primary objective is to achieve superior accuracy while maintaining high computational efficiency suitable for practical deployment. This is achieved through several key innovations integrated into the YOLOv8 architecture, designed specifically to enhance robustness against field complexities such as cluttered backgrounds and varying leaf geometries."

The following text has been revised in Section 3.1 on page 6:

"This study proposes a novel framework named Multi-scale Closed-loop tuning via Collaborative spatial-frequency sensitivity Attention YOLO (MCCA-YOLO) for rice leaf disease detection."

The following text has been revised in Section 3.1 on page 6:

"These feedback features are utilized to calibrate the backbone network. This refinement process involves upsampling the features from the neck network and then applying a 1×1 convolutional projection on the relevant layers."

The following text has been revised in Section 3.1 on page 7:

"Following this closed-loop calibration, the calibrated values C3, C4, and C5 are upsampled, respectively, and then fed into the main backbone through a 1×1 convolutional layer."

The following text has been revised in Section 3.1 on page 7:

"The multi-scale features are then integrated in the Scale-Weighted Fusion Neck. Here, the Scale-Weighted Fusion Entity (SWFE) module performs adaptive weighted fusion of cross-layer features, explicitly learning the contribution weight of each scale to lesion detection."

The following text has been revised in Section 3.1 on page 7:

"This design enables the system to maintain a lightweight architecture while preserving high sensitivity to minor disease manifestations and ensuring robust performance in complex environments."

The following text has been revised in Section 3.2 on page 7:

"When detecting rice leaf diseases in natural settings, the leaves themselves are often deformed due to wind and gravity, exhibiting significant bending and tilting. Lesion spots on leaves also tend to arrange in linear patterns, either along the leaf’s longitudinal axis or transverse to it."

The following text has been revised in Section 3.2 on page 7:

"To address these challenges, we propose a Deformable Hybrid Collaborative Attention (DHCA) mechanism and integrate it into the C2F component of the YOLOv8 feature fusion network, forming a new bottleneck-DHCA module."

The following text has been revised in Section 3.2 on page 7:

"Following the DCN, the module incorporates two parallel branches: the direction-aware attention branch and the channel-self-attention branch.The features from both branches are fused via a learnable gating mechanism. Furthermore, residual connections are incorporated to stabilize gradient flow. This design enables the module to effectively adapt to the diverse shapes of leaves."

The following text has been revised in Section 3.2.1 on page 7:

"Consequently, the sampling grid can adaptively deform to fit the local geometric structure of the input, such as leaf curvature and vein orientation."

The following text has been revised in Section 3.2.1 on page 8:

"Thus, the output feature map A” “ is computed as shown in Equation (1)."

The following text has been revised in Section 3.2.1 on page 8:

"Each modulation coefficient weights the corresponding transformed feature. Since the deformed sampling location” "typically has non-integer coordinates, we use bilinear interpolation to compute its feature value from the four nearest pixels in the input feature map."

The following text has been revised in Section 3.2.2 on page 8:

"Following alignment via DCNv4 to compensate for leaf bending and tilting, the pixel positions in the feature maps are geometrically corrected. However, alignment alone is insufficient to capture the linear distribution of lesions along specific directions. To address this, we introduce a Directional-Attention (DA) module, which processes the aligned features using dedicated filters oriented horizontally, vertically, and diagonally."

The following text has been revised in Section 3.2.3 on page 9:

"This weighting vector is then used to recalibrate the input features, performing channel-wise scaling that can potentially amplify lesion-related features and suppress responses from healthy regions.” "First, two complementary feature representations, AK and AQ, are generated by applying depthwise separable convolutions with different kernel sizes (3×3 and 5×5, respectively) to capture multi-scale context."

The following text has been revised in Section 3.2.3 on page 9:

"AK and AQ are concatenated along the channel dimension and then passed to a two-layer 1×1 convolutional module for attention embedding."

The following text has been revised in Section 3.2.3 on page 9:

"The second 1×1 convolution then expands the channel count to n²C, where n is the side length of a local n×n window. The output of this convolution represents the dynamic weight for each channel within its corresponding n×n local window."

The following text has been revised in Section 3.2.3 on page 10:

"The channel-attention map As is applied to Av through element-wise multiplication, producing the refined features Vweight."

The following text has been revised in Section 3.2.4 on page 10:

"The outputs of the two branches, denoted as Adir and Achannel, are first concatenated. The combined tensor is then fed into a gating function. The gating function consists of a 1×1 convolution followed by a Sigmoid function, thereby producing a gating tensor G."

The following text has been revised in Section 3.2.4 on page 11:

"Finally, the gated fusion of the two branches is added to the original input feature map via a residual connection, producing the final output Y of the DHCA module."

The following text has been revised in Section 3.3 on page 11:

"Under complex field conditions involving variable lighting, shading, and natural heterogeneity, lesions on rice leaves often exhibit a distinct set of characteristics."

The following text has been revised in Section 3.3 on page 11:

"Conventional frameworks that rely solely on spatial convolutions or single-domain frequency attention struggle to capture this complementary information, which limits their performance in early disease detection."

The following text has been revised in Section 3.3 on page 11:

"In the first stage, the input feature map m is reshaped via adaptive pooling to obtain mg, which matches the required dimensions for the subsequent 2D DCT."

The following text has been revised in Section 3.3 on page 11:

"The 2D DCT basis function is defined in formula (15). The resulting frequency-domain representation for the i-th block, denoted as Freqi, is then computed according to formula (16)."

The following text has been revised in Section 3.3 on page 12:

"TSSFE is designed to jointly enhance high-frequency textures and low-frequency contours through a two-stage process of spatial-frequency collaborative modeling and dynamic frequency-band weighting."

The following text has been revised in Section 3.3 on page 12:

"The residual structure essentially circumvents the suppression of original input features. The module is designed to be computationally efficient and can be readily integrated into existing network architectures."

The following text has been revised in Section 3.4 on page 12:

"In rice leaf spot monitoring, traditional single-path convolution methods often struggle to effectively mitigate error propagation."

The following text has been revised in Section 3.4 on page 13:

" as illustrated in formula (23)."

The following text has been revised in Section 3.4 on page 13:

"Consequently, deep semantic analysis in later layers alone cannot fully recover the fine-grained edge information lost in early stages."

The following text has been revised in Section 3.4 on page 13:

"These coarse outputs serve solely as se

Attachment Submitted filename: Response_to_Reviewers_auresp_3.docx

# Editor report: Decision Letter 3

**DOI:** 10.1371/journal.pone.0351727.r007

**Authors:** Yun Zhang

1 Jun 2026

Multi-scale closed-loop tuning via spatial-frequency collaborative sensitivity for rice leaf disease detection

PONE-D-25-58819R3

Dear Dr. An,

We’re pleased to inform you that your manuscript has been judged scientifically suitable for publication and will be formally accepted for publication once it meets all outstanding technical requirements.

Within one week, you’ll receive an e-mail detailing the required amendments. When these have been addressed, you’ll receive a formal acceptance letter and your manuscript will be scheduled for publication.

An invoice will be generated when your article is formally accepted. Please note, if your institution has a publishing partnership with PLOS and your article meets the relevant criteria, all or part of your publication costs will be covered. Please make sure your user information is up-to-date by logging into Editorial Manager at Editorial Manager® and clicking the ‘Update My Information' link at the top of the page. For questions related to billing, please contact billing support.

If your institution or institutions have a press office, please notify them about your upcoming paper to help maximize its impact. If they’ll be preparing press materials, please inform our press team as soon as possible -- no later than 48 hours after receiving the formal acceptance. Your manuscript will remain under strict press embargo until 2 pm Eastern Time on the date of publication. For more information, please contact onepress@plos.org.

Kind regards,

Yun Zhang

Academic Editor

PLOS One

Additional Editor Comments (optional):

Reviewers' comments:

# Editor report: Acceptance letter

**DOI:** 10.1371/journal.pone.0351727.r008

**Authors:** Yun Zhang

PONE-D-25-58819R3

PLOS One

Dear Dr. An,

I'm pleased to inform you that your manuscript has been deemed suitable for publication in PLOS One. Congratulations! Your manuscript is now being handed over to our production team.

At this stage, our production department will prepare your paper for publication. This includes ensuring the following:

* All references, tables, and figures are properly cited

* All relevant supporting information is included in the manuscript submission,

* There are no issues that prevent the paper from being properly typeset

You will receive further instructions from the production team, including instructions on how to review your proof when it is ready. Please keep in mind that we are working through a large volume of accepted articles, so please give us a few days to review your paper and let you know the next and final steps.

Lastly, if your institution or institutions have a press office, please let them know about your upcoming paper now to help maximize its impact. If they'll be preparing press materials, please inform our press team within the next 48 hours. Your manuscript will remain under strict press embargo until 2 pm Eastern Time on the date of publication. For more information, please contact onepress@plos.org.

You will receive an invoice from PLOS for your publication fee after your manuscript has reached the completed accept phase. If you receive an email requesting payment before acceptance or for any other service, this may be a phishing scheme. Learn how to identify phishing emails and protect your accounts at https://explore.plos.org/phishing.

If we can help with anything else, please email us at customercare@plos.org.

Thank you for submitting your work to PLOS ONE and supporting open access.

Kind regards,

PLOS ONE Editorial Office Staff

on behalf of

Dr. Yun Zhang

Academic Editor

PLOS One
